# Synthesis and Cytotoxicity Evaluation of Spirocyclic Bromotyrosine Clavatadine C Analogs

**DOI:** 10.3390/md19070400

**Published:** 2021-07-20

**Authors:** Piyush A. Patel, Tanja Bruun, Polina Ilina, Heidi Mäkkylä, Antti Lempinen, Jari Yli-Kauhaluoma, Päivi Tammela, Paula S. Kiuru

**Affiliations:** 1 Drug Research Program, Division of Pharmaceutical Chemistry and Technology, Faculty of Pharmacy, University of Helsinki, FI-00014 Helsinki, Finland; piyushkumar.patel@helsinki.fi (P.A.P.); tanja.bruun@helsinki.fi (T.B.); antti.lempinen@helsinki.fi (A.L.); jari.yli-kauhaluoma@helsinki.fi (J.Y.-K.); 2 Drug Research Program, Division of Pharmaceutical Biosciences, Faculty of Pharmacy, University of Helsinki, FI-00014 Helsinki, Finland; polina.ilina@helsinki.fi (P.I.); heidi.makkyla@helsinki.fi (H.M.); paivi.tammela@helsinki.fi (P.T.)

**Keywords:** Clavatadine C, spirocyclic bromotyrosines, cytotoxicity, cancer selectivity, marine compounds, melanoma A-375 cell line

## Abstract

Marine-originated spirocyclic bromotyrosines are considered as promising scaffolds for new anticancer drugs. In a continuation of our research to develop potent and more selective anticancer compounds, we synthesized a library of 32 spirocyclic clavatadine analogs by replacing the agmatine, i.e., 4-(aminobutyl)guanidine, side chain with different substituents. These compounds were tested for cytotoxicity against skin cancer using the human melanoma cell line (A-375) and normal human skin fibroblast cell line (Hs27). The highest cytotoxicity against the A-375 cell line was observed for dichloro compound **18** (CC_50_ 0.4 ± 0.3 µM, selectivity index (SI) 2). The variation of selectivity ranged from SI 0.4 to reach 2.4 for the pyridin-2-yl derivative **29** and hydrazide analog of 2-picoline **37**. The structure–activity relationships of the compounds in respect to cytotoxicity and selectivity toward cancer cell lines are discussed.

## 1. Introduction

**Figure 1:**
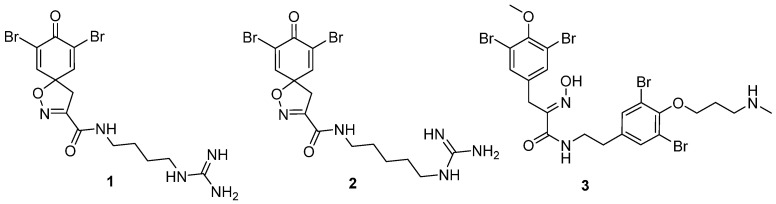
Clavatadine C **1**, D **2**, and purpurealidin I **3**.

Natural products have a long history of use in the treatment of various diseases, including cancer [1]. Marine organisms are a rich source of novel compounds with medicinally relevant properties. Many marine-derived bioactive terpenes, alkaloids, macrolides, and other compounds isolated from aquatic fungi, cyanobacteria, algae, sponges, and tunicates have been found to exhibit anticancer activities [2,3]. To date, 15 drugs with marine origins have been approved by the U.S. Food and Drug Administration and/or European Medicines Agency. Nine of these drugs are registered for the treatment of different cancer types [4,5]. At present, 33 marine-based compounds are in clinical trials, out of which 29 are being evaluated for cancer therapy. Bromotyrosine alkaloids have acquired special importance in medicinal chemistry since the vast majority of these secondary metabolites possess potential anticancer [6], antimicrobial, antiviral, and antifungal activities [7,8]. Quinn and co-workers identified two new spirocyclic bromotyrosine compounds, clavatadine C **1** and clavatadine D **2** (Figure 1). Both were isolated as trifluoroacetic acid (TFA) salts from the marine sponge *Suberea clavata* and their anticoagulative properties were described. [9]. Furthermore, moderate activity against MCF7, MDA-MB-231 (breast), and A549 (lung) cancer cell lines have been observed for clavatadine C **1**-**TFA** [10]. Marine bromotyrosines with an isoxazoline moiety attached to the spiro center have exhibited anticancer properties [11,12]. The spirocyclic bromotyrosine is structurally more rigid and better occupies the chemical space than the open-chain bromotyrosine, making it an interesting scaffold for medicinal chemistry [13]. We have previously reported a set of simplified open-chain bromotyrosine analogs of purpurealidin I **3** (Figure 1) with potential antiproliferative activity [14]. As purpurealidin analogs are *E*-isomers having free rotation around the C-C σ bond, introduction of conformational restriction in the form of a spiro ring fusion offers a good strategy to improve selectivity toward the target cell of interest [15].

**Figure 2:**
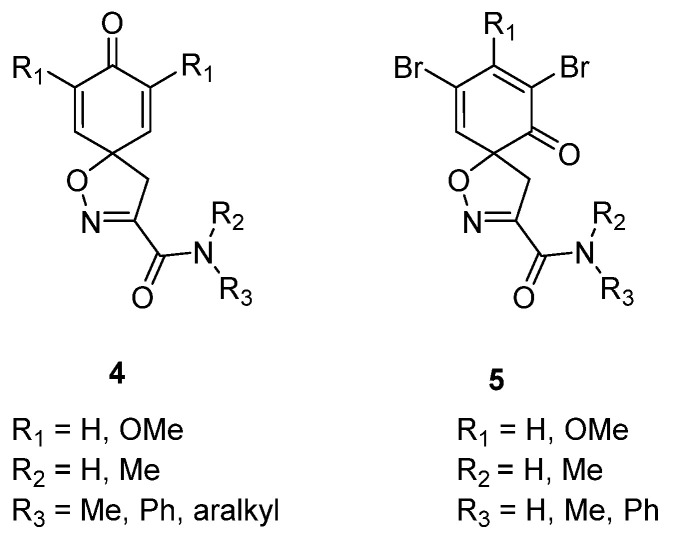
Simplified spiroisoxazoline analogs **4** [17] and **5** [18].

Cytotoxicity toward normal cells is a major challenge with anticancer compounds. Therefore, different approaches are required to develop a target-specific anticancer treatment. The structural simplification of natural products is one of the well-known strategies to improve pharmacokinetic profiles and to reduce side effects [16]. Initially, anticancer activity of the first simplified spiroisoxazolines **4** and **5** (Figure 2) was reported in Ehrlich ascites tumor cells in mice [17,18].

In order to understand the structure–activity relationships (SARs) of spirocyclic bromotyrosines as cytotoxic agents toward cancer cells, we synthesized a library of simplified spirocyclic clavatadine analogs **11**–**42** (Table 1). The agmatine, i.e., 4-(aminobutyl)guanidine, side chain was replaced with different amino and hydrazide substituents. These analogs were tested against a melanoma cell line (A-375) and normal human skin fibroblast cell line (Hs27) for cytotoxicity. The clavatadine scaffold was selected for the library synthesis to limit the free C-C rotation of purpurealidin analogs, to have a stereochemically simpler spiro core than the one in other spirocyclic bromotyrosines, and to build on the proven anticancer activity of simplified clavatadine analogs, such as compound **4**.

**Scheme 1:**
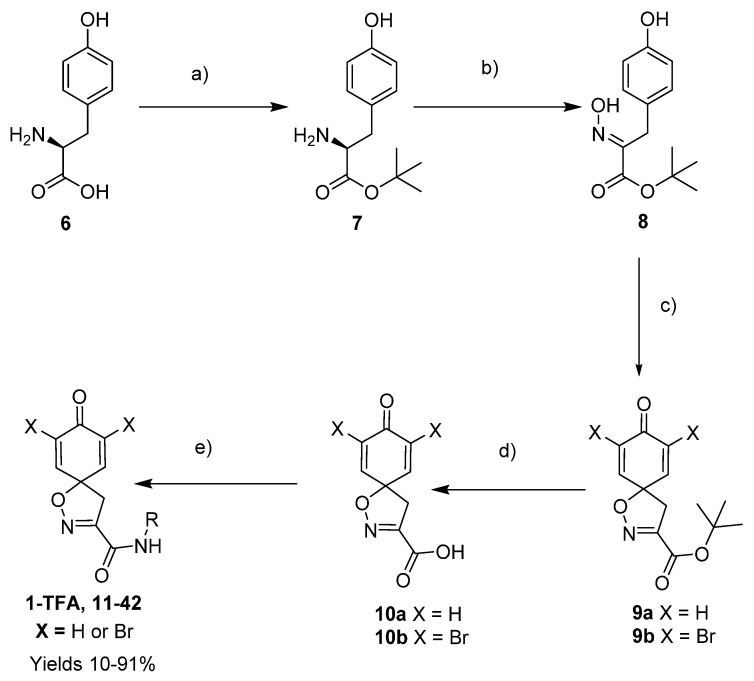
General scheme for synthesis of spirocyclic bromotyrosines **1-TFA** and **11**–**42**. Reagents and conditions: a) HClO_4_, *tert*-butyl acetate, 20–25 °C, 18 h; b) Na_2_WO_4_·2H_2_O, H_2_O_2_, EtOH, 20–25 °C, 7 h; c) PIFA, pyridine, TFE, 0 °C, 1 h, or NBS, DMF, 0 °C, 20 min; d) TFA, DCM, 20–25 °C, 6 h; e) amine, EDC·HCl, HOBt, DCM, 20–25 °C, 5–15 h. The R substituents are given in Table 1.

The compounds were synthesized according to the route presented in Scheme 1. Synthetic procedures and analytical data of the compounds are given in the Supporting Information.

## 2. Results

### 2.1. Chemistry

The synthesis of the spirocyclic bromotyrosine scaffold started with esterification of l-tyrosine **6** using *tert*-butyl acetate in the presence of perchloric acid to give l-tyrosine *tert*-butyl ester **7**. The ester **7** was oxidized with sodium tungstate and H_2_O_2_ to give the oxime **8** [10]. This resulting oxime was subjected to oxidative spirocyclization via treatment with [bis(trifluoro-acetoxy)iodo]benzene (PIFA) in 2,2,2-trifluoroethanol (TFE) in the case of non-halogenated compounds or *N*-bromosuccinimide (NBS) in *N*,*N*-dimethylformamide (DMF) in the case of brominated compounds to provide spirocyclic esters **9a/9b**. The *tert*-butyl esters **9a/9b** were deprotected with trifluoroacetic acid in dichloromethane (DCM) to give the spirocyclic carboxyl core **10a/10b**. This spirocyclic core was coupled with various amines or hydrazides in the presence of 1-ethyl-3-(3-dimethylaminopropyl)carbodiimide hydrochloride (EDC·HCl) and 1-hydroxybenzotriazole hydrate (HOBt⋅H_2_O) to give the target spirocyclic bromotyrosine analogs **1-TFA** and **11**–**42** (Table 1) with yields ranging 10–91%. We observed that the yields were typically higher in the case of dihydro carboxyl core **10a** compared to the dibromo core **10b**, and heterocyclic and aromatic amines compared to aliphatic amines and hydrazines.

### 2.2. Biological Activity

The cytotoxicities of the synthetic clavatadine C **1-TFA**, dihydroclavatadine C **11,** and compounds **12**–**42** against cancer cells were primarily evaluated in the human malignant melanoma A-375 cell line at the single concentration of 50 µM (Table 2). The compounds demonstrating over 80% cytotoxicity were selected for confirmatory dose-response experiments in the same cell line and CC_50_ (cytotoxic concentration that caused death of 50% of cells) was calculated (Table 2). The observed cytotoxicity (CC_50_) against the A-375 melanoma cell line for the compounds **1-TFA** and **11**–**42** was in the range of 0.4–12.3 µM (Table 2). Furthermore, we aimed to evaluate the potential of the compounds to selectively perturb the growth of skin cancer cells. Therefore, the compounds were tested for cytotoxicity in normal human fibroblast cell line Hs27 (Table 2).

**Figure 3:**
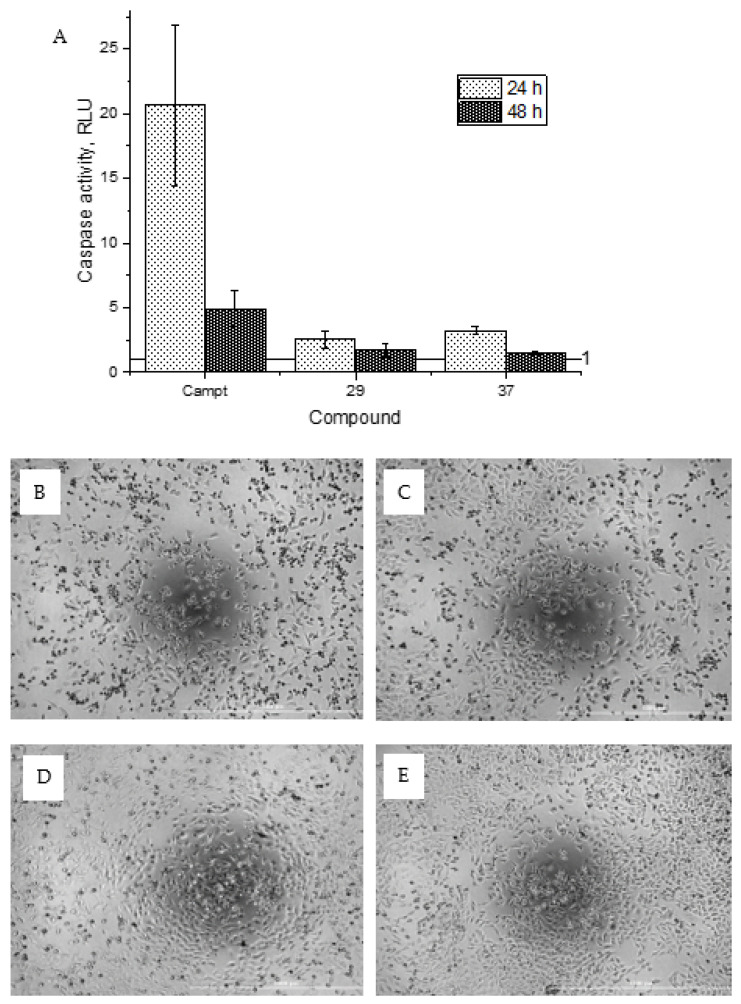
Induction of apoptosis in A-375 melanoma cells by compounds **29** and **37**, which demonstrated the highest selectivity, and positive control camptothecin. Cells were treated with compounds at 1 × CC_50_ concentration for 24 and 48 h. (**A**) Activity of caspase 3/7 apoptotic pathway shown as a fold change relative to DMSO-treated control, indicated with a horizontal line. Bars show mean values of two independent experiments (each performed in triplicate wells) ± SD. Representative microscopic images taken after a 24-h incubation with camptothecin (**B**), compound **29** (**C**), and **37** (**D**) show morphological changes characteristic for apoptosis; (**E**) shows cell morphology in DMSO-treated control wells. RLU = relative light units.

The degree of selectivity toward cancer cells can be expressed by the selectivity index (SI). The SI was calculated as a ratio of CC_50_ values between Hs27 fibroblasts and A-375 melanoma cells. High SI values show selectivity toward cancer cells, while values <2 show low selectivity [19] (Table 2). The highest but still moderate selectivity to cancer cells (SI 2.4, Table 2) was observed for the pyridin-2-yl compound **29** and hydrazide analog of 2-picoline **37**. To further elucidate mechanisms of cytotoxicity mediated by these compounds, we tested their ability to induce apoptosis. Apoptosis, or programmed cell death, is a mechanism utilized in the body for elimination of unwanted or damaged cells during development and aging. In cancer cells, apoptosis is typically inhibited and most selective anticancer agents act via induction of this pathway [20]. To elucidate potential effects of spirocyclic bromotyrosines on apoptosis, we tested their ability to induce the activity of caspases 3/7, a key protease involved in the apoptotic pathway. The results show that spirocyclic bromotyrosines induced the caspase pathway about twofold after 24 h, whereas no induction was observed after 48 h (Figure 3A). Treatment with a positive control camptothecin resulted in about a 20-fold caspase induction after 24 h of treatment and a nearly fivefold induction after 48 h of treatment. The data obtained correlated with microscopic observations. Cell rounding up and shrinkage, typical for apoptosis, was observed for both camptothecin-treated cells and the cells treated with spirocyclic bromotyrosines. However, for camptothecin the effect was more profound (Figure 3B–E).

## 3. Discussion

To understand the preliminary cytotoxicity of clavatadine C **1** analogs comprised of dibromo and dihydro spirocyclic cores, we synthesized clavatadine C **1-TFA** (overall yield 38%) along with various amides **11**–**42** having aliphatic, aromatic, and heterocyclic substitution with or without a carbon spacer. We then evaluated their effect on cytotoxicity of the melanoma cell line A-375 and the normal skin fibroblast cell line Hs27. Though the clavatadine C **1**-**TFA** and its dihydro analog **11** did not show any primary cytotoxicity, its analogs showed cytotoxicity, as shown in Table 2. Clavatadine C **1-TFA,** dihydroclavatadine C **11** and aliphatic morpholinoacetyl carbohydrazide **17** showed less than 25% primary cytotoxicity (at 50 μM in A-375 cells) while the rest of the compounds had over 75% primary cytotoxicity. This result was somewhat unexpected when compared to the known activity of clavatadine C **1-TFA** against breast and colon cancer cell lines [10]. Binnewerg et al. recently found spiro-structured isofistularin-3 to display cell line dependent effects, and the three melanoma cell lines tested (SKMel-147, Mel-Juso, and Malme-3M) showed no significant decrease (and even increased in the case of SKMel-147) in cell viability, but on the contrary isofistularin-3 reduced cell viability of breast cancer cell line MCF-7 [21]. The highest cytotoxicity against the A-375 cell line was observed in dihydro 2,4-dichloro compound **18** (CC_50_ 0.4 ± 0.3 µM, with moderate SI 2). The selectivity indices of the most simplified dimethyl amides **13** and **14** were 1.8 and 1.9, respectively, and amides **14**–**16** containing aliphatic substituents showed low SI. While the introduction of aromatic substituent in **18**–**30** with or without a spacer exhibited relatively similar cytotoxicity, the 3-chloro-4-methoxyphenyl analog **21** showed a slight improvement in the selectivity index. As seen in our open chain library [14], the introduction of pyridinyl substituent **26**–**29** at the amide showed improvement in SI value to 2.4 in the case of pyridin-2-yl analog **29**, whereas the introduction of the pharmacologically important trifluoromethyl group [22] to pyridine in **30** and **31** lowered the selectivity. We also introduced ethylene and methylene groups as spacers in **32**–**36** along with hydrazide spacers in **37**–**39** to pyridine to evaluate their effect on selectivity and cytotoxicity. Cytotoxicity and selectivity were increased in analogs **32**–**36** having a spacer compared to the analogs **14**–**17** having aliphatic substituents. In the case of the dihydro pyridin-2-yl analogs **32** and **37**, the change of spacers to hydrazide **37** led to a modest improvement in selectivity, but in case of the corresponding bromo analogs **33** and **38** the SI was lower in hydrazide **38**. The introduction of other heterocycles **40**–**42** gave similar lower SIs as observed for the aliphatic analogs **14**–**16**. In comparison with our earlier reported open-chain bromotyrosine analogs [14], we found that most spirocyclic bromotyrosine analogs have lower CC_50_ to both cell lines tested.

Overall, the SIs for this set of compounds stayed below 2.5, indicating relatively low selectivity toward A-375 cell line. The cytotoxic effect of the two most selective compounds was partially mediated by caspase-dependent apoptosis, although the low (twofold) level of apoptosis induction at CC_50_ concentration suggests predominantly an unspecific cytotoxicity mechanism. Taking into account considerable dissimilarities in the biology of different cancer types [23], future work may include testing of the compounds in a panel of cancer cell lines representing various malignancies. In summary, biological data of synthesized spiro-structured clavatadine C **1** analogs demonstrate low selectivity toward skin cancer. However, structure–activity relationships indicate further structural optimization by modification of the side chain for potential development of these analogs into anticancer agents should be explored.

## 4. Materials and Methods

### 4.1. Materials and Methods for Biological Testing

Analysis of selectivity to cancer cells. The cells were seeded to white frame and clear bottom 96-well plates (Perkin Elmer) at a density of 10,000 cells/well for the human malignant melanoma A-375 cell line (ATCC CRL-1619) and 7500 cells/well for the human skin fibroblast Hs27 cell line (ATCC CRL-1634). The cells were grown at 37 °C, 5% CO_2_ until they reached 70–80% confluence (approximately 24 h). Stock solutions of test compounds and a positive control (camptothecin, Sigma-Aldrich, St. Louis, MO, USA) were prepared in DMSO and diluted into the assay medium (growth medium with 5% FBS) to the final concentration. Final DMSO concentration was 0.5% in all samples. The culture medium was removed from the plate and compounds added, 200 µL/well. After a 48-h incubation, the amount of ATP, which is directly proportional to the number of viable cells present in culture, was quantified using CellTiter-Glo^®^ Luminescent Cell Viability kit (Promega, Madison, WI, USA), according to manufacturer’s instructions. Origin Graphing and Analysis, version 9.55 (OriginLab, Northampton, MA, USA), was used for determination of CC_50_ values. The cancer cell selectivity index was calculated as a ratio of CC_50_ values between Hs27 fibroblasts and A-375 melanoma cells. Standard deviation of selectivity indices was calculated using Equation (1)

(1) $$\sigma \left(\frac{\bar{x}}{\bar{y}}\right)=\sqrt{{\left(\frac{\sigma \left(x\right)}{\bar{x}}\right)}^{2}+{\left(\frac{\sigma \left(y\right)}{\bar{y}}\right)}^{2}}$$

In the Equation (1), $\bar{x}$ and $\bar{y}$ are average CC_50_ values in Hs27 and A-375 cells, respectively, and σ(x) and σ(y) are their standard deviations.

Apoptosis induction assay and imaging. A-375 cells were seeded to white 96-well plates and treated with compounds at 1 × CC_50_ concentration, 100 µL/well, following the procedure described above. After 24 and 48 h, caspase-3/7 activity was measured using the ApoTox-Glo kit (Promega) following the manufacturer’s instructions. The light microscopy images were taken using 4× phase contrast objective and Cytation5 automated imaging reader (Biotek).

### 4.2. Synthesis Experimental

#### 4.2.1. General

All reactions were carried out using commercially available starting materials unless otherwise stated. The melting points were measured with a Stuart SMP40 automated melting point apparatus and were uncorrected. ^1^H NMR and ^13^C NMR spectra in CDCl_3_, *d*_6_-DMSO, *d*_6_-acetone, or CD_3_OD at ambient temperature were recorded on a Bruker Ascend 400 spectrometer. Chemical shifts (*δ*) are given in parts per million (ppm) relative to the NMR reference solvent signals (CDCl_3_: 7.26 ppm, 77.16 ppm; CD_3_OD: 3.31 ppm, 49.00 ppm; *d*_6_-DMSO: 2.50 ppm, 39.52 ppm; *d*_6_-acetone: 2.05 ppm, 29.84 ppm). Multiplicities are indicated by s (singlet), br s (broad singlet), d (doublet), dd (doublet of doublets), ddd (doublet of doublet of doublets), t (triplet), dt (doublet of triplets), q (quartet), p (pentet), and m (multiplet). The coupling constants *J* were quoted in hertz (Hz). LC-MS and HRMS-spectra were recorded using a Waters Acquity UPLC^®^-system (with Acquity UPLC^®^ BEH C18 column, 1.7 μm, 50 mm × 2.1 mm, Waters, Milford, MA, USA) with Waters Synapt G2 HDMS with the ESI (+), high resolution mode, and PDA. The mobile phase consisted of H_2_O (A) and acetonitrile (B), both containing 0.1% HCOOH. Microwave syntheses were performed in sealed tubes using a Biotage Initiator+ instrument equipped with an external IR sensor. The flash chromatography was performed with a Biotage Isolera One flash chromatography purification system with a 200–800 nm UV-VIS detector using SNAP KP-Sil 10 g, 25 g, or 50 g cartridges. The TLC plates were provided by Merck (Silica gel 60-F254) and visualization of the amine compounds was conducted using ninhydrin (a 0.2% *w*/*v* solution in a 3% solution of acetic acid in 1-butanol) staining.

#### 4.2.2. Experimental Procedures and Characterization Data

***tert*-Butyl** **l-tyrosinate (7)**

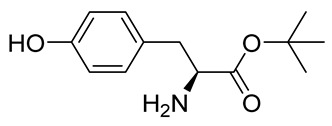


To a stirred suspension of l-tyrosine **6** (25.0 g, 0.138 mol) in *tert*-butyl acetate (100 mL) in an ice bath (0 °C), perchloric acid (15.7 mL, 0.276 mol, 2.0 equiv) was added dropwise. The reaction mixture was allowed to warm to room temperature and was stirred for 17 h. The mixture was washed with H_2_O (300 mL) and a 1 M solution of HCl in H_2_O (250 mL). The aqueous phase was diluted with H_2_O (300 mL), followed by the addition of solid K_2_CO_3_ until the pH was 7. The resulting mixture was filtered, the filtrate was made alkaline (pH 9) by adding solid K_2_CO_3_, and then it was extracted with EtOAc (3 × 300 mL). The combined organic phases were washed with brine (300 mL), dried over anhydrous Na_2_SO_4_, filtered, and concentrated in vacuo to give an off-white solid; crude yield: 29 g (86%). The crude product was purified with automated flash chromatography (DCM/MeOH, gradient: 0→10%) to give the product **7** as a white solid (25 g, 76%). ^1^H NMR (400 MHz, CDCl_3_) *δ* 7.01 (d, *J* = 8.5 Hz, 2H), 6.65 (d, *J* = 8.5 Hz, 2H), 3.60 (dd, *J* = 7.7, 5.3 Hz, 1H), 3.00 (dd, *J* = 13.8, 5.3 Hz, 1H), 2.77 (dd, *J* = 13.8, 7.7 Hz, 1H), 1.45 (s, 9H). ^13^C NMR (101 MHz, CDCl_3_) *δ* 174.2, 155.5, 130.5, 128.2, 115.8, 81.8, 56.2, 40.0, 28.2. ^1^H NMR is in accordance with the literature [24].

***tert*-Butyl (*E*)-2-(hydroxyimino)-3-(4-hydroxyphenyl)propanoate (8)**

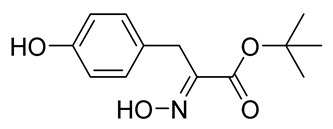


To a stirred solution of *tert*-butyl L-tyrosinate **7** (1.20 g, 5.06 mmol) in EtOH (20 mL) in an ice bath (0 °C), Na_2_WO_4_·2H_2_O (1.83 g, 10.4 mmol, 1.1 equiv), a 30% solution of H_2_O_2_ in H_2_O (9 mL), and H_2_O (14 mL) wereadded. The resulting mixture was stirred for 8 h and slowly allowed to reach room temperature. The mixture was diluted with EtOAc (25 mL) and washed with a 10% solution of Na_2_SO_3_ in H_2_O (2 × 15 mL), H_2_O (2 × 15 mL), and brine (15 mL). The aqueous phase was extracted with EtOAc (3 × 20 mL), and the combined organic phases were washed with brine (10 mL), dried over anhydrous Na_2_SO_4_, filtered, and concentrated in vacuo. The crude product was purified with automated flash chromatography (*n*-heptane/EtOAc gradient: 5→50%) to give the compound **8** as a white solid (1.00 g, 79%). ^1^H NMR (400 MHz, CD_3_OD) *δ* 7.05 (d, *J* = 8.7 Hz, 2H), 6.67 (d, *J* = 8.6 Hz, 2H), 3.79 (s, 2H), 1.43 (s, 9H). ^13^C NMR (101 MHz, CD_3_OD) *δ* 164.6, 156.9, 153.6, 131.0, 128.6, 116.1, 83.3, 30.3, 28.1. ^1^H NMR is in accordance with the literature [10].

***tert*-Butyl 8-oxo-1-oxa-2-azaspiro[4.5]deca-2,6,9-triene-3-carboxylate (9a)**

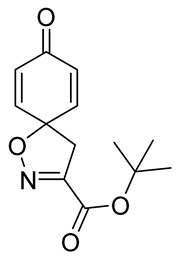


*tert*-Butyl (*E*)-2-(hydroxyimino)-3-(4-hydroxyphenyl)propanoate **8** (0.40 g, 1.6 mmol) was dissolved in 2,2,2-trifluoroethanol (7.7 mL), followed by the addition of anhydrous pyridine (0.39 mL, 4.8 mmol, 2 equiv). The mixture was cooled in an ice bath (0 °C) for 5 min. Phenyliodine bis(trifluoroacetate) (0.76 g, 1.8 mmol, 1.1 equiv) was added to the cooled mixture, and stirring was continued for 1.5 h. The reaction was quenched with a 10% solution of Na_2_S_2_O_3_ in H_2_O (13 mL), and the resulting mixture extracted with EtOAc (3 × 15 mL). The combined organic phases were washed with brine, dried over anhydrous Na_2_SO_4_, filtered, and concentrated in vacuo. The crude product was purified with automated flash chromatography (*n*-heptane/EtOAc gradient: 12→100%) to give the compound **9a** as a yellow oil (0.33 g, 84%). ^1^H NMR (400 MHz, *d*_6_-acetone) *δ* 7.12–7.05 (m, 2H), 6.24–6.18 (m, 2H), 3.48 (s, 2H), 1.52 (s, 9H). ^13^C NMR (101 MHz, *d*_6_-acetone) *δ* 184.9, 159.5, 153.7, 145.7, 129.2, 83.6, 83.6, 44.3, 28.1. ^1^H NMR is in accordance with the literature [10].

**8-Oxo-1-oxa-2-azaspiro[4.5]deca-2,6,9-triene-3-carboxylic acid (10a)**

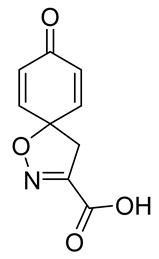


To a solution of *tert*-butyl 8-oxo-1-oxa-2-azaspiro[4.5]deca-2,6,9-triene-3-carboxylate **9a** (0.33 g, 1.3 mmol) in anhydrous DCM (10 mL), trifluoroacetic acid (5.0 mL) was added dropwise. The resulting mixture was stirred at room temperature for 3 h. The solvent was removed in vacuo to give the compound **10a** as a light brown solid (0.24 g, 93%). ^1^H NMR (400 MHz, *d*_6_-acetone) *δ* 7.15–7.08 (m, 1H), 6.26–6.19 (m, 1H), 3.51 (s, 1H). ^13^C NMR (101 MHz, *d*_6_-acetone) *δ* 184.9, 161.0, 153.0, 145.7, 129.3, 116.5, 83.8.

***tert*-Butyl 7,9-dibromo-8-oxo-1-oxa-2-azaspiro[4.5]deca-2,6,9-triene-3-carboxylate (9b)**

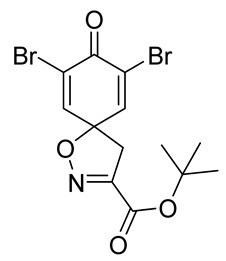


To a solution of *tert*-butyl (*E*)-2-(hydroxyimino)-3-(4-hydroxyphenyl)propanoate **8** (0.59 g, 2.348 mmol) in anhydrous DMF (10 mL) in an ice bath (0 °C), *N*-bromosuccinimide (1.35 g, 7.58 mmol, 3.25 equiv) in anhydrous DMF (5 mL) was added dropwise (15 min). The mixture was diluted with Et_2_O (25 mL), and washed with H_2_O (2 × 15 mL) and a 10% solution of Na_2_S_2_O_3_ in H_2_O (2 × 15 mL). The aqueous phase was back-extracted with Et_2_O (2 × 30 mL). The combined organic phases were washed with brine (20 mL), dried over anhydrous Na_2_SO_4_, filtered, and concentrated in vacuo. The crude product was purified with automated flash chromatography (isocratic DCM) to give the compound **9b** as a white solid (0.58 g, 62%). ^1^H NMR (400 MHz, CDCl_3_) *δ* 7.32 (s, 2H), 3.42 (s, 2H), 1.57 (s, 9H). ^13^C NMR (101 MHz, CDCl_3_) *δ* 171.5, 158.2, 152.3, 144.1, 123.4, 85.8, 84.9, 43.2, 27.8. ^1^H NMR is in accordance with the literature [10].

**7,9-Dibromo-8-oxo-1-oxa-2-azaspiro[4.5]deca-2,6,9-triene-3-carboxylic acid (10b)**

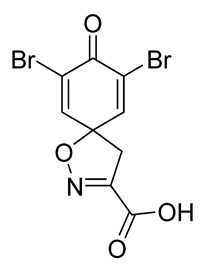


To a solution of *tert*-butyl 7,9-dibromo-8-oxo-1-oxa-2-azaspiro[4.5]deca-2,6,9-triene-3-carboxylate **9b** (0.58 g, 1.4 mmol) in anhydrous DCM (10 mL), trifluoroacetic acid (5.0 mL) was added dropwise. The resulting mixture was stirred at room temperature for 1 h, after which the solvent was removed in vacuo. The product was purified via trituration with Et_2_O to give the compound **10b** as a white solid (0.50 g, 98%). ^1^H NMR (400 MHz, *d*_6_-DMSO) *δ* 7.81 (s, 2H), 3.52 (s, 2H). ^1^H NMR is in accordance with the literature [22]. ^13^C NMR (101 MHz, *d*_6_-acetone) *δ* 172.3, 160.7, 153.6, 146.8, 123.2, 87.3, 43.7. HRMS (ESI-): calculated 303.8609 (C_8_H_4_Br_2_NO_2_), found 303.8609. LC-MS: [M-CO_2_]^−^ *m*/*z* 304 (*t*_R_ = 2.12 min), >99%. Mp: 174–175 °C, (Lit. Mp: 167–168 °C) [10].

The spiro carboxylate core was mainly constructed using the synthetic method given below, followed by *N*-(3-dimethylaminopropyl)-*N*′-ethylcarbodiimide (EDC)-mediated coupling (General procedures for coupling A and B) to give the corresponding product. Some compounds were deprotected with trifluoroacetic acid to give the corresponding trifluoroacetate salts (General procedure C).

**General procedure for EDC-mediated coupling** (**A**)**.** To a stirred solution of carboxylic acid **10a** or **10b** (0.36 mmol) in DCM (5 mL), 1-hydroxybenzotriazole (HOBt) hydrate (0.036 mmol, 0.1 equiv) and *N*-(3-dimethylaminopropyl)-*N*′-ethylcarbodiimide (EDC) hydrochloride (0.39 mmol, 1.1 equiv) at 0–5 °C were added and stirred for 15 min. After this, the amine/hydrazide (0.36 mmol, 1.0 equiv) was added. The reaction mixture was allowed to reach room temperature and was stirred for a further 8–16 h. The mixture was diluted with DCM (10 mL) and washed with 1 M hydrochloric acid (5 mL), a saturated solution of NaHCO_3_ in H_2_O (5 mL), and brine (5 mL). The organic layer was dried over anhydrous Na_2_SO_4_, filtered, and concentrated in vacuo. The crude product was purified by automated flash chromatography (*n*-heptane/EtOAc gradient: 0→100%) to give the pure product.

**General procedure for EDC-mediated coupling** (**B**)**.** Carboxylic acid **10a** or **10b** (0.3 mmol), amine (0.45 mmol, 1.5 equiv), HOBt hydrate (0.45 mmol, 1.5 equiv), and EDC·HCl (0.45 mmol, 1.5 equiv) were dissolved in anhydrous DCM (3 mL). The mixture was irradiated under microwave conditions at 60 °C for 2 h, after which it was diluted with DCM (10 mL). The solution was washed with a saturated solution of NH_4_Cl in H_2_O, water, and brine. The organic phase was dried over anhydrous Na_2_SO_4_, filtered, and concentrated in vacuo. The crude product was purified with automated flash column chromatography (*n*-heptane/EtOAc-EtOH 3:1 (12→100%) to give the pure product.

**General procedure for deprotection of the Boc groups** (**C**)**.** To a solution of clavatadine bis-Boc-derivative (0.28 mmol) in DCM (2 mL), TFA (1 mL) at 0–5 °C was added dropwise. The reaction mixture was allowed to reach room temperature. The resulting mixture was stirred for 3 h at room temperature. The solvent was removed in vacuo to give the crude product, which was triturated in Et_2_O to give a solid trifluoroacetate salt.

**7,9-Dibromo-*N*-(4-guanidinobutyl)-8-oxo-1-oxa-2-azaspiro[4.5]deca-2,6,9-triene-3-carboxamide TFA salt (1-TFA)**

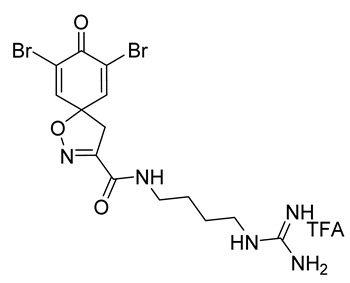


General procedures **A** and **C** were followed to give the clavatadine C TFA salt **12** as an off-white solid (0.10 g, 77%). ^1^H NMR (400 MHz, *d*_6_-DMSO) *δ* 8.65 (t, *J* = 5.9 Hz, 1H), 7.80 (s, 2H), 7.59 (t, *J* = 5.7 Hz, 1H), 3.55 (s, 2H), 3.23–3.06 (m, 4H), 1.48 (m, 4H). ^13^C NMR (101 MHz, *d*_6_-DMSO) *δ* 171.6, 158.2, 156.7, 155.0, 146.7, 121.6, 85.2, 43.2, 40.4, 38.4, 26.0, 25.9. Spectra are in accordance with the literature [10]. HRMS (ESI^+^): Calculated 463.9757 (C_14_H_18_Br_2_N_5_O_3_), found 463.9755. LC-MS: [M + H]^+^ *m*/*z* 464 (*t*_R_ = 2.25 min), >91%. Mp: 115–118 °C, decomp. (Lit. Mp: 130–140 °C, decomp.) [10].

***N*-(4-Guanidinobutyl)-8-oxo-1-oxa-2-azaspiro[4.5]deca-2,6,9-triene-3-carboxamide TFA salt (11)**

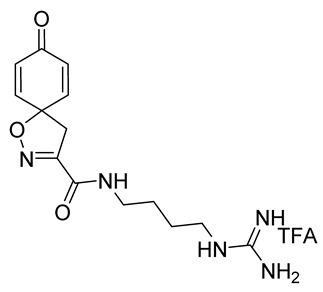


General procedures **A** and **C** were followed to give the compound **11** as an off-white solid (0.11 g, 34%). ^1^H NMR (400 MHz, CD_3_OD) *δ* 7.04–7.00 (m, 2H), 6.26–6.22 (m, 2H), 3.44 (s, 2H), 3.36–3.34 (m, 2H), 3.23–3.20 (m, 2H), 1.64 (m, 4H). ^13^C NMR (101 MHz, CD_3_OD) *δ* 186.4, 161.3, 158.6, 153.3, 146.7, 129.4, 83.6, 44.4, 42.0, 39.8, 27.5, 27.1. HRMS (ESI^+^): calculated 306.1566 (C_14_H_20_N_5_O_3_), found 306.1567. LC-MS: [M + H]^+^ *m*/*z* 306 (*t*_R_ = 0.92 min), >98%. Mp: 170–173 °C.

***N*,*N*-Dimethyl-8-oxo-1-oxa-2-azaspiro[4.5]deca-2,6,9-triene-3-carboxamide (12)**

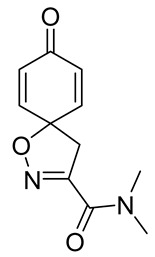


General procedure **A** was followed to give the compound **12** as an off-white amorphous solid (0.011 g, 28%). ^1^H NMR (400 MHz, CD_3_OD) *δ* 7.05 (d, *J* = 10.1 Hz, 2H), 6.25 (d, *J* = 10.1 Hz, 2H), 3.50 (s, 2H), 3.28 (s, 3H), 3.06 (s, 3H). ^13^C NMR (101 MHz, CD_3_OD) *δ* 185.1, 160.7, 153.4, 145.5, 128.7, 80.6, 45.2, 37.6, 34.7. HRMS (ESI^+^): calculated 221.0926 (C_11_H_13_N_2_O_3_), found 221.0927. LC-MS: [M + H]^+^ *m*/*z* 221 (*t*_R_ = 1.85 min), >99%.

**7,9-Dibromo-*N*,*N*-dimethyl-8-oxo-1-oxa-2-azaspiro[4.5]deca-2,6,9-triene-3-carboxamide (13)**

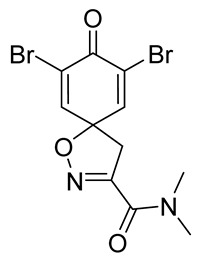


General procedure **A** was followed to give the compound **13** as a white amorphous solid (0.12 g, 37%). ^1^H NMR (400 MHz, CDCl3) *δ* 7.32 (s, 2H), 3.55 (s, 2H), 3.32 (s, 3H), 3.09 (s, 3H). ^13^C NMR (101 MHz, CDCl_3_) *δ* 171.6, 159.3, 153.7, 144.9, 123.8, 84.1, 45.8, 38.8, 36.5. HRMS (ESI^+^): calculated 378.9117 (C_11_H_11_Br_2_N_2_O_3_), found 378.9119.

**7,9-Dibromo-*N*-isopropyl-*N*-methyl-8-oxo-1-oxa-2-azaspiro[4.5]deca-2,6,9-triene-3-carboxamide (14)**

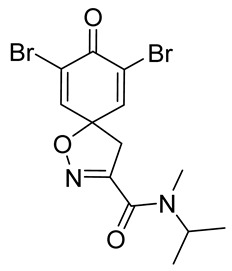


General procedure **A** was followed to give the compound **14** as an off-white amorphous solid (0.061 g, 35%). ^1^H NMR (400 MHz, CDCl_3_) (1:1 mixture of rotamers) *δ* 7.33 (7.32) (s, 2H), 4.84 (4.66) (p, *J* = 6.8 Hz, 1H), 3.56 (3.55) (s, 2H), 3.10 (2.92) (s, 3H), 1.26 (1.19) (d, *J* = 6.8 Hz, 6H). ^13^C NMR (101 MHz, CDCl_3_) *δ* 171.6, 159.0 (158.9), 154.2 (153.5), 144.98 (144.96), 123.71 (123.69), 84.0 (83.9), 49.6 (45.9), 46.03 (45.97), 30.1 (27.0), 20.9 (19.3). HRMS (ESI^+^): calculated 406.9430 (C_13_H_15_Br_2_N_2_O_3_), found 406.9426. LC-MS: [M + H]^+^ *m*/*z* 407 (*t*_R_ = 4.31 min), >99%. Mp: 148–153 °C.

**3-(Morpholine-4-carbonyl)-1-oxa-2-azaspiro[4.5]deca-2,6,9-trien-8-one (15)**

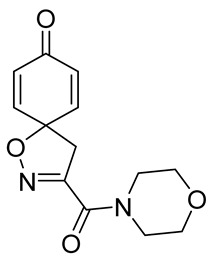


General procedure **A** was followed to give the compound **15** as an off-white amorphous solid (0.012 g, 12%). ^1^H NMR (400 MHz, CDCl_3_) *δ* 6.90–6.81 (m, 2H), 6.33–6.25 (m, 2H), 4.02–3.95 (m, 2H), 3.80–3.70 (m, 6H), 3.48 (s, 2H). ^13^C NMR (101 MHz, CDCl_3_) *δ* 184.5, 158.9, 153.2, 144.1, 129.5, 80.9, 67.1, 66.8, 47.4, 46.2, 43.3. HRMS (ESI^+^): calculated 263.1032 (C_13_H_1_5N_2_O_4_), found 263.1036. LC-MS: [M + H]^+^ *m*/*z* 442 (*t*_R_ = 5.39 min), >99%.

**3-(4-Hydroxypiperidine-1-carbonyl)-1-oxa-2-azaspiro[4.5]deca-2,6,9-trien-8-one (16)**

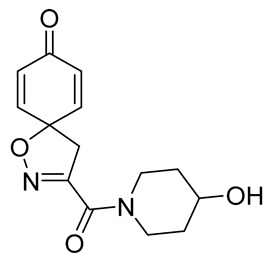


General procedure **A** was followed to give the compound **16** as an off-white amorphous solid (0.024 g, 24%). ^1^H NMR (400 MHz, CDCl_3_): *δ* 6.92–6.83 (m, 2H), 6.33–6.24 (m, 2H), 4.23–4.14 (m, 1H), 4.08–4.00 (m, 2H), 3.72–3.60 (m, 1H), 3.53–3.42 (m, 3H), 2.02–1.90 (m, 2H), 1.69–1.59 (m, 2H), 1.57 (br s, 1H). ^13^C NMR (101 MHz, CDCl_3_) *δ* 184.5, 158.4, 153.2, 144.3, 129.4, 80.8, 66.7, 46.3, 39.9, 34.7, 33.8. HRMS (ESI^+^): calculated 277.1188 (C_14_H_17_N_2_O_4_), found 277.1187. LC-MS: [M + H]^+^ *m*/*z* 277 (*t*_R_ = 1.61 min), >99%.

***N*’-(2-Morpholinoacetyl)-8-oxo-1-oxa-2-azaspiro[4.5]deca-2,6,9-triene-3-carbohydrazide (17)**

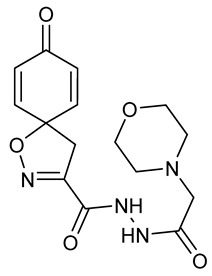


General procedure **A** was followed to give the compound **17** as an amorphous off-white solid (0.030 g, 17%). ^1^H NMR (400 MHz, CD_3_OD) *δ* 7.09–7.00 (m, 2H), 6.29–6.20 (m, 2H), 3.77–3.61 (m, 4H), 3.47 (s, 2H), 3.17 (s, 2H), 2.68–2.55 (m, 4H). ^13^C NMR (101 MHz, CD_3_OD) *δ* 186.4, 171.6, 160.2, 154.3, 146.6, 129.5, 83.5, 67.7, 61.5, 54.7, 44.4. HRMS (ESI^+^): calculated 335.1356 (C_15_H_19_N_4_O_5_), found 335.1354. LC-MS: [M + H]^+^ *m*/*z* 335 (*t*_R_ = 0.53 min), >96%.

***N*-(3,5-Dichlorophenyl)-8-oxo-1-oxa-2-azaspiro[4.5]deca-2,6,9-triene-3-carboxamide (18)**

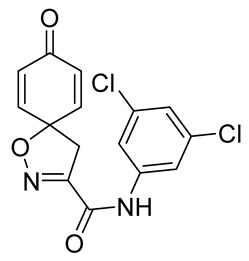


General procedure **A** was followed to give the compound **18** as an off-white amorphous solid (0.098 g, 51%). ^1^H NMR (400 MHz, CDCl_3_) *δ* 8.36 (br s, 1H), 7.56 (d, *J* = 1.8 Hz, 2H), 7.17 (t, *J* = 1.8 Hz, 1H), 6.89–6.84 (m, 2H), 6.35–6.29 (m, 2H), 3.45 (s, 2H). ^13^C NMR (101 MHz, CDCl_3_) *δ* 183.9, 156.3, 153.4, 143.1, 138.2, 135.4, 129.4, 125.0, 117.9, 83.6, 42.7. HRMS (ESI^+^): calculated 337.0147 (C_15_H_11_C_l2_N_2_O_3_), found 337.0148. LC-MS: [M + H]^+^ *m*/*z* 337. (*t*_R_ = 4.81 min), >91%.

**7,9-Dibromo-*N*-(3,5-dichlorophenyl)-8-oxo-1-oxa-2-azaspiro[4.5]deca-2,6,9-triene-3-carboxamide (19)**

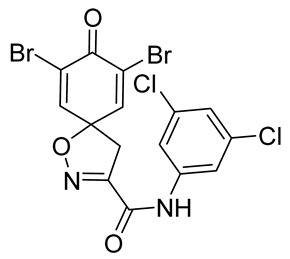


General procedure **B** was followed to give the compound **19** as a white amorphous solid (0.0045 g, 13%). ^1^H NMR (400 MHz, *d*_6_-acetone) *δ* 9.78 (br s, 1H), 7.93 (d, *J* = 1.9 Hz, 2H), 7.77 (s, 2H), 7.26 (t, *J* = 1.8 Hz, 1H), 3.76 (s, 2H). ^13^C NMR (101 MHz, *d*_6_-acetone) *δ* 172.2, 158.3, 155.8, 146.7, 141.3, 135.7, 124.7, 123.3, 119.1, 119.0, 87.4, 43.4. HRMS (ESI^−^): calculated 490.8200 (C_15_H_9_Br_2_Cl_2_N_2_O_3_), found 490.8198. LC-MS: [M-H]^−^ *m*/*z* 491 (*t*_R_ = 5.84 min), >99%.

***N*-(3-Chloro-4-methoxyphenyl)-8-oxo-1-oxa-2-azaspiro[4.5]deca-2,6,9-triene-3-carboxamide (20)**

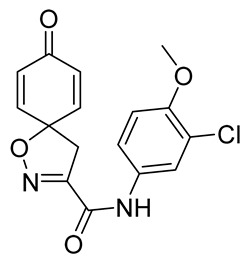


General procedure **A** was followed to give the compound **20** as an off-white amorphous solid (0.025 g, 13%). ^1^H NMR (400 MHz, CDCl_3_) *δ* 8.23 (br s, 1H), 7.68 (d, *J* = 2.6 Hz, 1H), 7.43 (dd, *J* = 8.9, 2.6 Hz, 1H), 6.92 (d, *J* = 8.9 Hz, 1H), 6.90–6.85 (m, 2H), 6.34–6.28 (m, 2H), 3.91 (s, 3H), 3.46 (s, 2H). ^13^C NMR (101 MHz, CDCl_3_) *δ* 184.3, 156.4, 154.0, 152.7, 143.7, 130.2, 129.6, 123.0, 122.5, 119.6, 112.4, 83.5, 56.5, 43.3. HRMS (ESI^+^): calculated 333.0642 (C_16_H_14_ClN_2_O_4_), found 333.0639. LC-MS: [M + H]^+^ *m*/*z* 333 (*t*_R_ = 3.68 min), >99%.

**7,9-Dibromo-*N*-(3-chloro-4-methoxyphenyl)-8-oxo-1-oxa-2-azaspiro[4.5]deca-2,6,9-triene-3-carboxamide (21)**

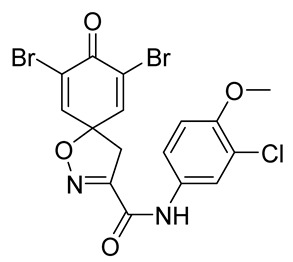


General procedure **B** was followed to give the compound **21** as a white amorphous solid (0.037 g, 18%). ^1^H NMR (400 MHz, *d*_6-_DMSO) *δ* 10.59 (br s, 1H), 7.88 (d, *J* = 2.6 Hz, 1H), 7.85 (s, 2H), 7.68 (dd, *J* = 9.0, 2.6 Hz, 1H), 7.15 (d, *J* = 9.1 Hz, 1H), 3.83 (s, 3H), 3.65 (s, 2H). ^13^C NMR (101 MHz, *d*_6_-DMSO) *δ* 171.6, 156.8, 155.4, 151.2, 146.5, 131.6, 121.8, 121.8, 120.5, 120.2, 112.8, 85.6, 56.2, 43.0. HRMS (ESI^+^): calculated 486.8696 (C_16_H_11_Br_2_ClN_2_O_4_), found 486.8696. LC-MS: [M-H]^−^ *m*/*z* 487 (*t*_R_ = 4.95 min), >94%.

***N*-[2-(1*H*-Indol-3-yl)ethyl]-8-oxo-1-oxa-2-azaspiro[4.5]deca-2,6,9-triene-3-carboxamide (22)**

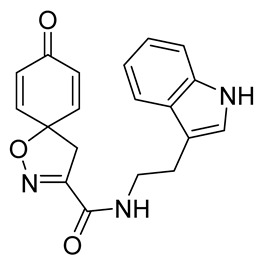


General procedure **A** was followed to give the compound **22** as a white amorphous solid (0.058 g, 56%). ^1^H NMR (400 MHz, CDCl_3_) *δ* 8.12 (br s, 1H), 7.65–7.59 (m, 1H), 7.39 (m, 1H), 7.23 (ddd, *J* = 8.2, 7.2, 1.1 Hz, 1H), 7.15 (ddd, *J* = 8.0, 7.1, 1.0 Hz, 1H), 7.08 (d, *J* = 2.3 Hz, 1H), 6.84–6.78 (m, 2H), 6.67 (br s, 1H), 6.30–6.23 (m, 2H), 3.71 (q, *J* = 6.8 Hz, 2H), 3.36 (s, 2H), 3.06 (t, *J* = 6.8 Hz, 2H). ^13^C NMR (101 MHz, CDCl_3_) *δ* 184.4, 158.7, 153.8, 144.0, 136.5, 129.3, 127.2, 122.5, 122.2, 119.7, 118.8, 112.5, 111.4, 82.7, 43.7, 39.8. HRMS (ESI^+^): calculated 336.1348 (C_19_H_18_N_3_O_3_), found 336.1349. LC-MS: [M-H]^−^ *m*/*z* 336 (*t*_R_ = 4.95 min), >99%.

***N*-(4-Methoxybenzyl)-8-oxo-1-oxa-2-azaspiro[4.5]deca-2,6,9-triene-3-carboxamide (23)**

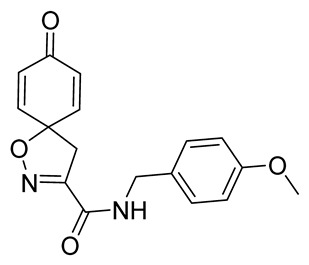


General procedure **A** was followed to give the compound **23** as an off-white amorphous solid (0.13 g, 67%). ^1^H NMR (400 MHz, CDCl_3_) *δ* 7.27–7.22 (m, 2H), 6.91–6.87 (m, 3H), 6.86–6.81 (m, 2H), 6.30–6.24 (m, 2H), 4.48 (d, *J* = 5.9 Hz, 2H), 3.81 (s, 3H), 3.41 (s, 2H). ^13^C NMR (101 MHz, CDCl_3_) *δ* 184.4, 159.5, 158.5, 153.7, 143.9, 129.5, 129.4, 114.5, 114.4, 82.9, 55.4, 43.6, 43.3. HRMS (ESI^+^): calculated 313.1188 (C_17_H_17_N_2_O_4_), found 313.1191. LC-MS: [M + H]^+^ *m*/*z* 313 (*t*_R_ = 3.06 min), >99%.

**7,9-Dibromo-*N*-(4-methoxybenzyl)-8-oxo-1-oxa-2-azaspiro[4.5]deca-2,6,9-triene-3-carboxamide (24)**

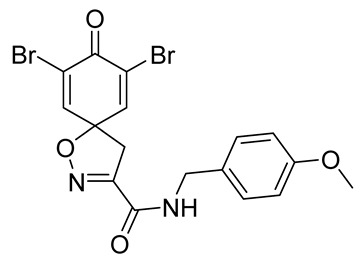


General procedure **A** was followed to give the compound **24** as an off-white amorphous solid (0.022 g, 20%). ^1^H NMR (400 MHz, *d*_6_-DMSO) *δ* 9.07 (t, *J* = 6.2 Hz, 1H), 7.82 (s, 2H), 7.28–7.19 (m, 2H), 6.93–6.84 (m, 2H), 4.29 (d, *J* = 6.2 Hz, 2H), 3.73 (s, 3H), 3.56 (s, 2H). ^13^C NMR (101 MHz, *d*_6_-DMSO) *δ* 171.6, 158.3, 158.1, 155.0, 146.7, 130.8, 128.8, 121.5, 113.6, 85.2, 55.0, 43.1, 41.7. HRMS (ESI^+^Na^+^): calculated 492.9199 (C_17_H_15_Br_2_N_2_O_4_Na), found 492.9197.

***N*-(4-Methoxyphenethyl)-8-oxo-1-oxa-2-azaspiro[4.5]deca-2,6,9-triene-3-carboxamide (25)**

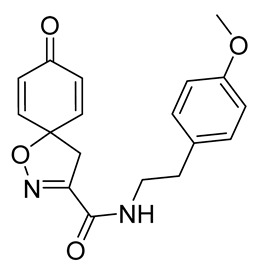


General procedure **A** was followed to give the compound **25** as an off-white amorphous solid (0.042 g, 41%). ^1^H NMR (400 MHz, CDCl_3_) *δ* 7.15–7.11 (m, 2H), 6.89–6.85 (m, 2H), 6.85–6.81 (m, 2H), 6.63 (app. t, 1H), 6.31–6.24 (m, 2H), 3.80 (s, 3H), 3.60 (q, *J* = 7.0 Hz, 2H), 3.38 (s, 2H), 2.83 (t, J = 7.0 Hz, 2H). ^13^C NMR (101 MHz, CDCl_3_) *δ* 184.4, 158.6, 158.6, 153.8, 143.9, 130.2, 129.7, 129.4, 114.3, 82.8, 55.4, 43.6, 41.0, 34.8. HRMS (ESI^+^): calculated 327.1345 (C_18_H_19_N_2_O_4_), found 327.1348. LC-MS: [M + H]^+^ *m*/*z* 327 (*t*_R_ = 3.35 min), >99%.

**8-Oxo-*N*-(pyridin-3-yl)-1-oxa-2-azaspiro[4.5]deca-2,6,9-triene-3-carboxamide (26)**

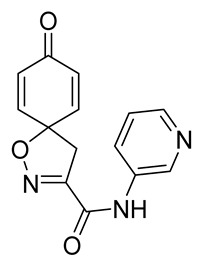


General procedure **A** was followed to give the compound **26** as an off-white amorphous solid (0.047 g, 68%). ^1^H NMR (400 MHz, CDCl_3_) *δ* 8.72 (d, *J* = 2.5 Hz, 1H), 8.44 (dd, *J* = 4.7, 1.4 Hz, 1H), 8.40 (br s, 1H), 8.14 (m, 1H), 7.33 (dd, *J* = 8.3, 4.7 Hz, 1H), 6.91–6.86 (m, 2H), 6.35–6.30 (m, 2H), 3.47 (s, 2H). ^13^C NMR (101 MHz, CDCl_3_) *δ* 184.2, 157.0, 153.8, 146.4, 143.5, 141.5, 133.7, 129.7, 127.2, 124.0, 83.8, 43.1. HRMS (ESI^+^): calculated 270.0879 (C_14_H_12_N_3_O_3_), found 270.0876. LC-MS: [M + H]^+^ *m*/*z* 270 (*t*_R_ = 1.03 min), >92%.

**7,9-Dibromo-8-oxo-*N*-(pyridin-3-yl)-1-oxa-2-azaspiro[4.5]deca-2,6,9-triene-3-carboxamide (27)**

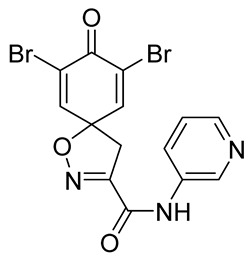


General procedure **B** was followed to give the compound **27** as a white amorphous solid (0.042 g, 34%). ^1^H NMR (400 MHz, *d*_6_-DMSO) *δ* 10.81 (br s, 1H), 8.93 (dd, *J* = 2.6, 0.8 Hz, 1H), 8.33 (dd, *J* = 4.7, 1.5 Hz, 1H), 8.14 (ddd, *J* = 8.4, 2.6, 1.5 Hz, 1H), 7.86 (s, 2H), 7.40 (ddd, *J* = 8.3, 4.7, 0.8 Hz, 1H), 3.66 (s, 2H). ^13^C NMR (101 MHz, *d*_6_-DMSO) *δ* 171.6, 157.4, 155.2, 146.4, 145.1, 142.0, 134.9, 127.4, 123.6, 121.8, 85.8, 42.9. HRMS (ESI^+^): calculated 425.9089 (C_14_H_10_Br_2_N_3_O_3_), found 425.9090. LC-MS: [M + H]^+^ *m*/*z* 426 (*t*_R_ = 2.49 min), >99%.

**8-Oxo-*N*-(pyridin-2-yl)-1-oxa-2-azaspiro[4.5]deca-2,6,9-triene-3-carboxamide (28)**

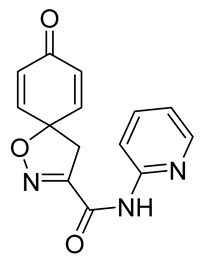


General procedure **A** was followed to give the compound **28** as an off-white amorphous solid (0.040 g, 53%). ^1^H NMR (400 MHz, CDCl_3_) *δ* 9.01 (br s, 1H), 8.35 (ddd, *J* = 5.0, 1.9, 1.0 Hz, 1H), 8.18–8.16 (m, 1H), 7.76 (ddd, *J* = 8.4, 7.4, 1.9 Hz, 1H), 7.12 (ddd, *J* = 7.4, 5.0, 1.0 Hz, 1H), 6.92–6.85 (m, 2H), 6.35–6.28 (m, 2H), 3.46 (s, 2H). ^13^C NMR (101 MHz, CDCl_3_) *δ* 184.3, 156.9, 153.7, 150.2, 148.4, 143.7, 138.7, 129.5, 120.7, 114.2, 83.6, 43.1. HRMS (ESI^+^): calculated 270.0879 (C_14_H_12_N_3_O_3_), found 270.0881. LC-MS: [M + H]^+^ *m*/*z* 270 (*t*_R_ = 2.11 min), >99%.

**7,9-Dibromo-8-oxo-*N*-(pyridin-2-yl)-1-oxa-2-azaspiro[4.5]deca-2,6,9-triene-3-carboxamide (29)**

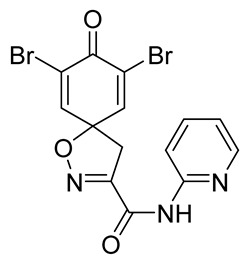


General procedure **B** was followed to give the compound **29** as a white amorphous solid (0.016 g, 13%). ^1^H NMR (400 MHz, *d*_6_-acetone) *δ* 9.22 (br s, 1H), 8.36 (ddd, *J* = 4.9, 1.9, 0.9 Hz, 1H), 8.15 (dt, *J* = 8.3, 1.0 Hz, 1H), 7.85 (ddd, *J* = 8.3, 7.4, 1.9 Hz, 1H), 7.80 (s, 2H), 7.19 (ddd, *J* = 7.4, 4.9, 1.0 Hz, 1H), 3.80 (s, 2H). ^13^C NMR (101 MHz, *d*_6_-acetone) *δ* 172.3, 157.9, 155.9, 151.6, 150.7, 149.4, 146.7, 139.3, 123.3, 121.3, 114.5, 87.5, 43.3. HRMS (ESI^+^): calculated 427.9245 (C_14_H_12_Br_2_N_3_O_3_), found 427.9243. LC-MS: [M + H]^+^ *m*/*z* 426 (*t*_R_ = 3.92 min), >95%.

**7,9-Dibromo-8-oxo-*N*-[5-(trifluoromethyl)pyridin-2-yl]-1-oxa-2-azaspiro[4.5]-deca-2,6,9-triene-3-carboxamide (30)**

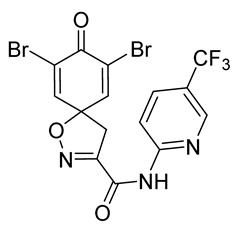


General procedure **A** was followed to give the compound **30** as an off-white amorphous solid (0.012 g, 10%). ^1^H NMR (400 MHz, CDCl_3_) *δ* 9.14 (br s, 1H), 8.62 (d, *J* = 2.3 Hz, 1H), 8.32 (d, *J* = 8.7 Hz, 1H), 7.99 (dd, *J* = 8.7, 2.4 Hz, 1H), 7.34 (s, 2H), 3.56 (s, 2H). ^13^C NMR (101 MHz, CDCl_3_) *δ* 171.3, 156.7, 153.6, 152.6, 146.0, 144.5 (q, *J*_C,F_ = 40.4 Hz), 143.9, 136.2 (q, *J*_C,F_ = 33.3 Hz), 124.3, 123.9, 113.6, 87.0, 42.4. HRMS (ESI^+^): calculated 495.8643 (C_15_H_9_Br_2_F_3_N_3_O_3_), found 495.8941. LC-MS: [M + H]^+^ *m*/*z* 496 and 498 (*t*_R_ = 5.30 and 5.15 min), >97%.

**7,9-Dibromo-8-oxo-*N*-[6-(trifluoromethyl)pyridin-3-yl]-1-oxa-2-azaspiro[4.5] deca-2,6,9-triene-3-carboxamide (31)**

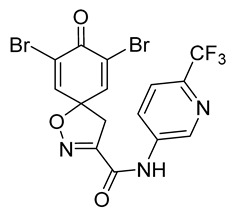


General procedure **A** was followed to give the compound **31** as an off-white amorphous solid (0.015 g, 13%). ^1^H NMR (400 MHz, *d*_6_-acetone) *δ* 10.09 (br s, 1H), 9.14 (d, *J* = 2.4 Hz, 1H), 8.55 (dd, *J* = 8.7, 2.5 Hz, 1H), 7.87 (d, *J* = 8.6 Hz, 1H), 7.78 (s, 2H), 3.80 (s, 2H). ^13^C NMR (101 MHz, *d*_6_-acetone) *δ* 172.3, 158.7 (m), 155.8 (m), 146.7, 143.4 (q, *J*_C,F_ = 34.7 Hz), 142.6 (m), 138.5 (m), 128.4 (m), 123.4, 122.8 (q, *J*_C,F_ = 272.6 Hz), 121.9 (q, *J*_C,F_ = 2.9 Hz), 87.5, 43.4. HRMS (ESI^+^): calculated 495.8943 (C_15_H_9_Br_2_F_3_N_3_O_3_), found 495.8948. LC-MS: [M + H]^+^ *m*/*z* 496 and 498 (*t*_R_ = 4.80 and 4.72 min), >91%.

**8-Oxo-*N*-[2-(pyridin-2-yl)ethyl]-1-oxa-2-azaspiro[4.5]deca-2,6,9-triene-3-carboxamide (32)**

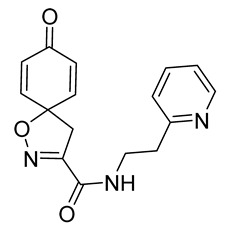


General procedure **A** was followed to give the compound **32** as an off-white amorphous solid (0.076 g, 91%). ^1^H NMR (400 MHz, CDCl_3_) *δ* 8.59–8.54 (m, 1H), 7.71 (app. t, 1H), 7.65 (td, J = 7.7, 1.8 Hz, 1H), 7.21–7.19 (m, 1H), 7.19–7.16 (m, 1H), 6.87–6.81 (m, 2H), 6.29–6.23 (m, 2H), 3.81 (dt, *J* = 6.8, 5.9 Hz, 2H), 3.39 (s, 2H), 3.07 (t, *J* = 6.3 Hz, 2H). ^13^C NMR (101 MHz, CDCl_3_) *δ* 184.5, 159.0, 158.6, 153.9, 149.4, 144.1, 137.0, 129.3, 123.6, 121.9, 82.7, 43.8, 38.6, 36.5. HRMS (ESI^+^): calculated 298.1192 (C_16_H_16_N_3_O_3_), found 298.1191. LC-MS: [M + H]^+^ *m*/*z* 298 (*t*_R_ = 0.74, salt and 1.15 min), >99%.

**7,9-Dibromo-8-oxo-*N*-[2-(pyridin-2-yl)ethyl]-1-oxa-2-azaspiro[4.5]deca-2,6,9-triene-3-carboxamide (33)**

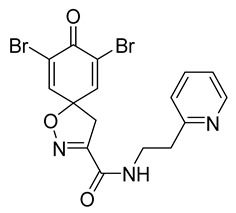


General procedure **B** was followed to give the compound **33** as a white amorphous solid (0.062 g, 24%). ^1^H NMR (400 MHz, CDCl_3_) *δ* 8.59–8.54 (m, 1H), 7.79 (app. t, 1H), 7.64 (td, *J* = 7.7, 1.8 Hz, 1H), 7.30 (s, 2H), 7.21–7.15 (m, 2H), 3.81 (q, *J* = 6.0 Hz, 2H), 3.47 (s, 2H), 3.06 (t, *J* = 6.2 Hz, 2H). ^13^C NMR (101 MHz, CDCl_3_) *δ* 171.5, 159.1, 158.0, 154.1, 149.5, 144.6, 136.9, 123.8, 123.5, 122.0, 85.8, 43.4, 38.7, 36.4. HRMS (ESI^+^): calculated 453.9402 (C_16_H_14_Br_2_N_3_O_3_), found 453.9403. LC-MS: [M + H]^+^ *m*/*z* 454 (*t*_R_ = 2.27 min), >90%.

**7,9-Dibromo-8-oxo-*N*-[2-(pyridin-3-yl)ethyl]-1-oxa-2-azaspiro[4.5]deca-2,6,9-triene-3-carboxamide (34)**

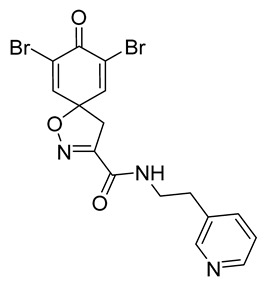


General procedure **A** was followed to give the compound **34** as an off-white solid (0.075 g, 39%). ^1^H NMR (400 MHz, *d*_6_-DMSO) *δ* 8.72 (t, *J* = 5.8 Hz, 1H), 8.44 (dq, *J* = 6.5, 2.6, 1.7 Hz, 2H), 7.80 (s, 2H), 7.66 (dt, *J* = 7.8, 2.0 Hz, 1H), 7.33 (ddd, *J* = 7.8, 4.8, 0.9 Hz, 1H), 3.54 (s, 2H), 3.48–3.38 (m, 2H), 2.83 (t, *J* = 7.2 Hz, 2H). ^13^C NMR (101 MHz, *d*_6_-DMSO) *δ* 171.6, 158.2, 154.9, 149.8, 147.5, 146.7, 146.7, 136.1, 134.6, 123.4, 121.6, 85.2, 43.1, 31.7. HRMS (ESI^+^): calculated 492.9199 (C_16_H_14_Br_2_N_3_O_3_), found 492.9197. Mp: 202–205 °C.

**8-Oxo-*N*-(pyridin-3-ylmethyl)-1-oxa-2-azaspiro[4.5]deca-2,6,9-triene-3-carboxamide (35)**

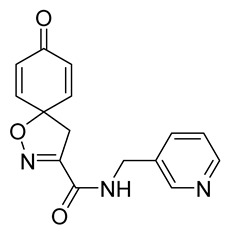


General procedure **B** was followed to give the compound **35** as a white amorphous solid (0.13 g, 61%). ^1^H NMR (400 MHz, *d*_6_-acetone) *δ* 8.59 (dd, *J* = 2.3, 0.9 Hz, 1H), 8.48 (dd, *J* = 4.8, 1.7 Hz, 1H), 8.36 (br s, 1H), 7.80–7.75 (m, 1H), 7.32 (ddd, *J* = 7.9, 4.8, 0.9 Hz, 1H), 7.14–7.05 (m, 2H), 6.25–6.15 (m, 2H), 4.55 (d, *J* = 6.3 Hz, 2H), 3.50 (s, 2H). ^13^C NMR (101 MHz, *d*_6_-acetone) *δ* 184.9, 159.9, 155.1, 150.3, 149.4, 145.9, 136.1, 135.4, 129.2, 124.2, 83.2, 44.2, 41.2. HRMS (ESI^+^): calculated 284.1035 (C_15_H_14_N_3_O_3_), found 284.1032. LC-MS: [M + H]^+^ *m*/*z* 284 (*t*_R_ = 0.68 min), >99%.

**7,9-Dibromo-8-oxo-*N*-(pyridin-3-ylmethyl)-1-oxa-2-azaspiro[4.5]deca-2,6,9-triene-3-carboxamide (36)**

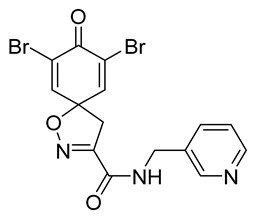


General procedure **B** was followed to give **36** as a white amorphous solid (0.068 g, 55%). ^1^H NMR (400 MHz, *d*_6_-DMSO) *δ* 8.53 (dd, *J* = 2.3, 0.9 Hz, 1H), 8.47 (dd, *J* = 4.8, 1.7 Hz, 1H), 7.83 (s, 2H), 7.74–7.68 (m, 1H), 7.36 (ddd, *J* = 7.8, 4.8, 0.9 Hz, 1H), 4.39 (s, 2H), 3.57 (s, 2H). ^13^C NMR (101 MHz, *d*_6_-DMSO) *δ* 171.6, 158.4, 158.4, 154.9, 154.8, 148.9, 148.2, 146.7, 135.3, 134.3, 134.3, 123.4, 121.6, 85.4, 43.0, 40.0. HRMS (ESI^+^): calculated 439.9245 (C_15_H_12_Br_2_N_3_O_3_), found 439.9244. LC-MS: [M + H]^+^ *m*/*z* 440 (*t*_R_ = 2.08 min), >94%.

**8-Oxo-*N*’-picolinoyl-1-oxa-2-azaspiro[4.5]deca-2,6,9-triene-3-carbohydrazide (37)**

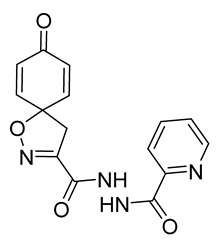


General procedure **A** was followed to give the compound **37** as an off-white solid (0.025 g, 16%). ^1^H NMR (400 MHz, *d*_6_-DMSO) *δ* 10.71 (br s, 1H), 10.65 (br s, 1H), 8.75–8.67 (m, 1H), 8.10–8.00 (m, 2H), 7.69–7.63 (m, 1H), 7.22–7.13 (m, 2H), 6.31–6.22 (m, 2H), 3.54 (s, 2H). ^13^C NMR (101 MHz, *d*_6_-DMSO) *δ* 184.5, 163.0, 157.8, 153.2, 149.1, 148.7, 145.6, 137.9, 128.2, 127.1, 122.5, 81.6, 43.3. HRMS (ESI^+^): calculated 313.0937 (C_15_H_13_N_4_O_4_), found 313.0937. LC-MS: [M + H]+ *m*/*z* 313 (*t*_R_ = 1.51 min), >93%. Mp: 191–193 °C.

**7,9-Dibromo-8-oxo-*N*’-picolinoyl-1-oxa-2-azaspiro[4.5]deca-2,6,9-triene-3-carbohydrazide (38)**

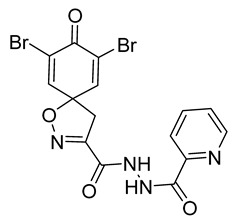


General procedure **A** was followed to give the compound **38** as an off-white solid (0.1 g, 50%). ^1^H NMR (400 MHz, *d*_6-_DMSO) *δ* 10.74 (s, 1H), 10.65 (s, 1H), 8.70 (dt, *J* = 4.7, 1.4 Hz, 1H), 8.08–7.99 (m, 2H), 7.88 (s, 2H), 7.71–7.63 (m, 1H), 3.63 (s, 2H). ^13^C NMR (101 MHz, *d*_6_-DMSO) *δ* 171.7, 163.0, 157.5, 153.6, 149.0, 148.6, 146.7, 137.9, 127.1, 122.5, 121.7, 85.2, 43.5. HRMS (ESI^+^): calculated 470.9128 (C_15_H_11_Br_2_N_4_O_4_), found 470.9129. LC-MS: [M + H]^+^ *m*/*z* 471 and 473 (*t*_R_ = 3.14 and 3.11 min), >98%. Mp: 199–202 °C.

**7,9-Dibromo-8-oxo-*N*’-picolinoyl-1-oxa-2-azaspiro[4.5]deca-2,6,9-triene-3-carbohydrazide (39)**

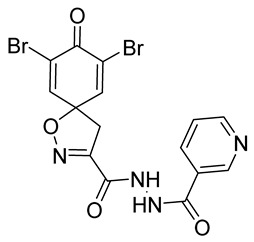


General procedure **A** was followed to give the compound **39** as an off-white solid (0.025 g, 14%). ^1^H NMR (400 MHz, *d*_6_-DMSO) *δ* 10.73 (br s, 2H), 9.06–9.01 (m, 1H), 8.78 (dd, *J* = 4.9, 1.7 Hz, 1H), 8.23 (ddd, *J* = 8.0, 2.3, 1.7 Hz, 1H), 7.88 (s, 2H), 7.62–7.53 (m, 1H), 3.64 (s, 2H). ^13^C NMR (101 MHz, *d*_6_-DMSO) *δ* 171.6, 164.1, 157.7, 153.5, 152.6, 148.4, 146.6, 135.2, 128.0, 123.7, 121.77, 85.2, 43.0. HRMS (ESI^+^): calculated 470.9128 (C_15_H_11_Br_2_N_4_O_4_), found 470.9127. LC-MS: [M + H]^+^ *m*/*z* 471 and 473 (*t*_R_ = 2.48 and 2.37 min), >99%. Mp: 163–166 °C.

***N*-[3-(4-Chlorophenyl)-1-methyl-1*H*-pyrazol-5-yl]-8-oxo-1-oxa-2-azaspiro[4.5]deca-2,6,9-triene-3-carboxamide (40)**

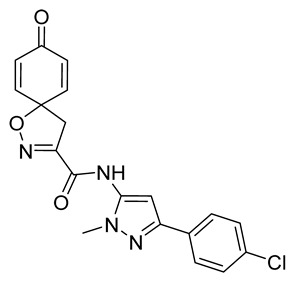


General procedure **A** was followed to give the compound **40** as an off-white solid (0.021 g, 18%). ^1^H NMR (400 MHz, CDCl_3_) *δ* 8.25 (br s, 1H), 7.74–7.68 (m, 2H), 7.40–7.33 (m, 2H), 6.91–6.87 (m, 2H), 6.67 (s, 1H), 6.36–6.31 (m, 2H), 3.86 (s, 3H), 3.48 (s, 2H). ^13^C NMR (101 MHz, CDCl_3_) *δ* 184.0, 156.2, 152.9, 149.2, 143.1, 134.9, 133.7, 131.5, 129.6, 128.8, 126.6, 96.9, 83.8, 42.8, 35.8. HRMS (ESI^+^): calculated 383.0911 (C_19_H_16_ClN_4_O_3_), found 383.0911. LC-MS: [M + H]^+^ *m*/*z* 383 and 385 (*t*_R_ = 4.21 and 4.14min), >99%.

**7,9-Dibromo-*N*-[3-(4-chlorophenyl)-1-methyl-1*H*-pyrazol-5-yl]-8-oxo-1-oxa-2-azaspiro[4.5]deca-2,6,9-triene-3-carboxamide (41)**

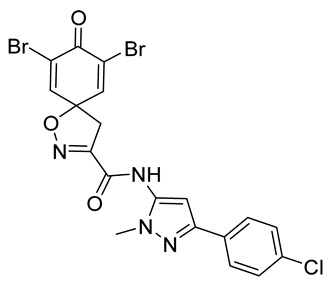


General procedure **A** was followed to give the compound **41** as an off-white solid (0.075 g, 35%). ^1^H NMR (400 MHz, *d*_6_-DMSO) *δ* 10.74 (br s, 1H), 7.88 (s, 2H), 7.85–7.76 (m, 2H), 7.50–7.42 (m, 2H), 3.74 (s, 3H), 3.65 (s, 2H). ^13^C NMR (101 MHz, *d*_6_-DMSO) *δ* 171.6, 157.6, 154.6, 147.1, 146.4, 136.4, 132.0, 132.0, 128.7, 126.5, 121.9, 98.3, 85.9, 42.8, 36.0. Mp: 251–254 °C. HRMS (ESI^+^): calculated 538.9121 (C_19_H_14_Br_2_ClN_4_O_3_), found 538.9120. LC-MS: [M + H]^+^ *m*/*z* 541 (*t*_R_ = 5.29 min), >99%.

**7,9-Dibromo-8-oxo-*N*-(4,5,6,7-tetrahydrobenzo[d]thiazol-2-yl)-1-oxa-2-azaspiro[4.5]deca-2,6,9-triene-3-carboxamide (42)**

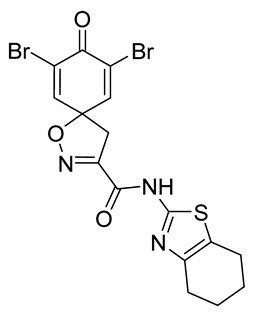


General procedure **A** was followed to give the compound **42** as a pale yellow solid (0.085 g, 40%). ^1^H NMR (400 MHz, *d*_6_-acetone) *δ* 10.92 (br s, 1H), 7.78 (s, 2H), 3.79 (s, 2H), 2.72–2.69 (m, 2H), 2.62–2.59 (m, 2H), 1.84 (p, *J* = 3.2 Hz, 4H). ^13^C NMR (101 MHz, *d*_6_-acetone) *δ* 172.29, 157.75, 155.14, 155.10, 146.75, 145.21, 123.69, 123.34, 87.35, 43.40, 29.84, 26.92, 24.02, 23.67, 23.33. HRMS (ESI^+^): calculated 487.9102 (C_16_H_14_Br_2_N_3_O_3_S), found 487.9109. LC-MS: [M + H]^+^ *m*/*z* 488 and 490 (*t*_R_ = 4.92 and 5.03 min), >99%. Mp: 185–190 °C.

## 5. Conclusions

In summary, the highest cytotoxicity against the A-375 cell line was observed in 2,4-dichloro compound **18** (CC_50_ 0.4 ± 0.3 µM, SI 2) and the highest SI (2.4) was observed for the pyridin-2-yl derivative **29** and hydrazide analog of 2-picoline **37**. The results of these spirocyclic clavatadine analogs provide a path for further mechanistic studies and optimization of simplified spirocyclic bromotyrosine derivatives to understand the elements of their SARs and improve the selectivity.

## Figures and Tables

**Table 1:** Structures of the target spirocyclic bromotyrosine analogs **1-TFA** and **11**–**42**.

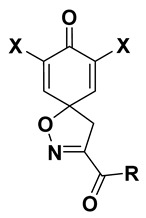											
No	X	-R	No	X	-R	No	X	-R	No	X	-R
**1-TFA**	Br	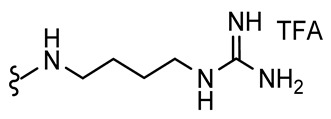	**19**	Br	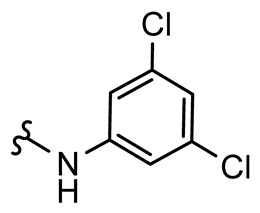	**28**	H	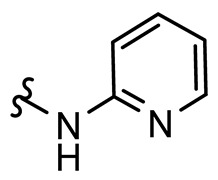	**37**	H	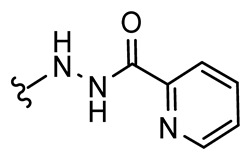
**11**	H	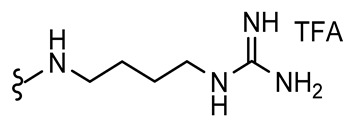	**20**	H	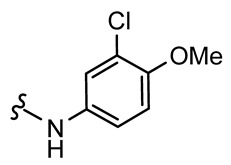	**29**	Br	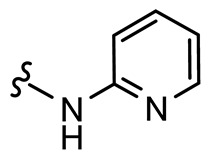	**38**	Br	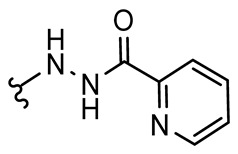
**12**	H	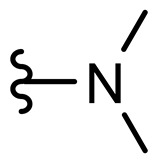	**21**	Br	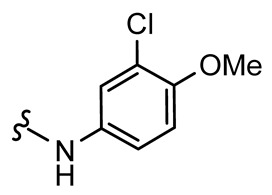	**30**	Br	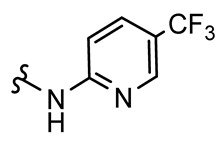	**39**	Br	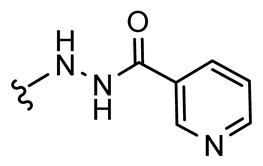
**13**	Br	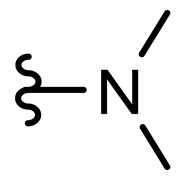	**22**	H	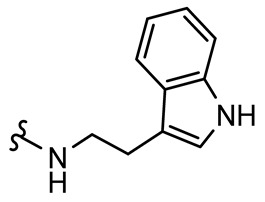	**31**	Br	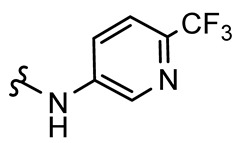	**40**	H	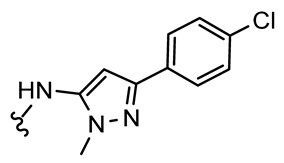
**14**	Br	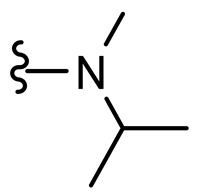	**23**	H	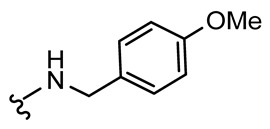	**32**	H	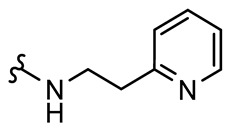	**41**	Br	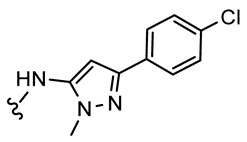
**15**	H	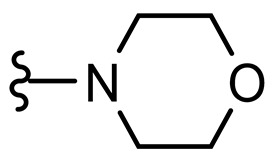	**24**	Br	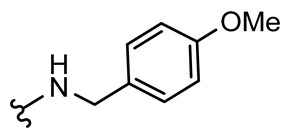	**33**	Br	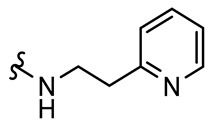	**42**	Br	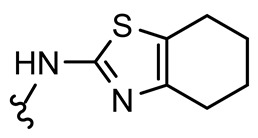
**16**	H	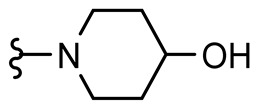	**25**	H	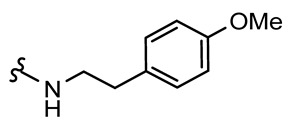	**34**	Br	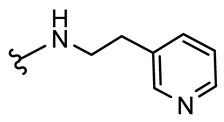			
**17**	H	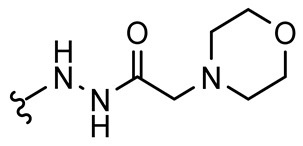	**26**	H	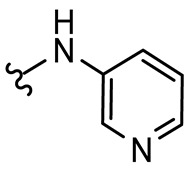	**35**	H	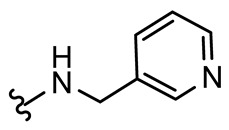			
**18**	H	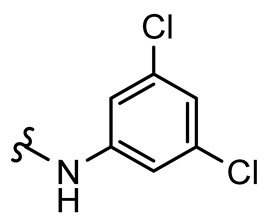	**27**	Br	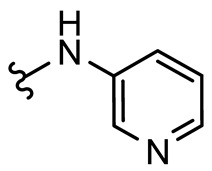	**36**	Br	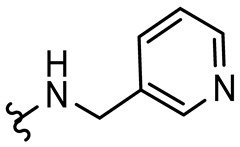			

**Table 2:** Cytotoxicity of compounds **1-TFA** and **11**–**42** against human malignant melanoma cell line A-375 and normal human fibroblast Hs27 cells. Camptothecin, a compound with high selectivity to cancer cells, was used as a positive control. Purpurealidin I **3** was used as a reference compound [14]. CC_50_ = cytotoxic concentration that caused death of 50% of cells. ND = not determined. The primary screening was performed as a single experiment with three technical replicates per sample. The CC_50_ values are arithmetic means from 2–3 independent experiments performed in triplicate. The values in parentheses are standard deviations.

Compound	Primary Test Results (Cytotoxicity % at 50 µM in A-375 Cells)	CC_50_ (µM) in A-375 Cells	CC_50_ (µM) in Hs27 Cells	Selectivity Index (SI)
**1-TFA**	0.5	ND	ND	ND
**11**	−8.2	ND	ND	ND
**12**	95.1	2.1 (1.2)	3.8 (0.7)	1.8 (0.6)
**13**	99.8	3.7 (1.9)	7.2 (6.2)	1.9 (1.0)
**14**	99.9	7.2 (3.2)	7.8 (1.8)	1.1 (0.5)
**15**	96.1	3.8 (0.7)	2.5 (0.3)	0.7 (0.2)
**16**	97.1	4.9 (1.0)	7.4 (0.5)	1.5 (0.2)
**17**	20.8	ND	ND	ND
**18**	100.0	0.4 (0.3)	0.8 (0.2)	2.0 (0.8)
**19**	94.9	9.2 (2.9)	6.8 (2.8)	0.7 (0.5)
**20**	100.0	1.2 (0.1)	1.5 (0.7)	1.3 (0.5)
**21**	101.1	2.3 (0.4)	5.0 (1.1)	2.2 (0.3)
**22**	99.8	0.7 (0.1)	0.7 (0.2)	1.0 (0.3)
**23**	100.0	1.2 (0.2)	1.4 (0.8)	1.2 (0.6)
**24**	97.8	3.5 (1.1)	3.3 (1.1)	0.9 (0.3)
**25**	100.0	1.2 (0.1)	2.0 (0.6)	1.7 (0.3)
**26**	99.2	2.0 (1.2)	1.9 (0.9)	1.0 (0.8)
**27**	100.7	3.0 (0.3)	6.6 (3.1)	2.2 (0.5)
**28**	99.6	1.9 (0.2)	1.5 (0.6)	0.8 (0.4)
**29**	100.6	2.4 (0.3)	5.8 (2.5)	2.4 (0.4)
**30**	98.1	3.6 (0.1)	2.3 (0.4)	0.6 (0.2)
**31**	98.6	5.3 (0.7)	2.1 (0.1)	0.4 (0.1)
**32**	97.5	1.5 (1.0)	2.2 (1.3)	1.5 (0.9)
**33**	98.9	3.6 (1.9)	6.6 (1.3)	1.9 (0.6)
**34**	97.0	5.1 (2.8)	8.8 (1.8)	1.7 (0.6)
**35**	97.5	1.1 (0.1)	2.5 (0.2)	2.3 (0.1)
**36**	100.7	3.4 (0.2)	6.1 (0.9)	1.8 (0.2)
**37**	89.3	2.5 (0.6)	6.0 (1.3)	2.4 (0.3)
**38**	93.5	12.3 (1.3)	6.4 (3.0)	0.5 (0.5)
**39**	75.2	ND	ND	ND
**40**	99.9	0.5 (0.1)	0.5 (0.4)	1.0 (0.8)
**41**	97.6	1.3 (0.4)	1.4 (1.0)	1.1 (0.8)
**42**	97.5	3.5 (1.2)	4.4 (1.4)	1.3 (0.5)
Purpurealidin I **3**	98.9	3.7 (0.5)	4.7 (0.2)	1.3 (0.1)
Camptothecin	94.7	0.05 (0.02)	3.6 (1.0)	70.9 (0.5)

## Data Availability

Data is contained within the article and Supplementary Material.

## Supplementary Materials

The following are available online at https://www.mdpi.com/article/10.3390/md19070400/s1. Experimental data: ^1^H and ^13^C spectra of all compounds.
