# Supplementary material for: Synthesis and Cytotoxicity Evaluation of Spirocyclic Bromotyrosine Clavatadine C Analogs
Source: Mar Drugs. 2021 Jul 20;19(7):400. doi: 10.3390/md19070400 (PMC8305101; doi:10.3390/md19070400)

Supporting Information

Synthesis and Cytotoxicity Evaluation of Spirocyclic Bromotyrosine Clavatadine C Analogs

Piyush A. Patel,[1] Tanja Bruun,[1] Polina Ilina,[2] Heidi Mäkkylä,[2] Antti Lempinen,[1] Jari Yli-Kauhaluoma,[1] Päivi Tammela,[2] Paula S. Kiuru[1*]

[1]Drug Research Program, Division of Pharmaceutical Chemistry and Technology, Faculty of Pharmacy, University of Helsinki, FI-00014 Helsinki, Finland, paula.kiuru@helsinki.fi

[2]Drug Research Program, Division of Pharmaceutical Biosciences, Faculty of Pharmacy, University of Helsinki, FI-00014 Finland

1. ^{1}H and ^{13}C NMR spectra of the compounds S2

1. NMR spectra

^{1}H NMR of clavatidine C TFA-salt, compound **1-TFA** in d_6-DMSO (3.31 ppm)

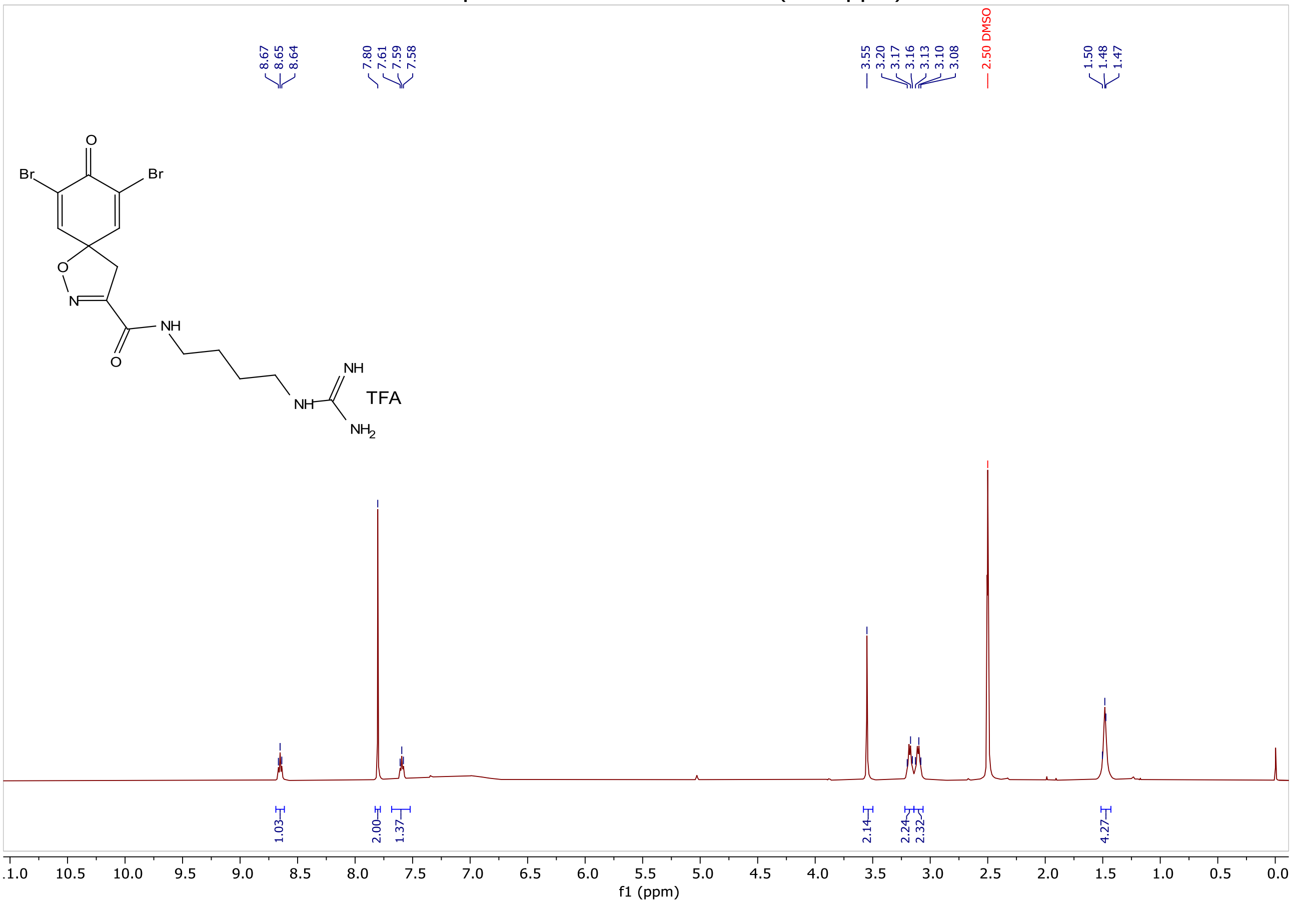

^{13}C NMR of clavatidine C TFA-salt, compound **1-TFA** in d_6-DMSO (39.52 ppm)

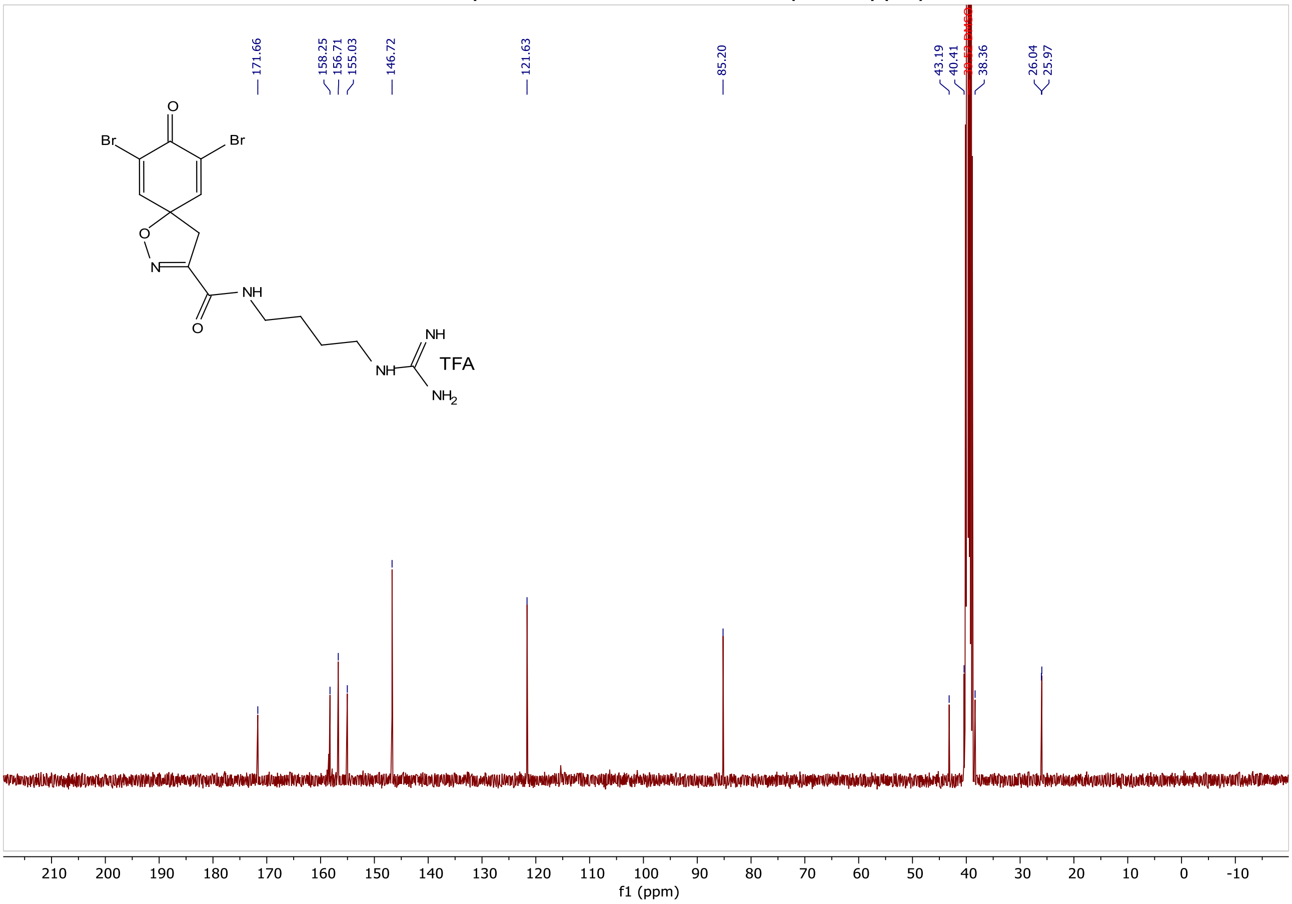

^{1}H NMR of compound **11** in CD_3OD (3.31 ppm)

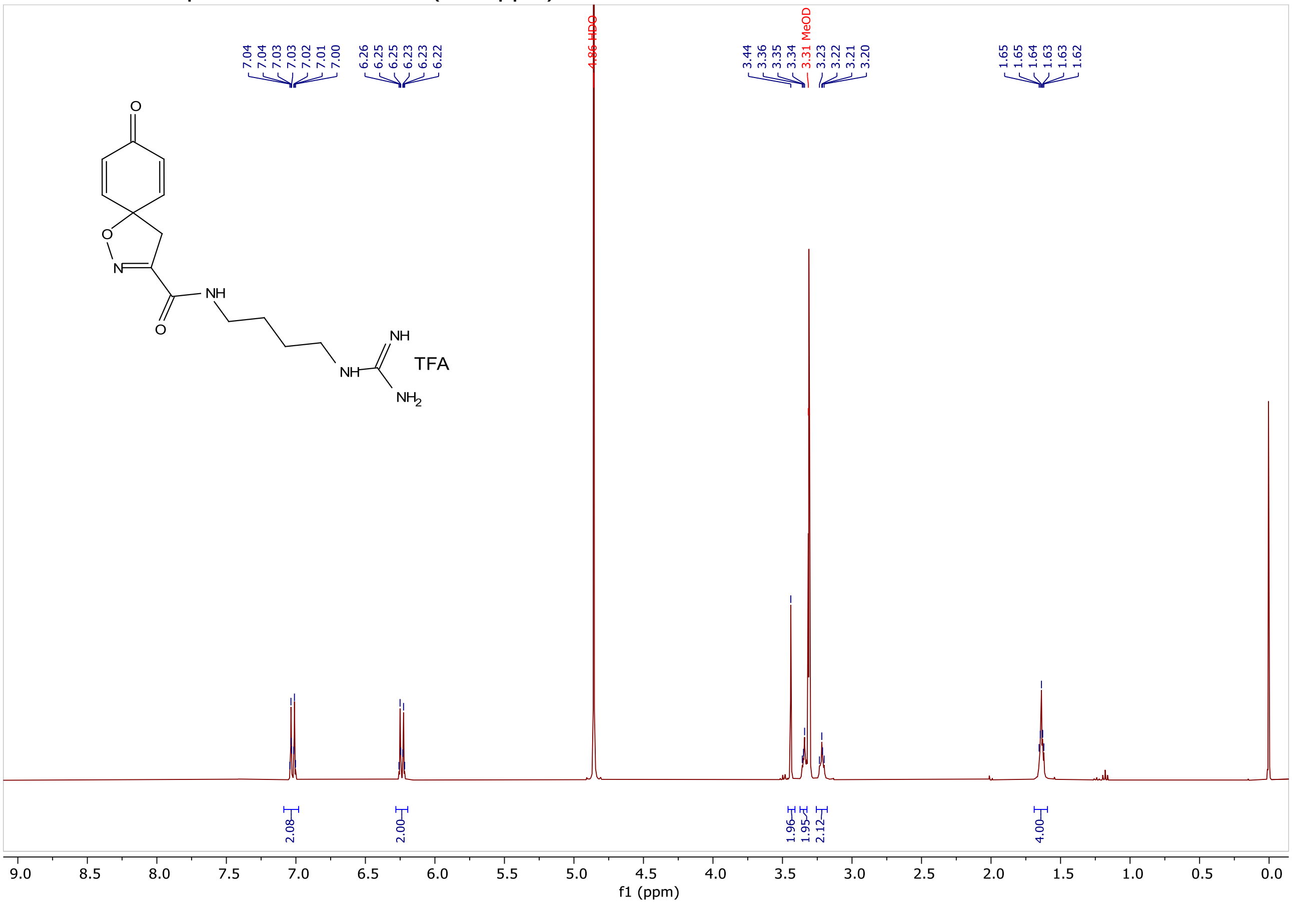

^{13}C NMR of compound **11** in CD_3OD (49.00 ppm)

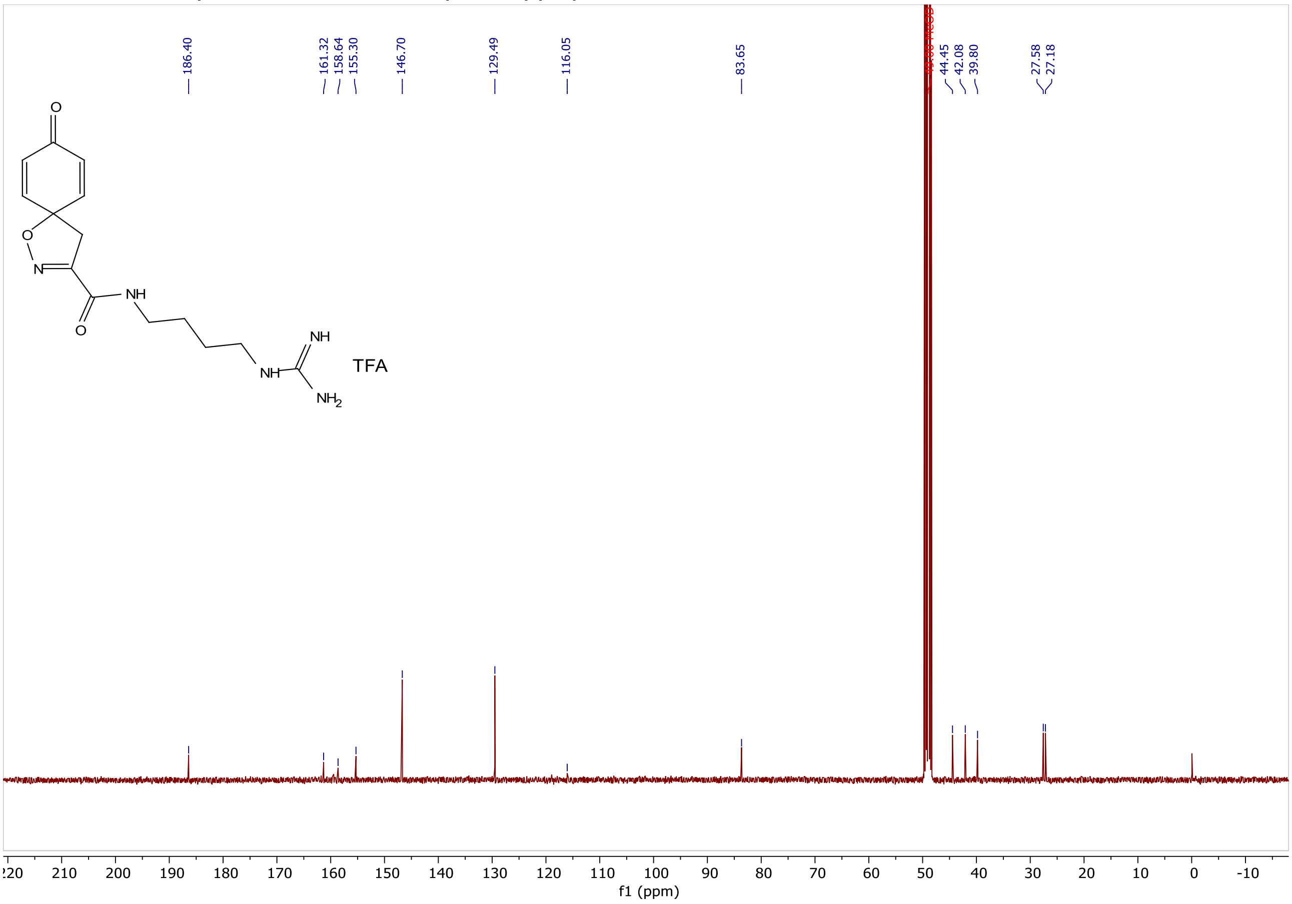

^{1}H NMR of compound **12** in CD_3OD (3.31 ppm)

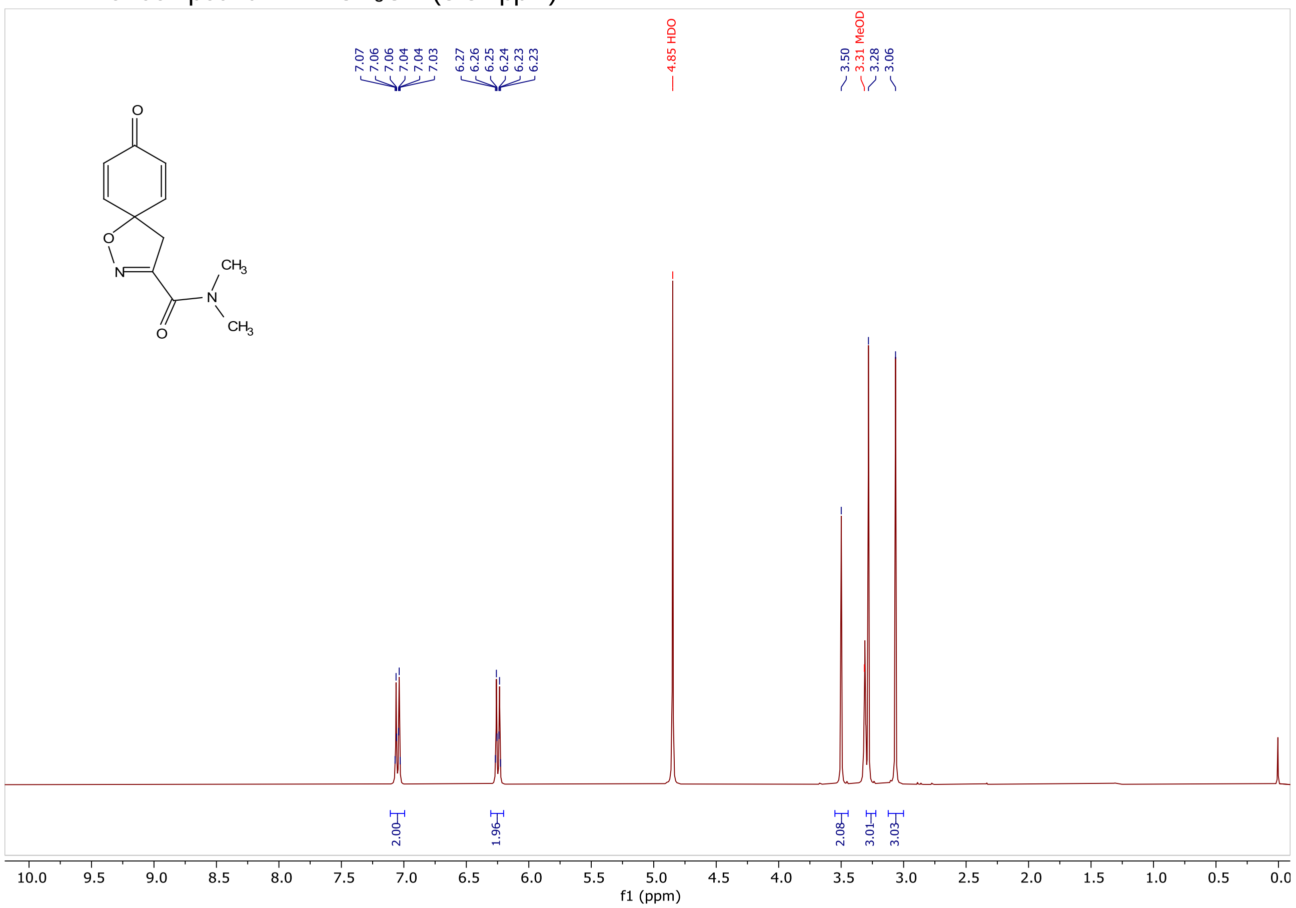

^{13}C NMR of compound **12** in CD_3OD (49.00 ppm)

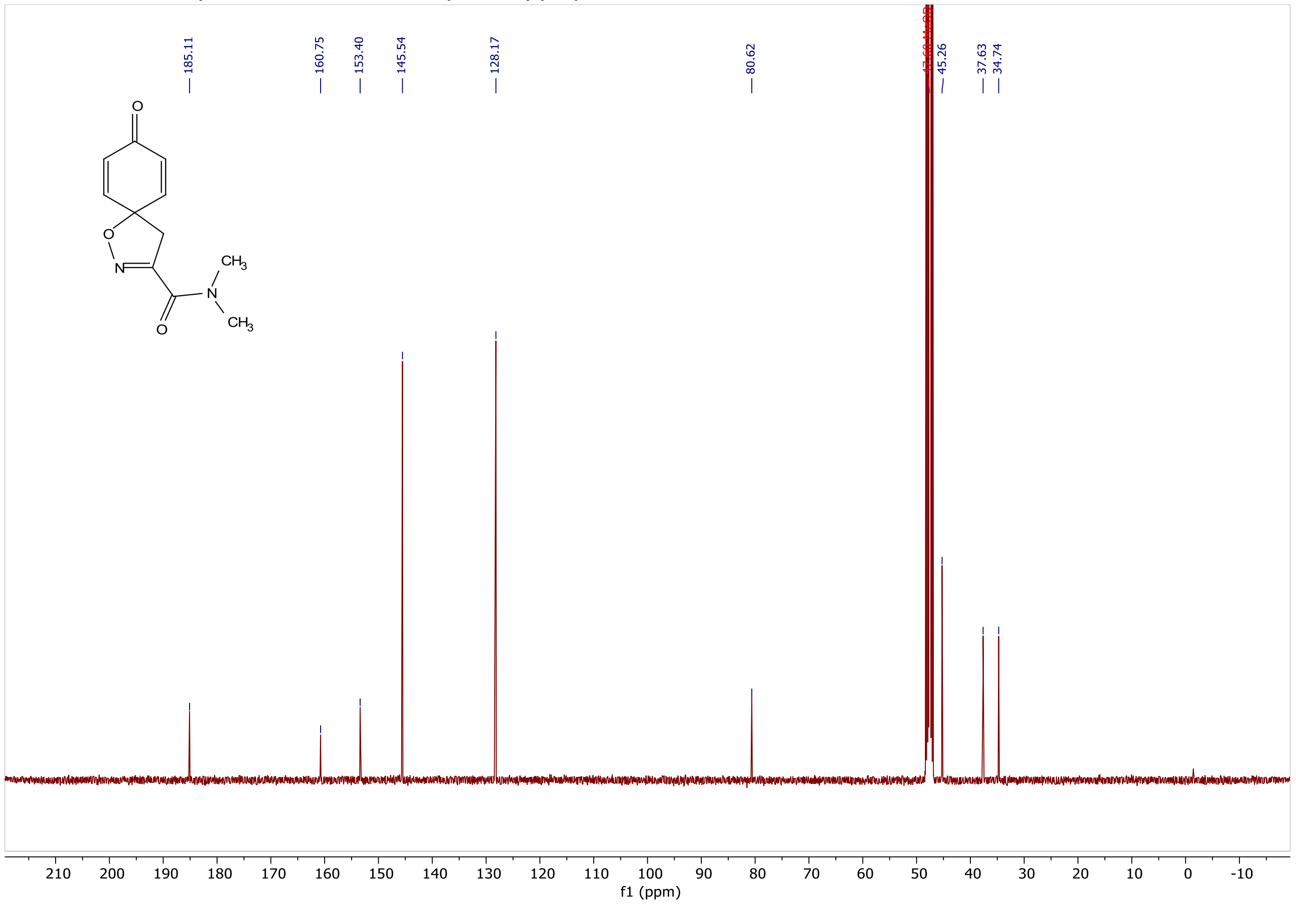

^{1}H NMR of compound **13** in $CDCl_3$ (7.26 ppm)

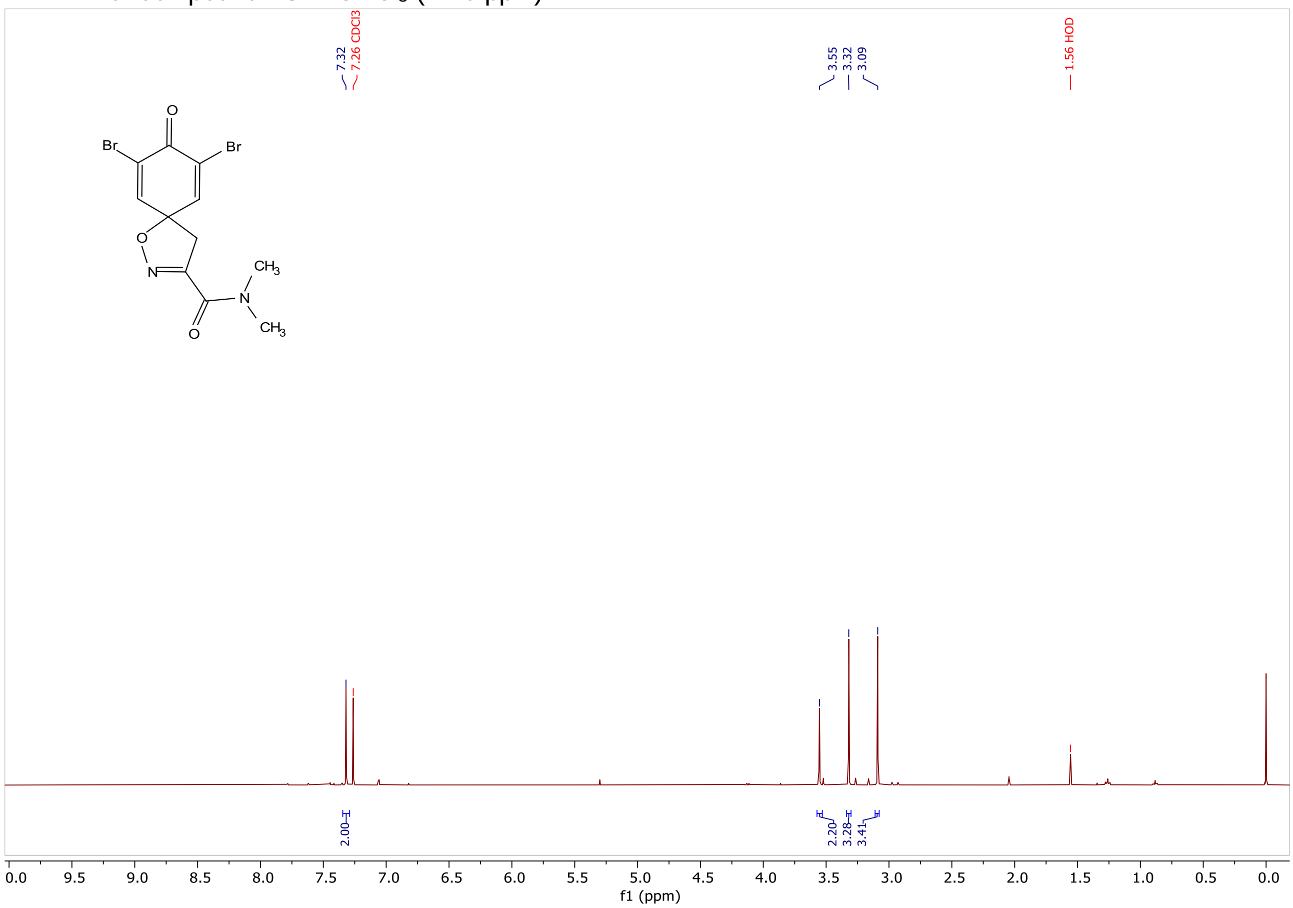

^{13}C NMR of compound **13** in $CDCl_3$ (77.16 ppm)

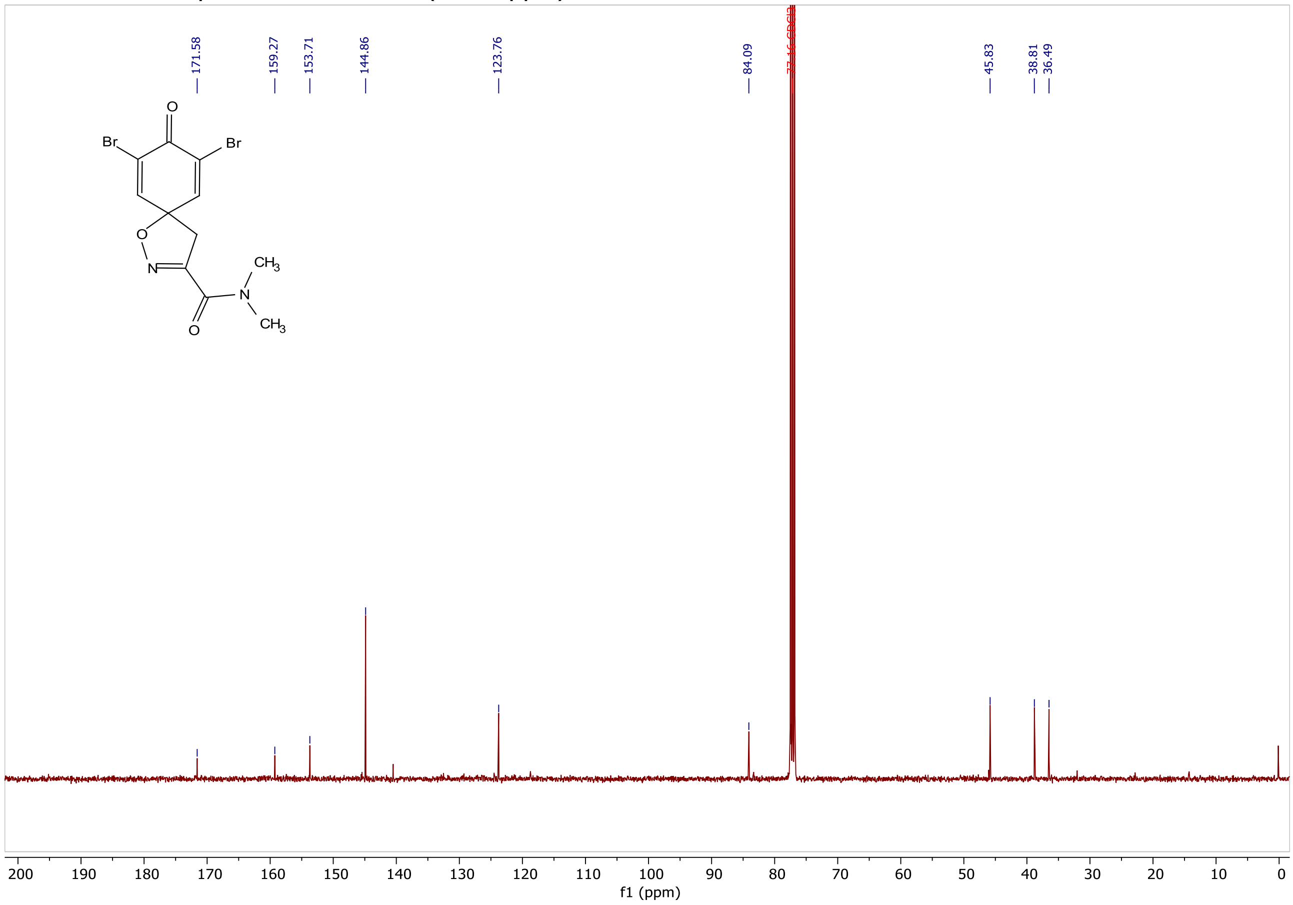

^{1}H NMR of compound **14** in $CDCl_3$ (7.26 ppm)

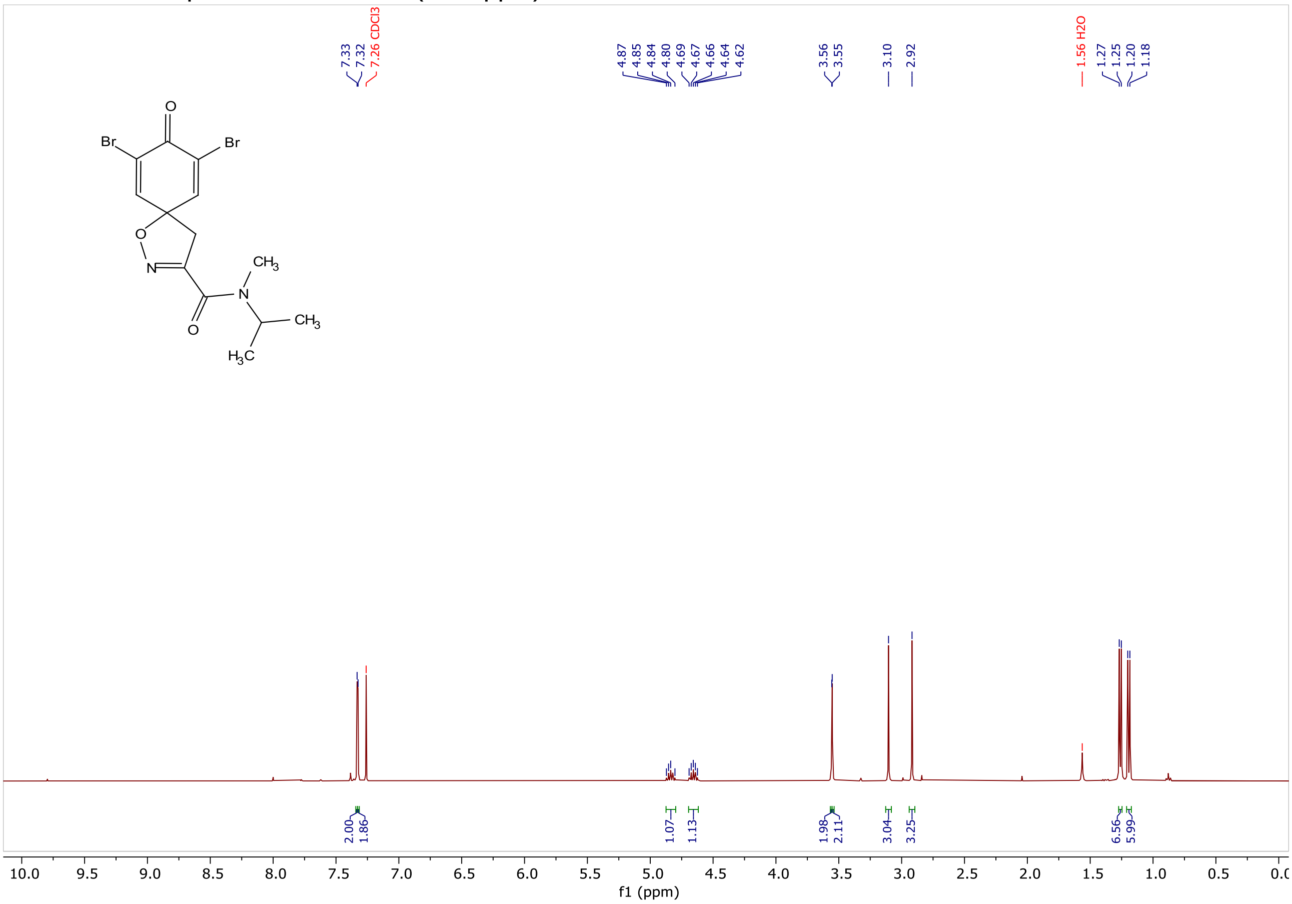

^{13}C NMR of compound **14** in $CDCl_3$ (77.16 ppm)

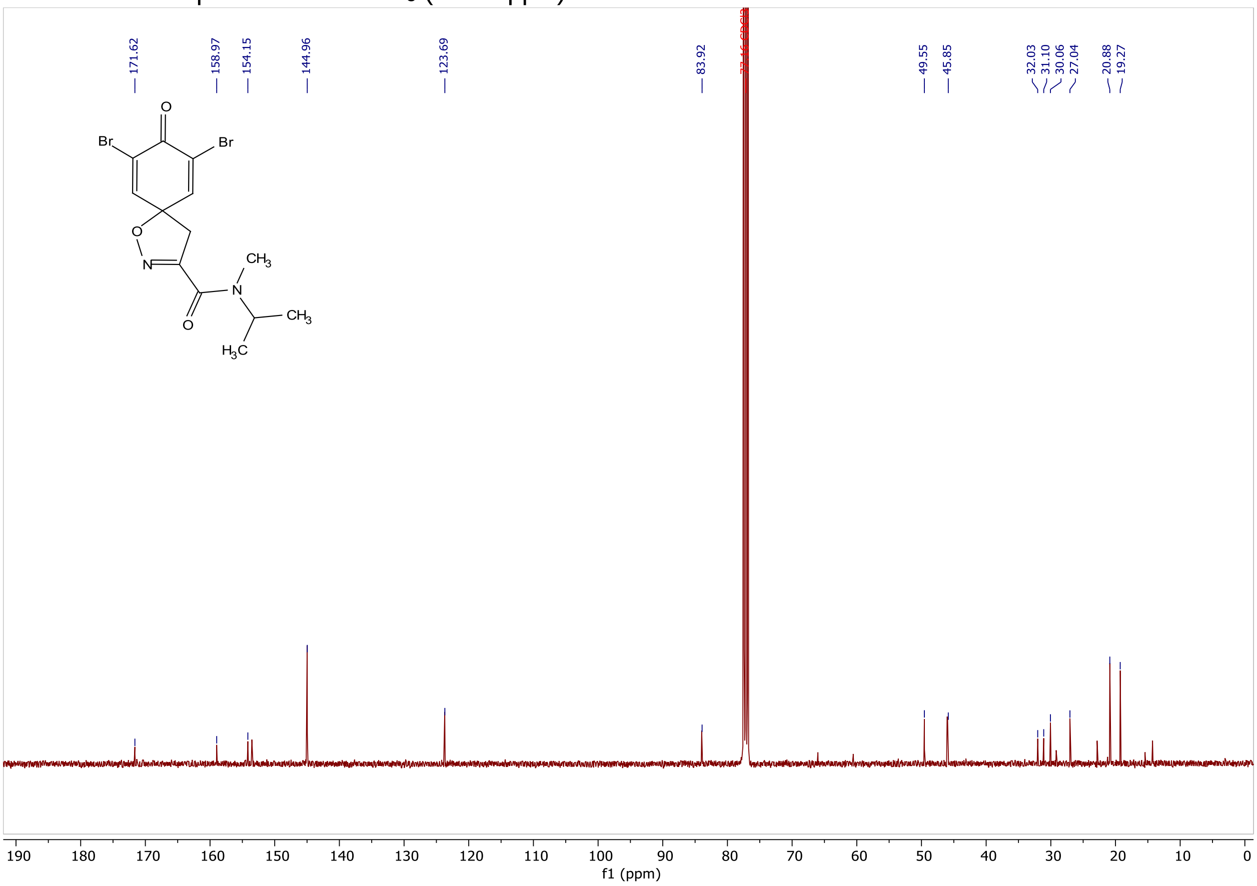

^{1}H NMR of compound **15** in $CDCl_3$ (7.26 ppm)

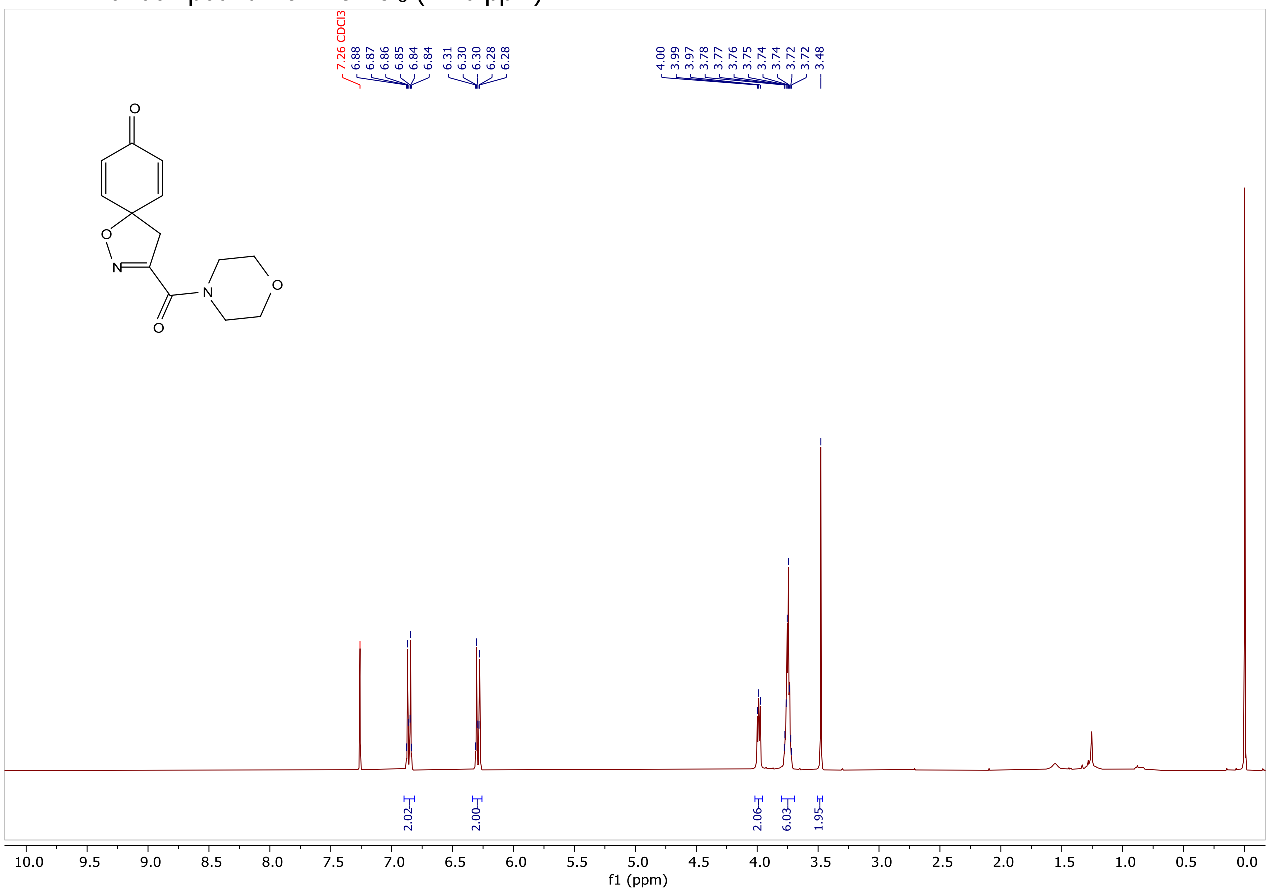

^{13}C NMR of compound **15** in $CDCl_3$ (77.16 ppm)

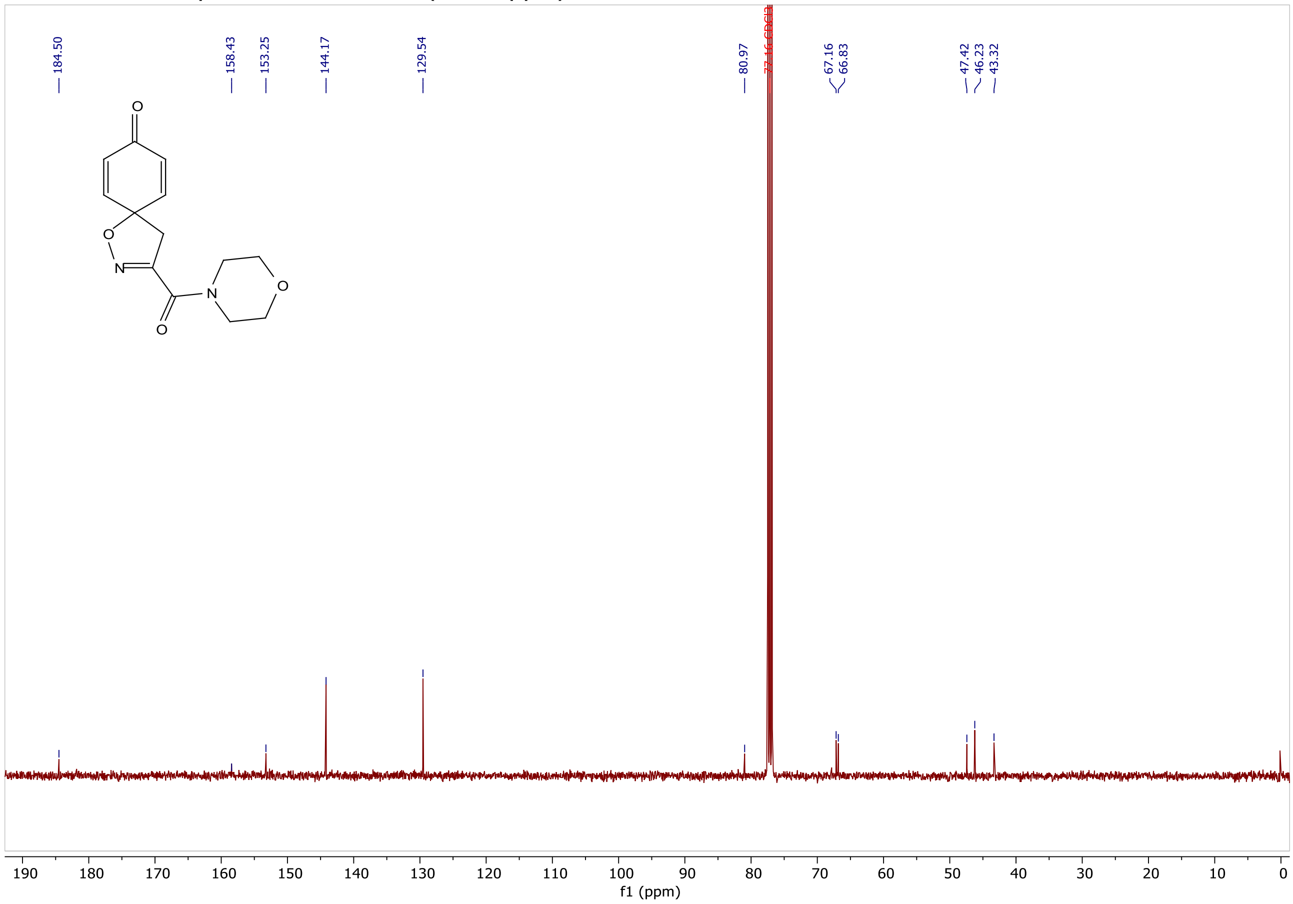

^{1}H NMR of compound **16** in $CDCl_3$ (7.26 ppm)

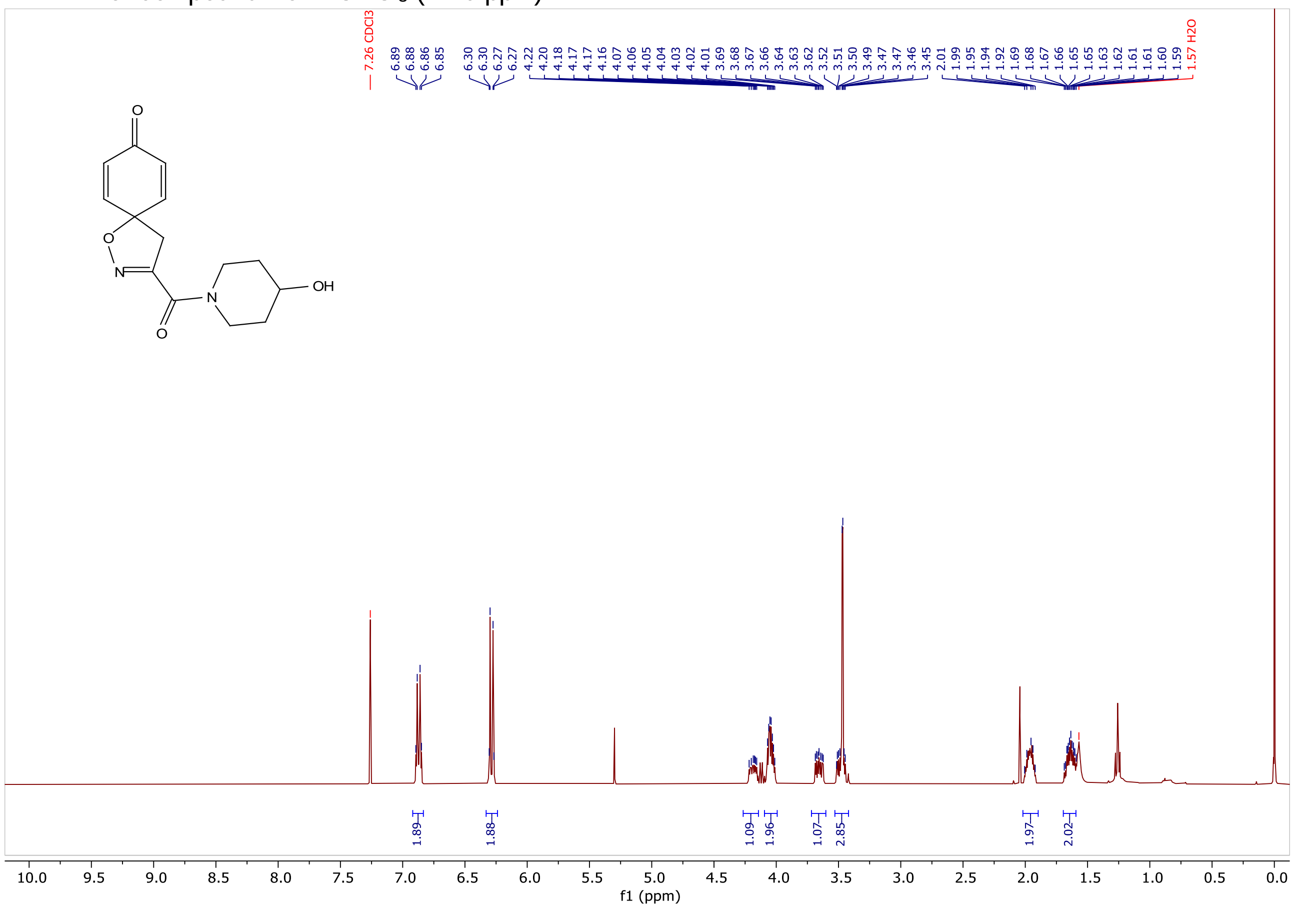

^{13}C NMR of compound **16** in $CDCl_3$ (77.16 ppm)

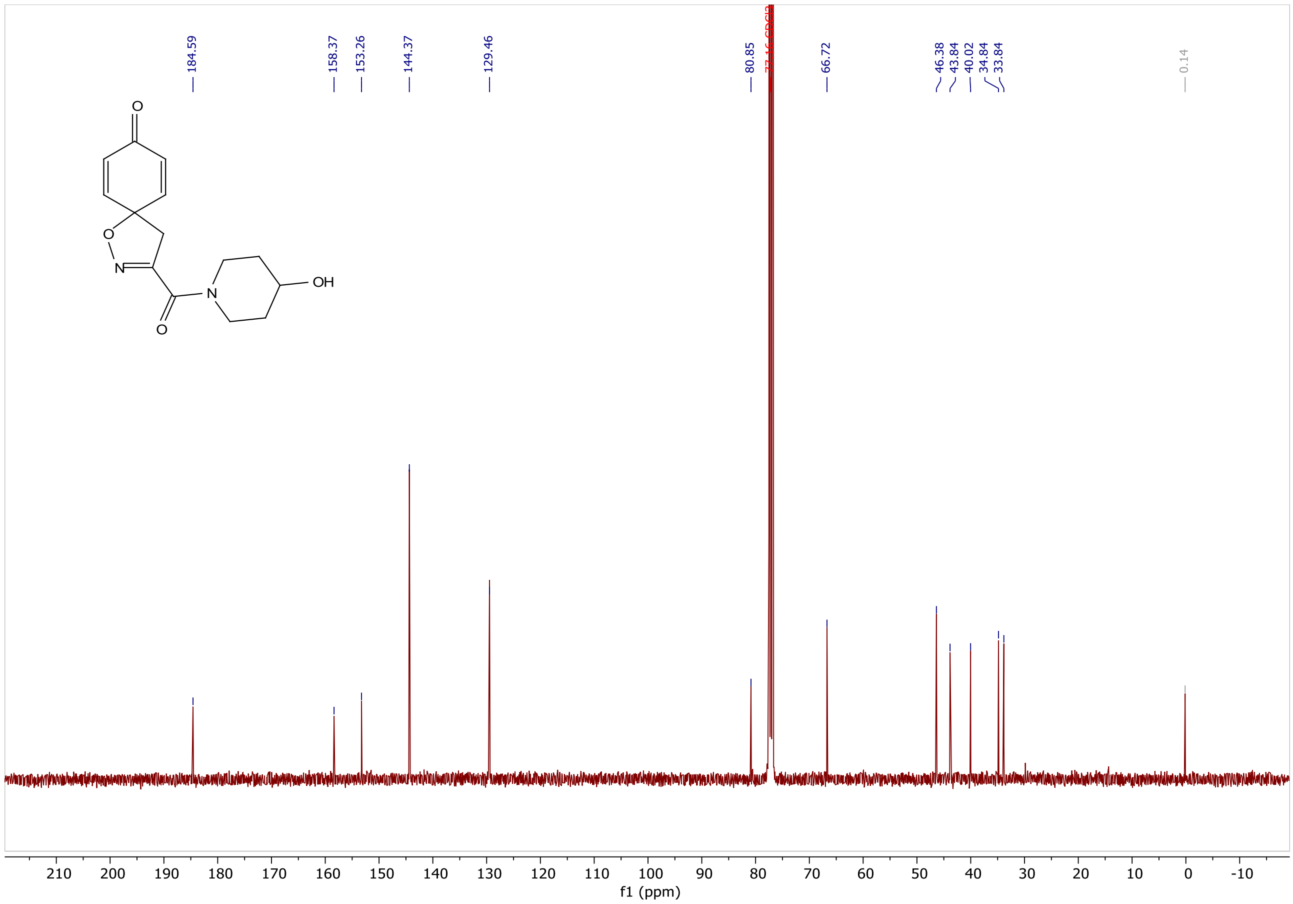

^{1}H NMR of compound **17** in CD_3OD (3.31 ppm)

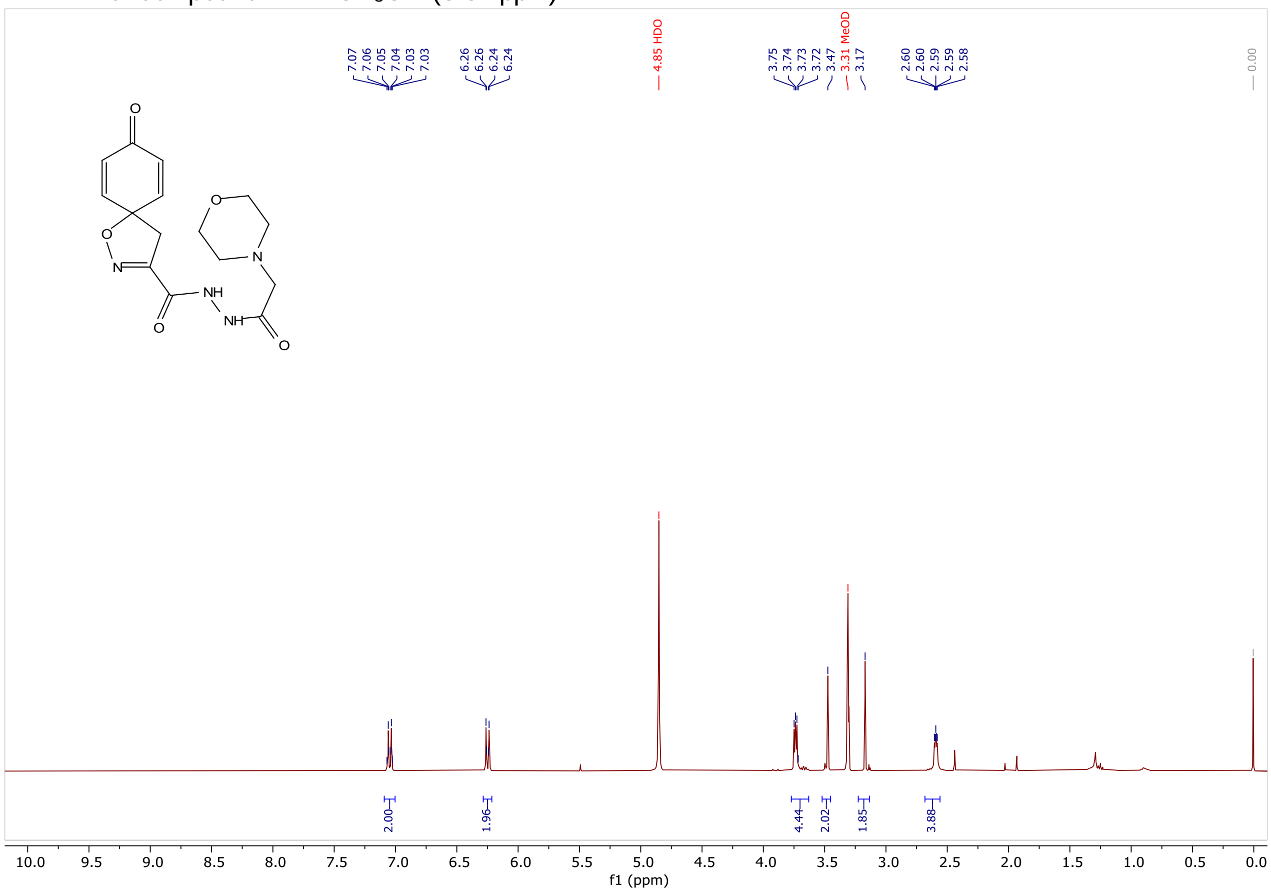

^{13}C NMR of compound **17** in CD_3OD (49.00 ppm)

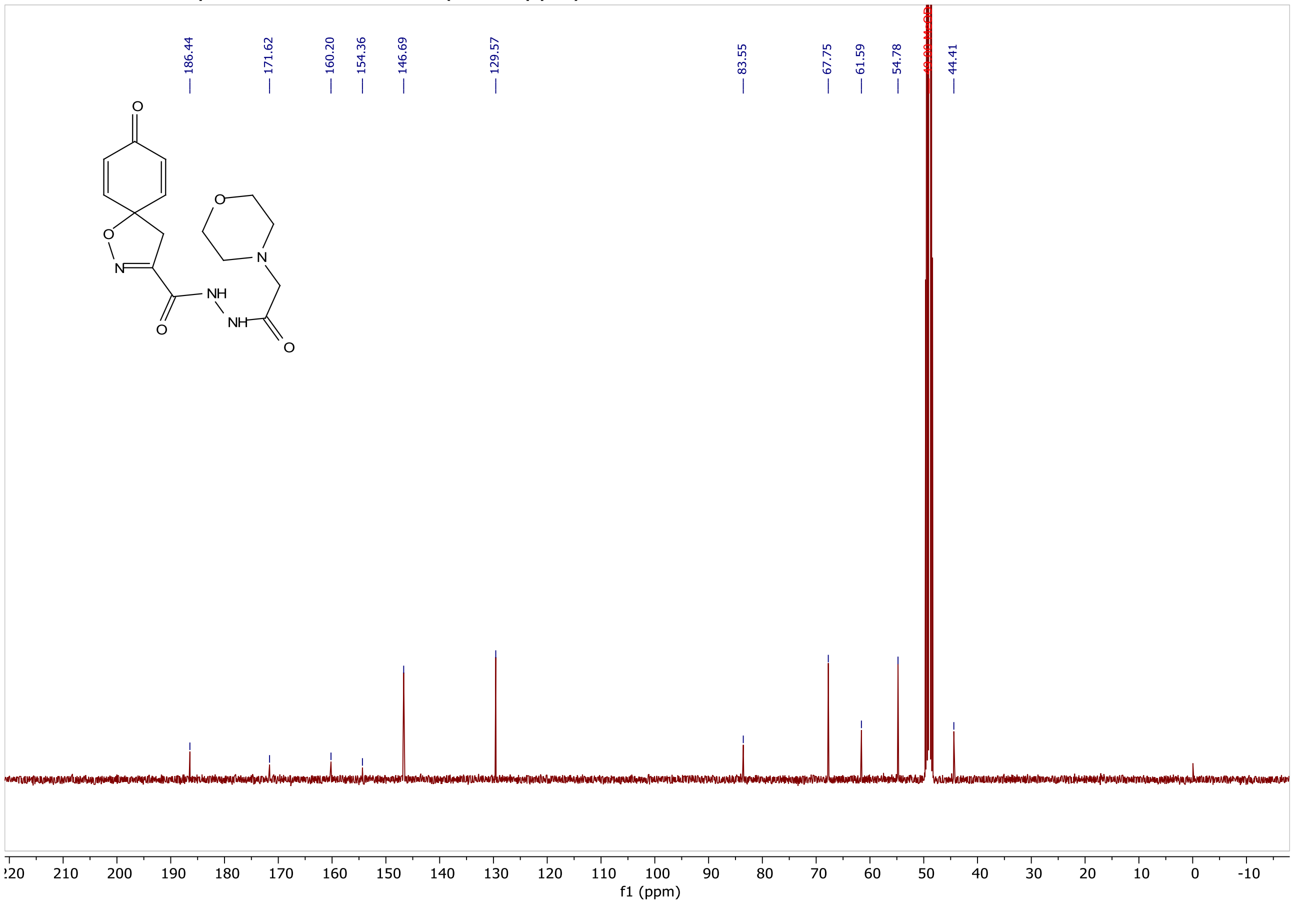

^{1}H NMR of compound **18** in $CDCl_3$ (7.26 ppm)

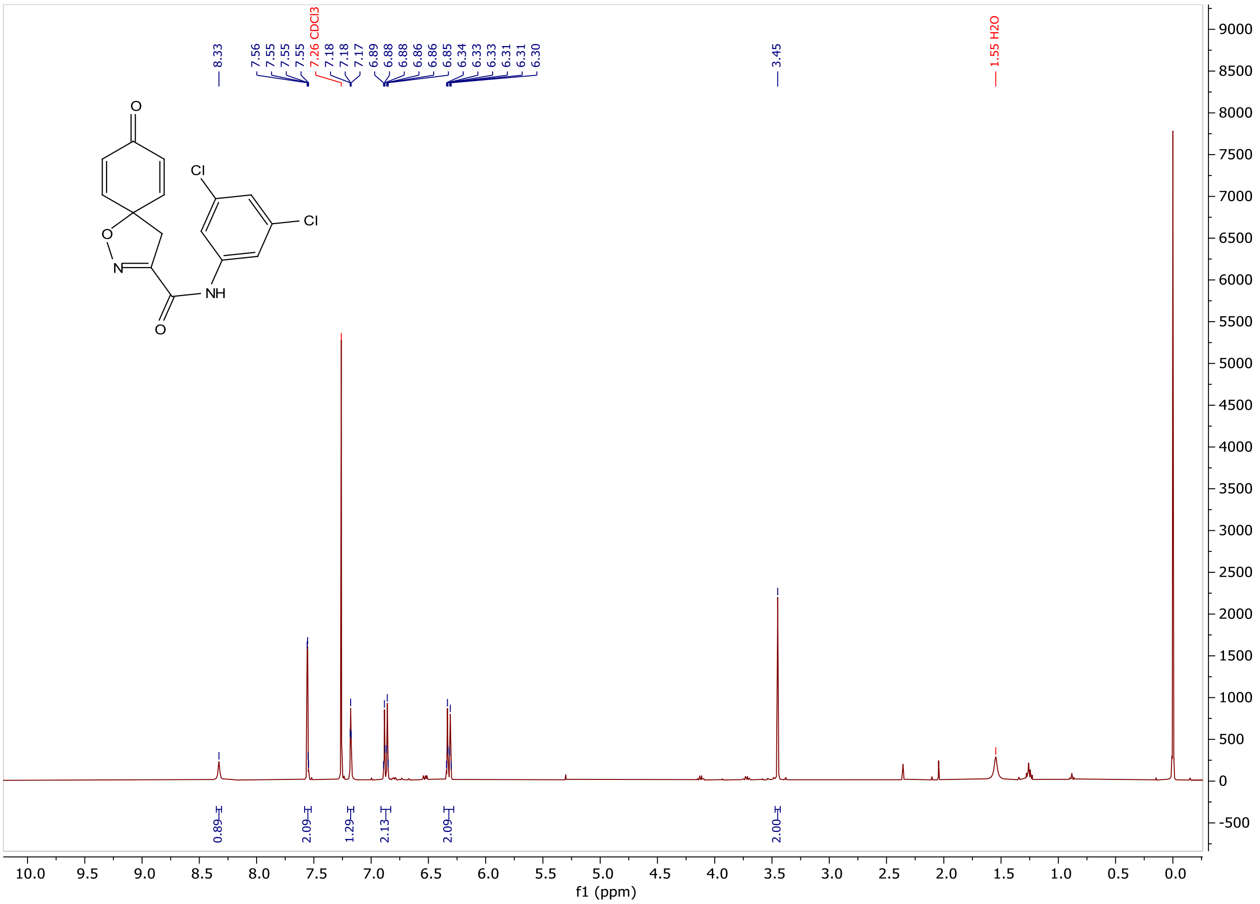

^{13}C NMR of compound **18** in $CDCl_3$ (77.16 ppm)

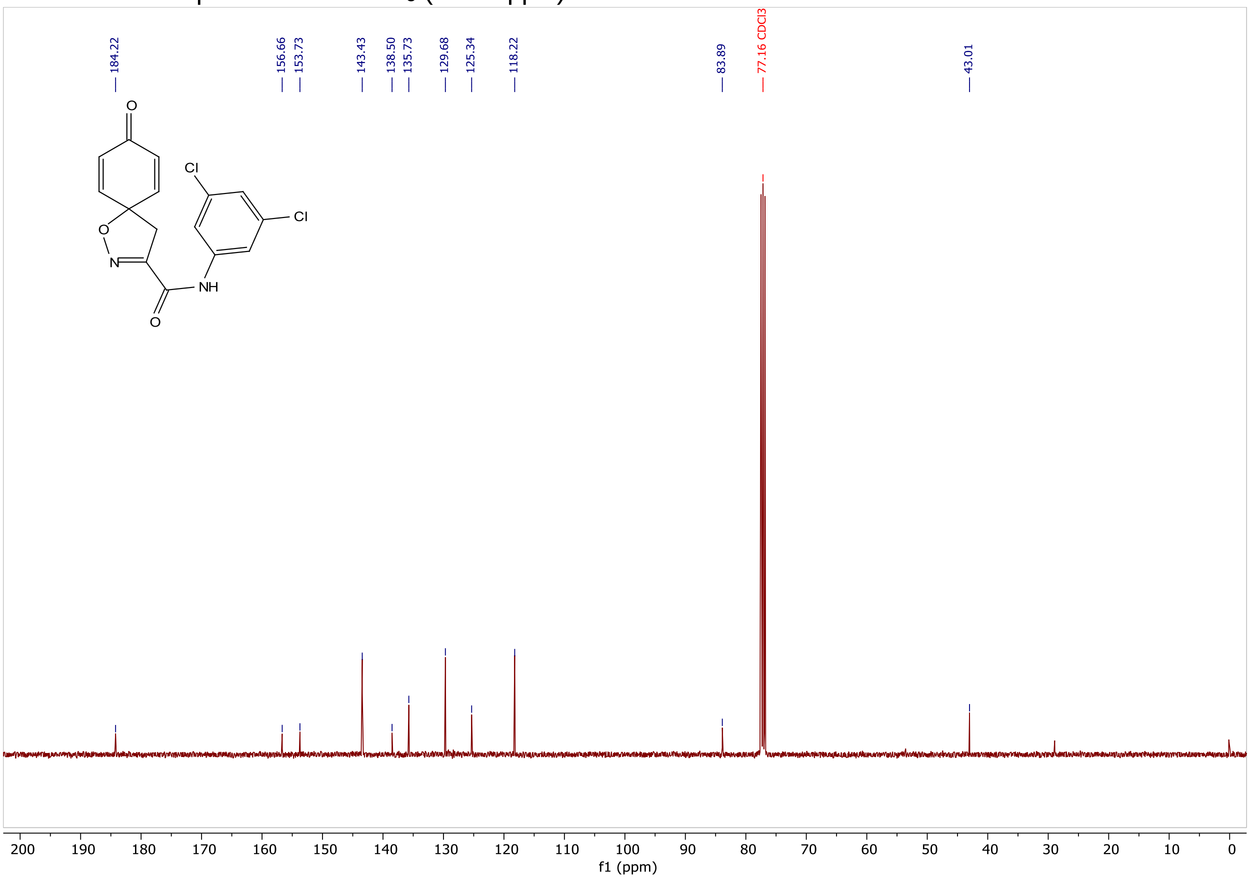

^{1}H NMR of compound **19** in d_6-acetone (2.05 ppm)

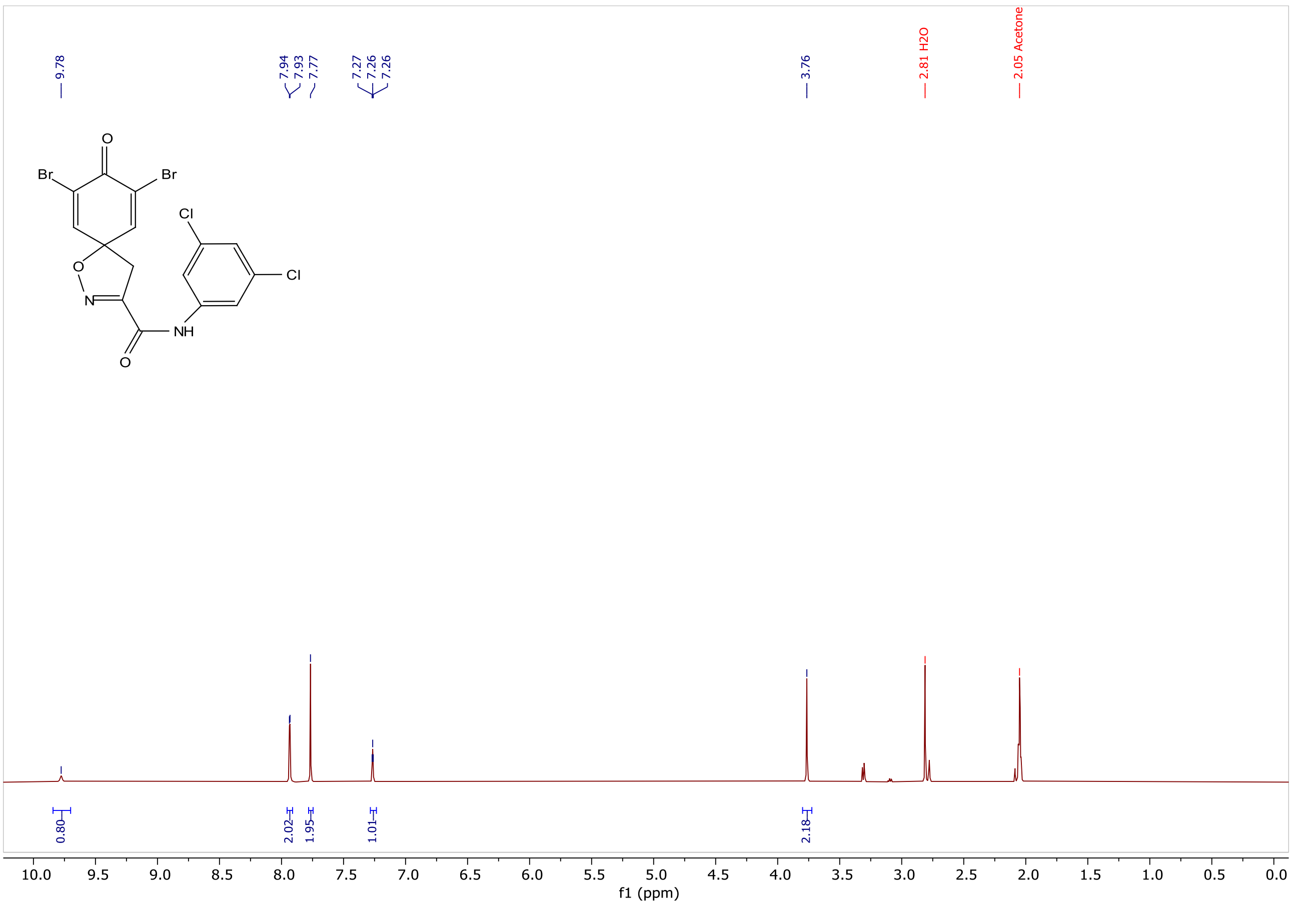

^{13}C NMR of compound **19** in d_6-acetone (29.84 ppm)

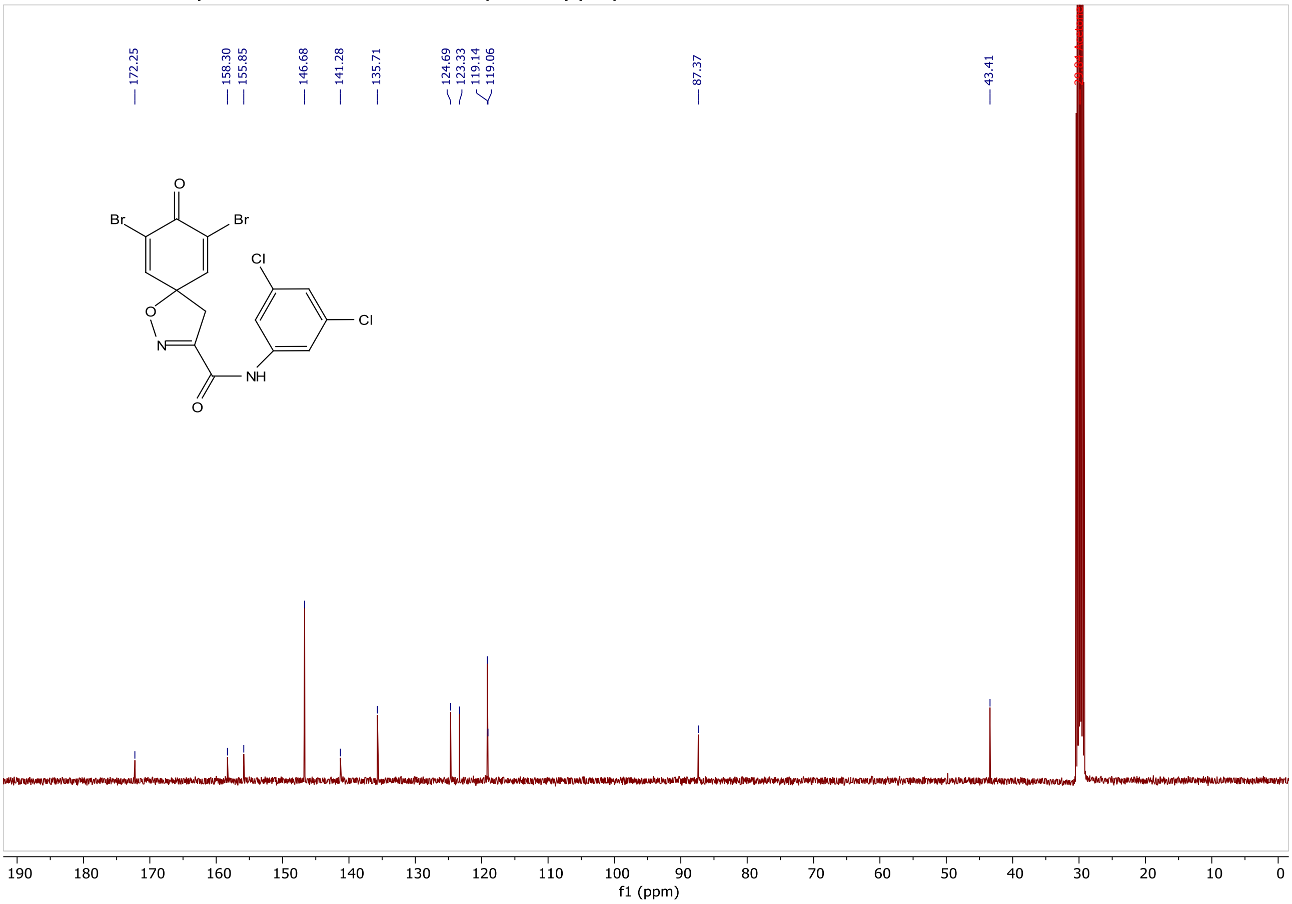

^{1}H NMR of compound **20** in $CDCl_3$ (7.26 ppm)

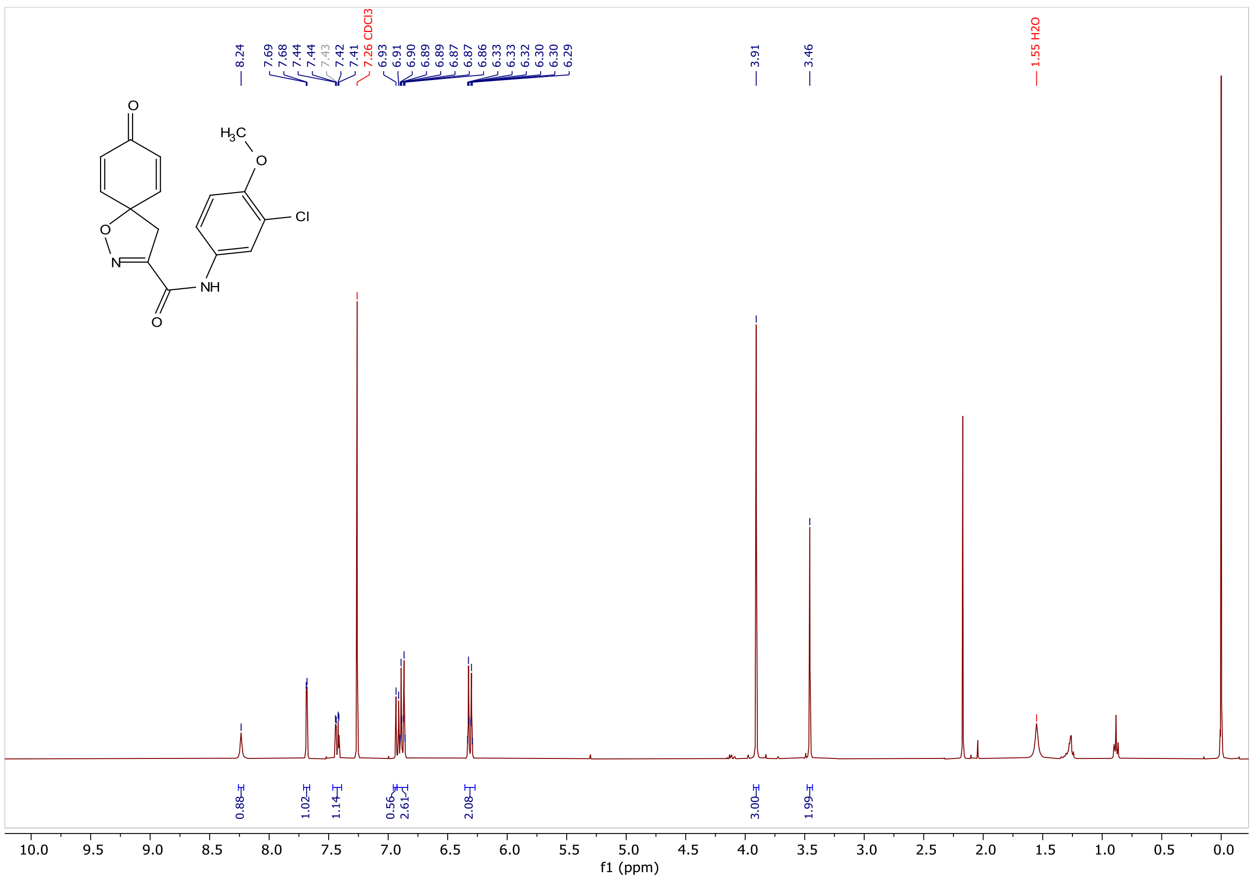

^{13}C NMR of compound **20** in $CDCl_3$ (77.16 ppm)

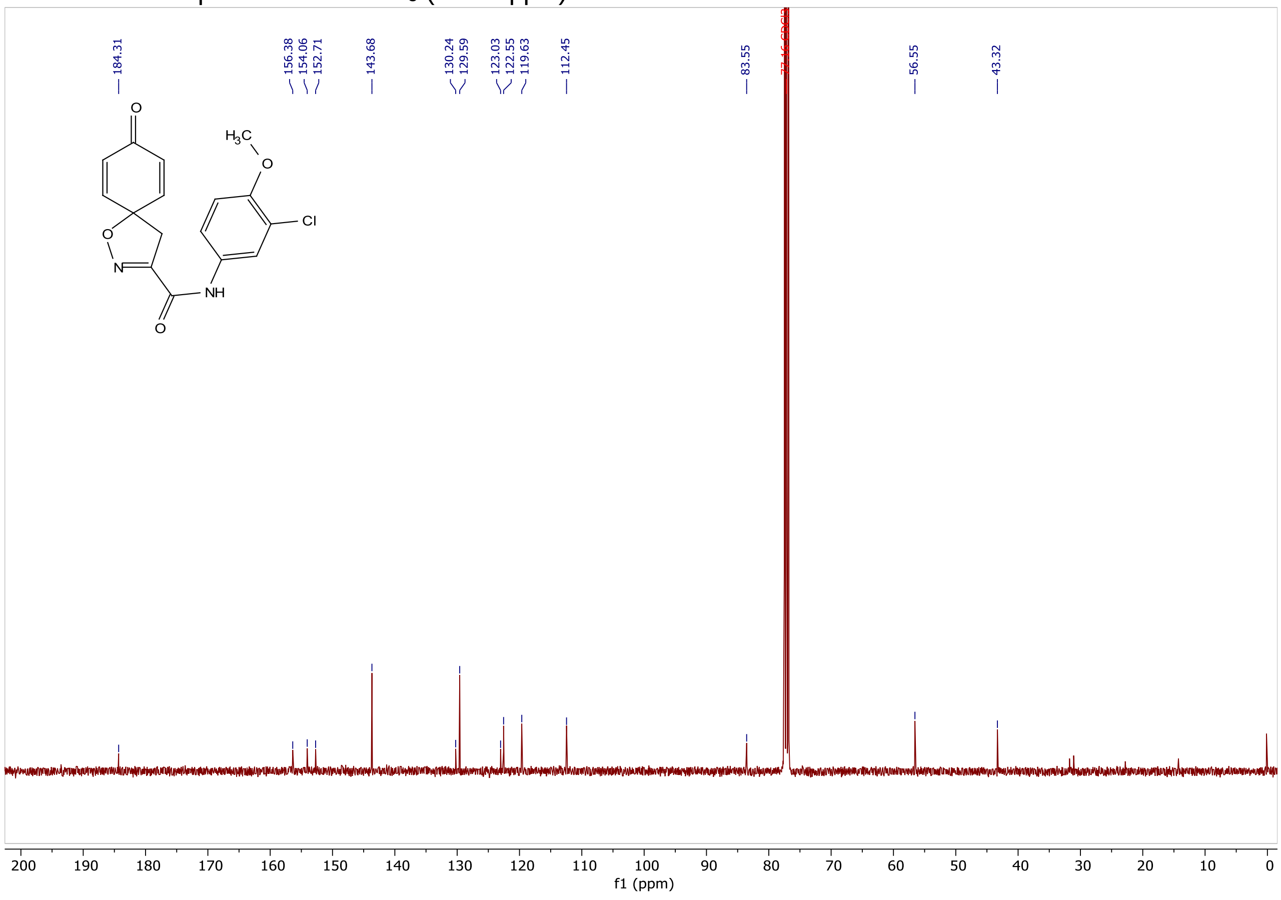

^{1}H NMR of compound **21** in d_6-DMSO (2.50 ppm)

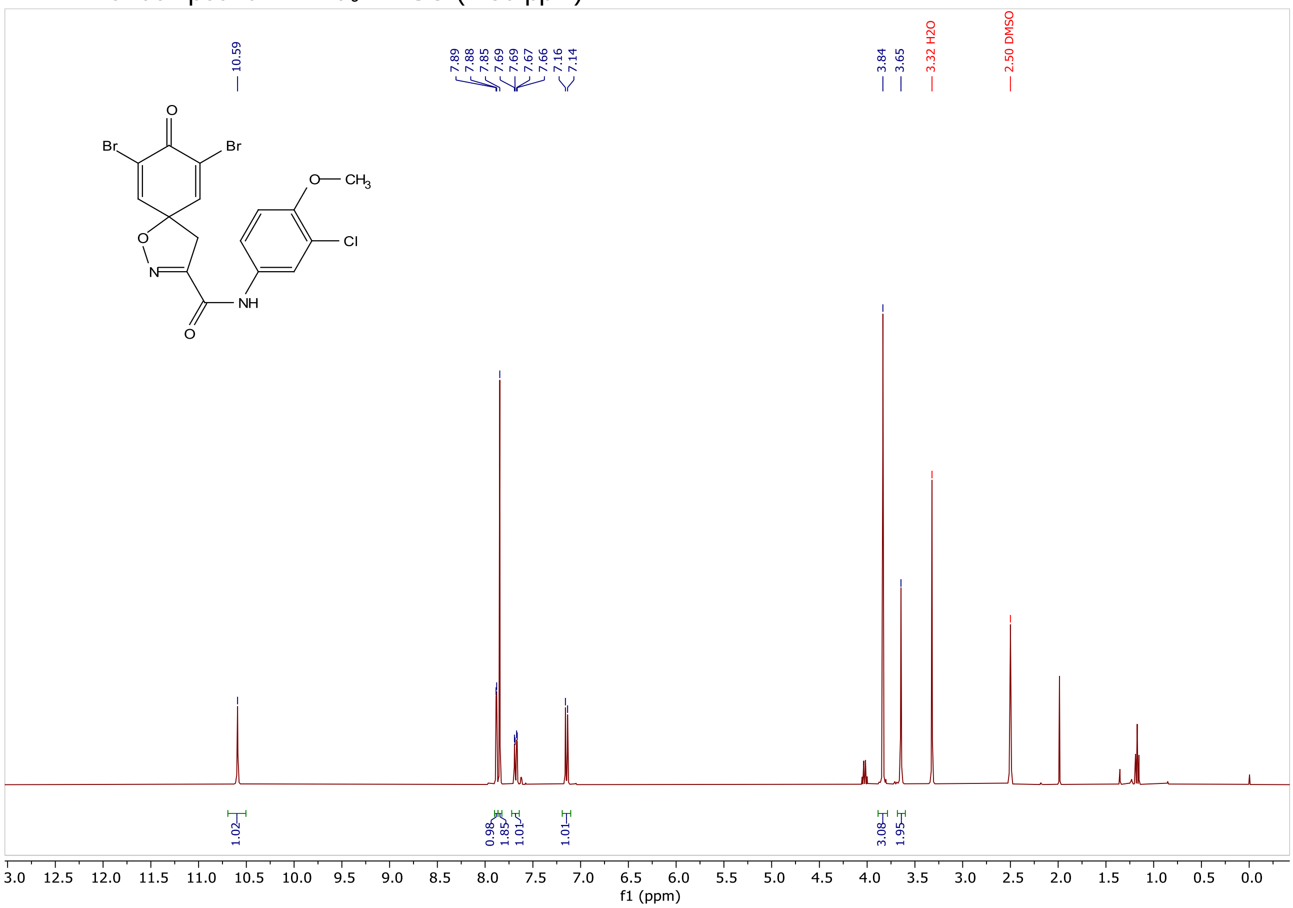

^{13}C NMR of compound **21** in d_6-DMSO (39.52 ppm)

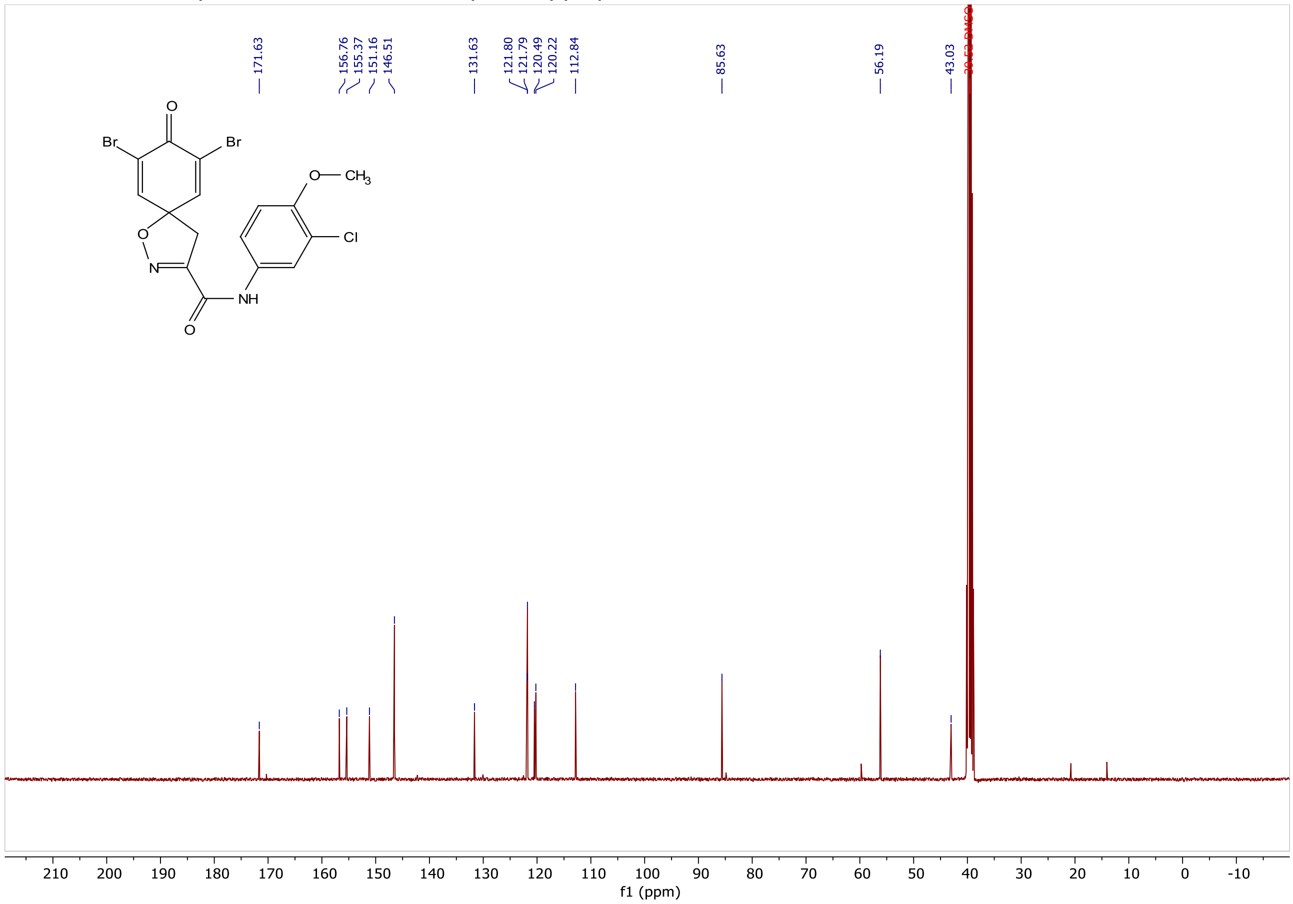

^{1}H NMR of compound **22** in $CDCl_3$ (7.26 ppm)

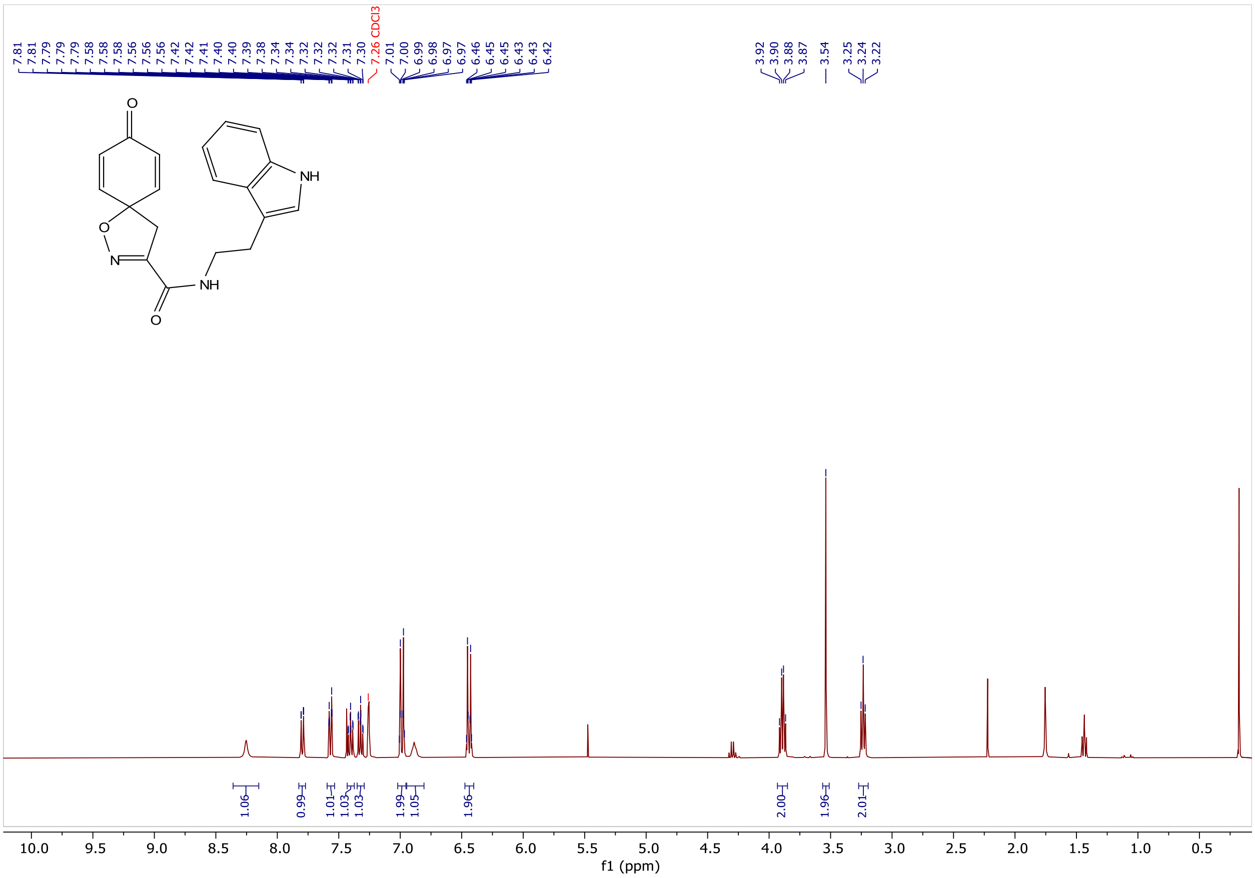

^{13}C NMR of compound **22** in $CDCl_3$ (77.16 ppm)

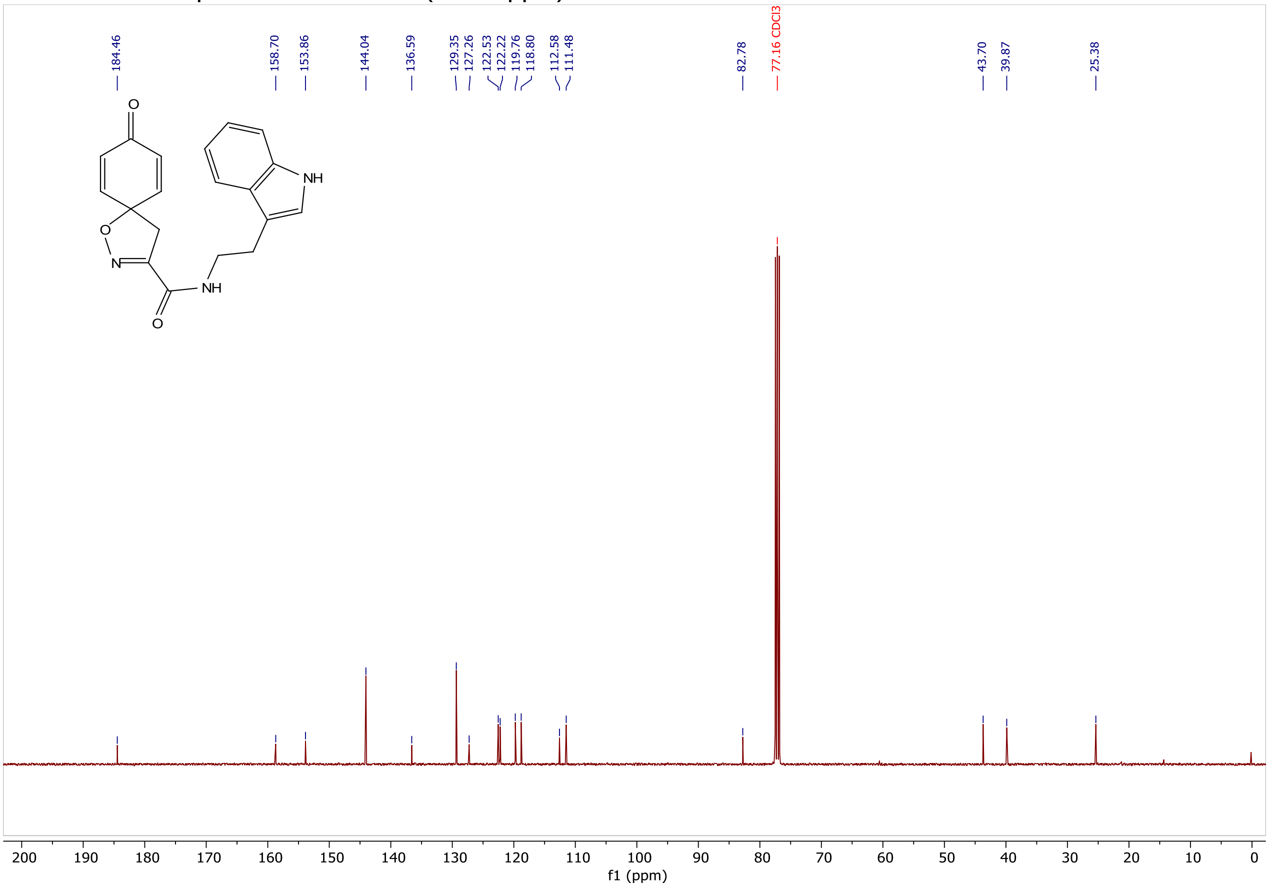

^{1}H NMR of compound **23** in $CDCl_3$ (7.26 ppm)

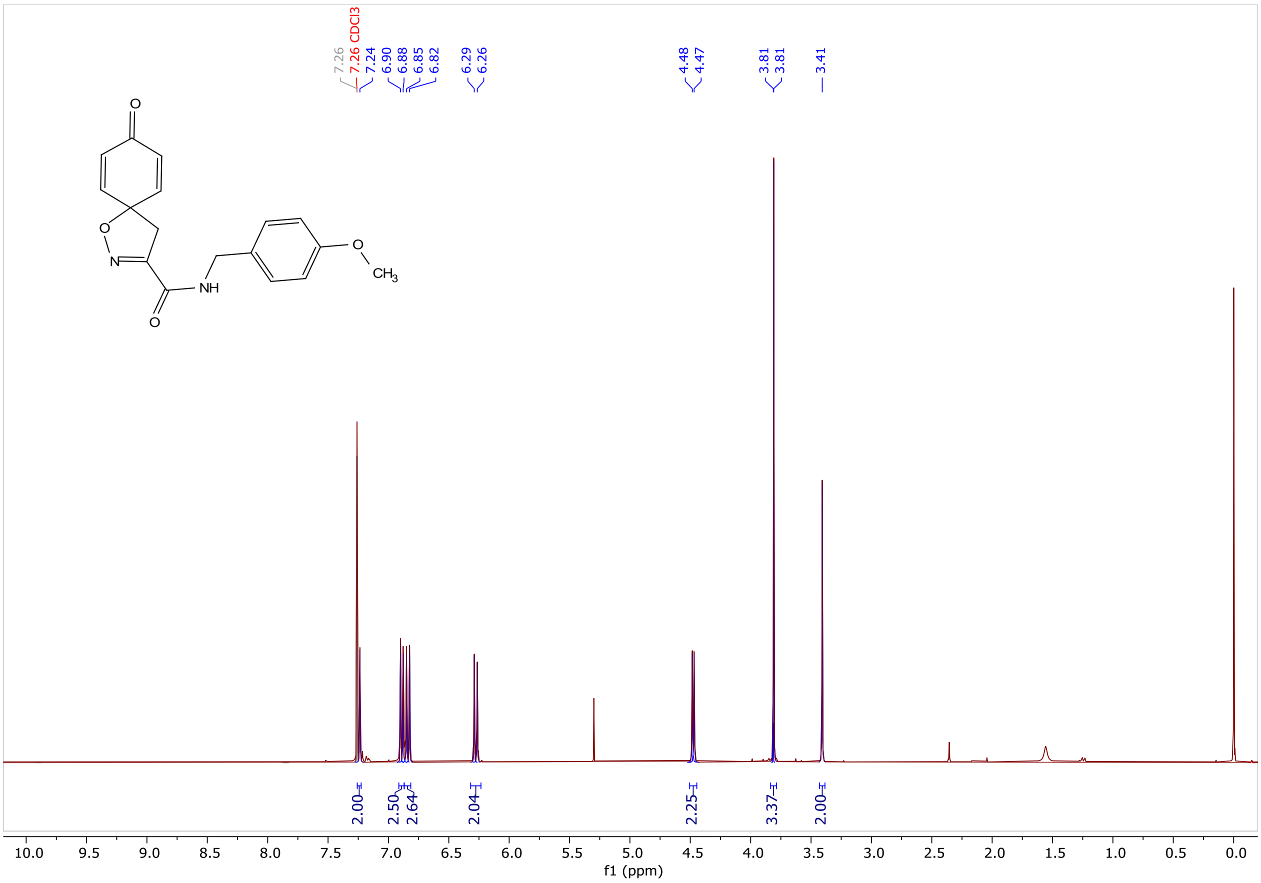

^{13}C NMR of compound **23** in $CDCl_3$ (77.16 ppm)

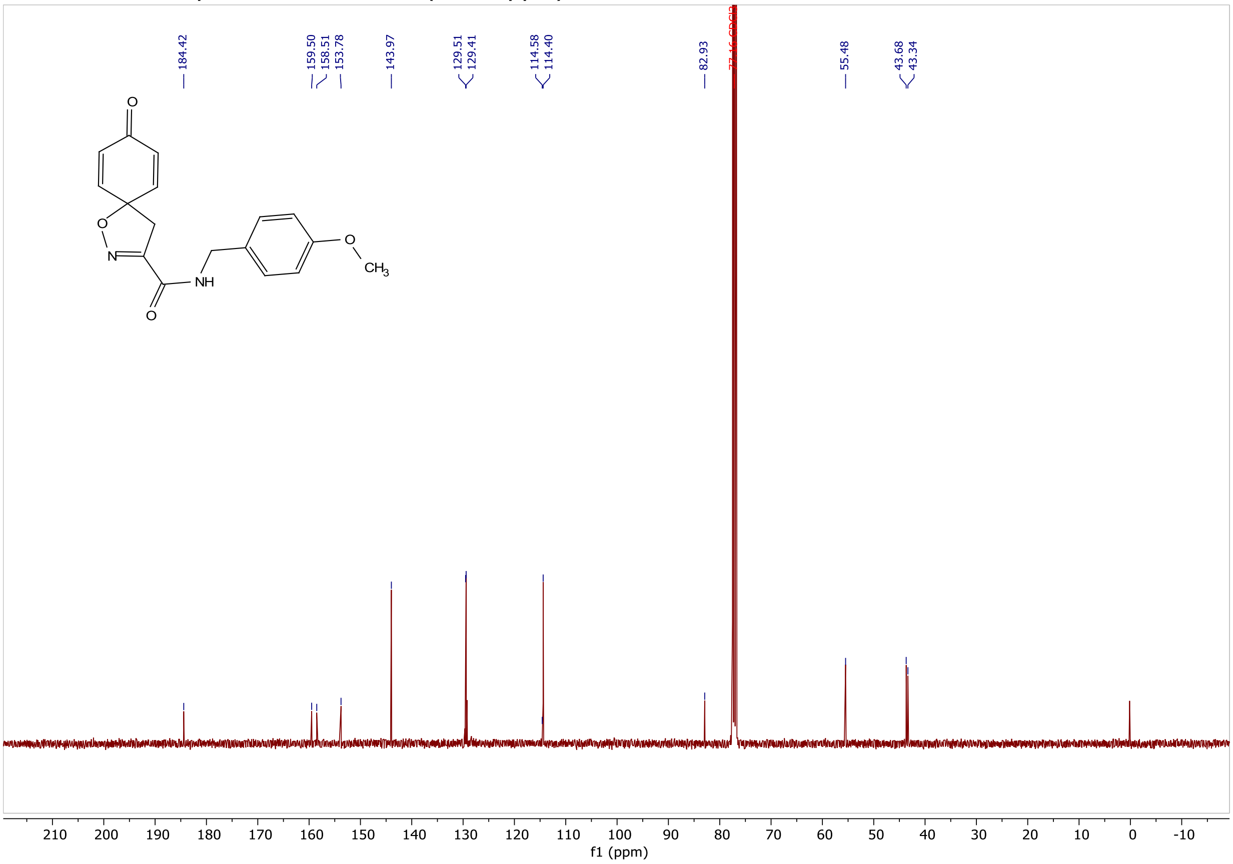

^{1}H NMR of compound **24** in d_6-DMSO (2.50 ppm)

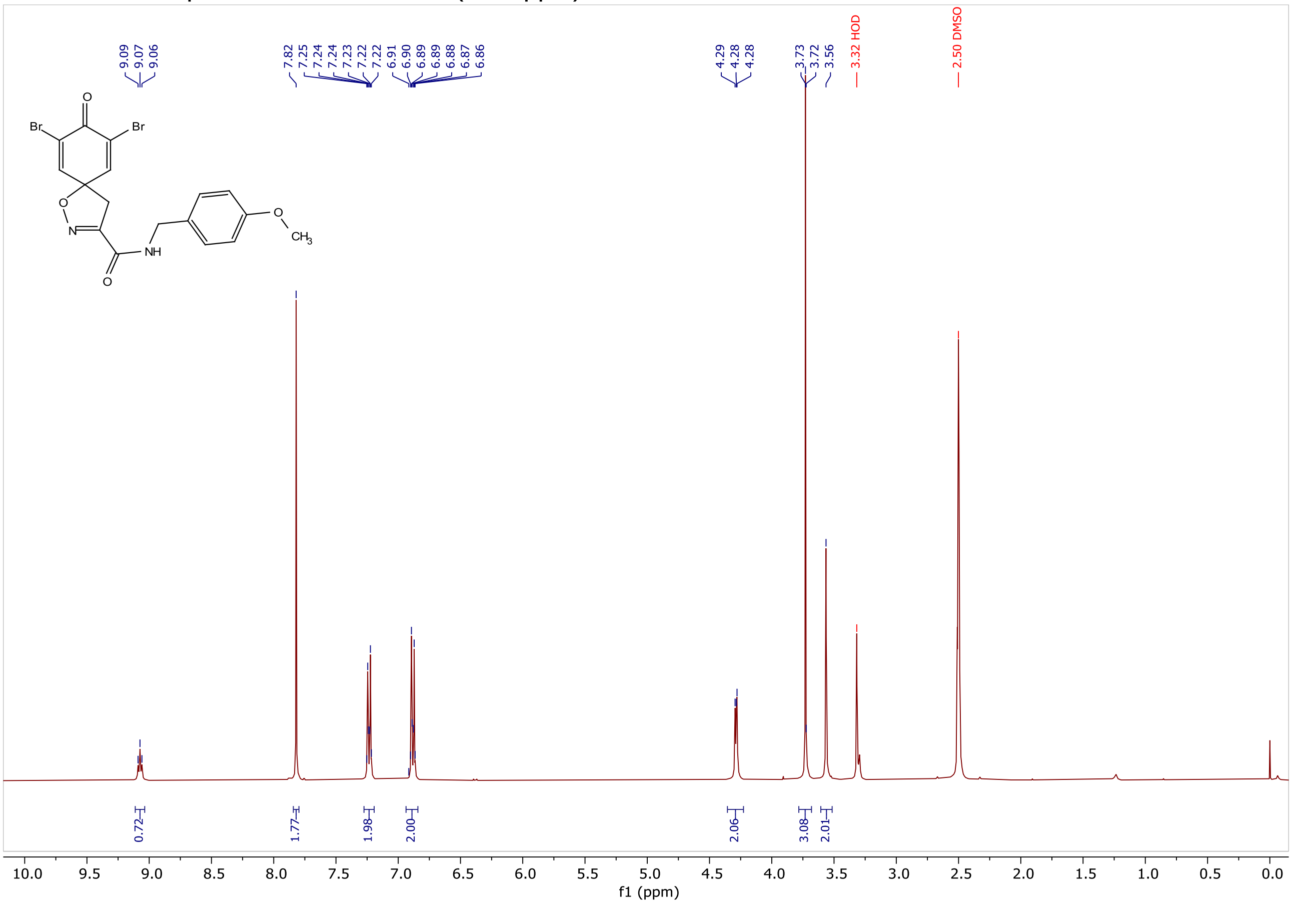

^{13}C NMR of compound **24** in d_6-DMSO (39.52 ppm)

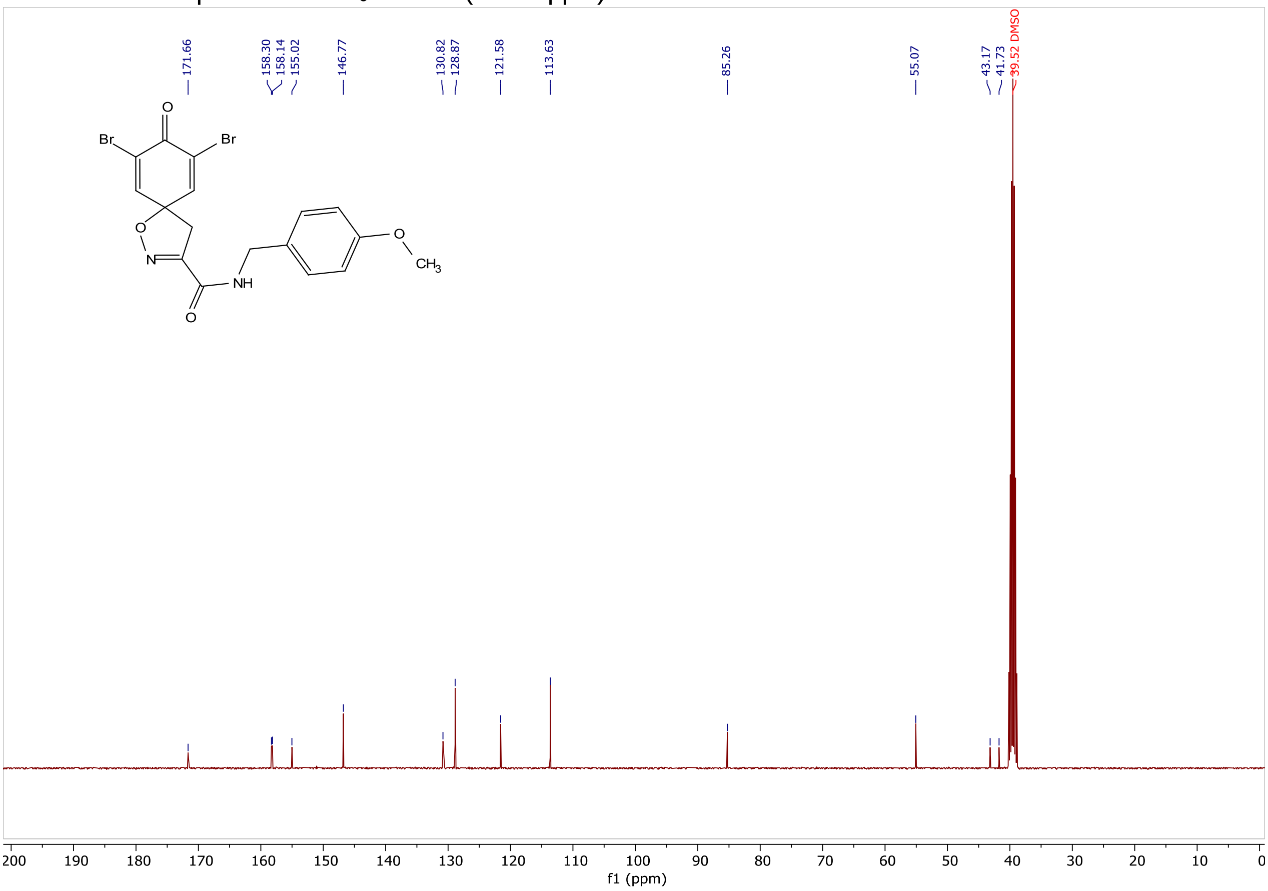

^{1}H NMR of compound **25** in $CDCl_3$ (7.26 ppm)

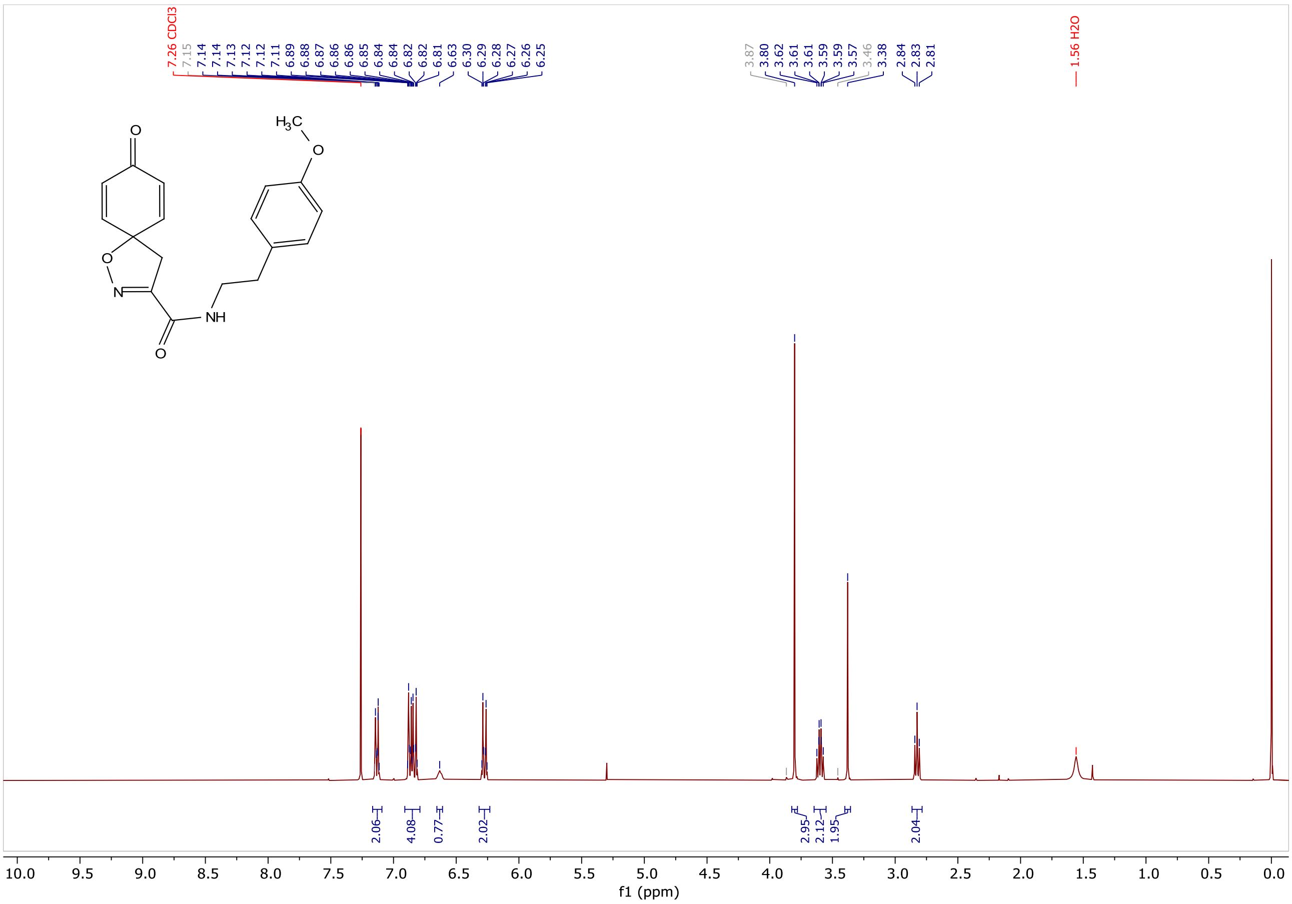

^{13}C NMR of compound **25** in $CDCl_3$ (77.16 ppm)

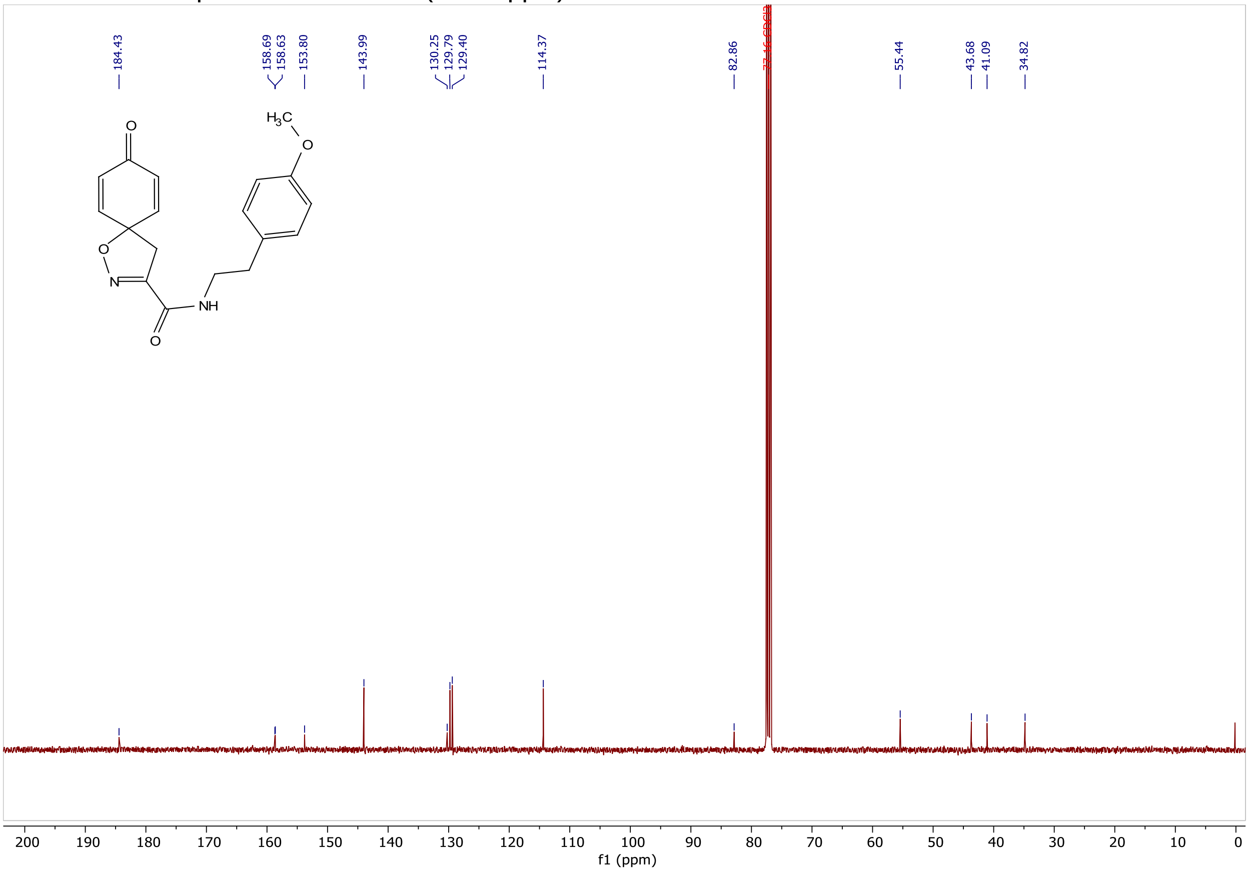

^{1}H NMR of compound **26** in $CDCl_3$ (7.26 ppm)

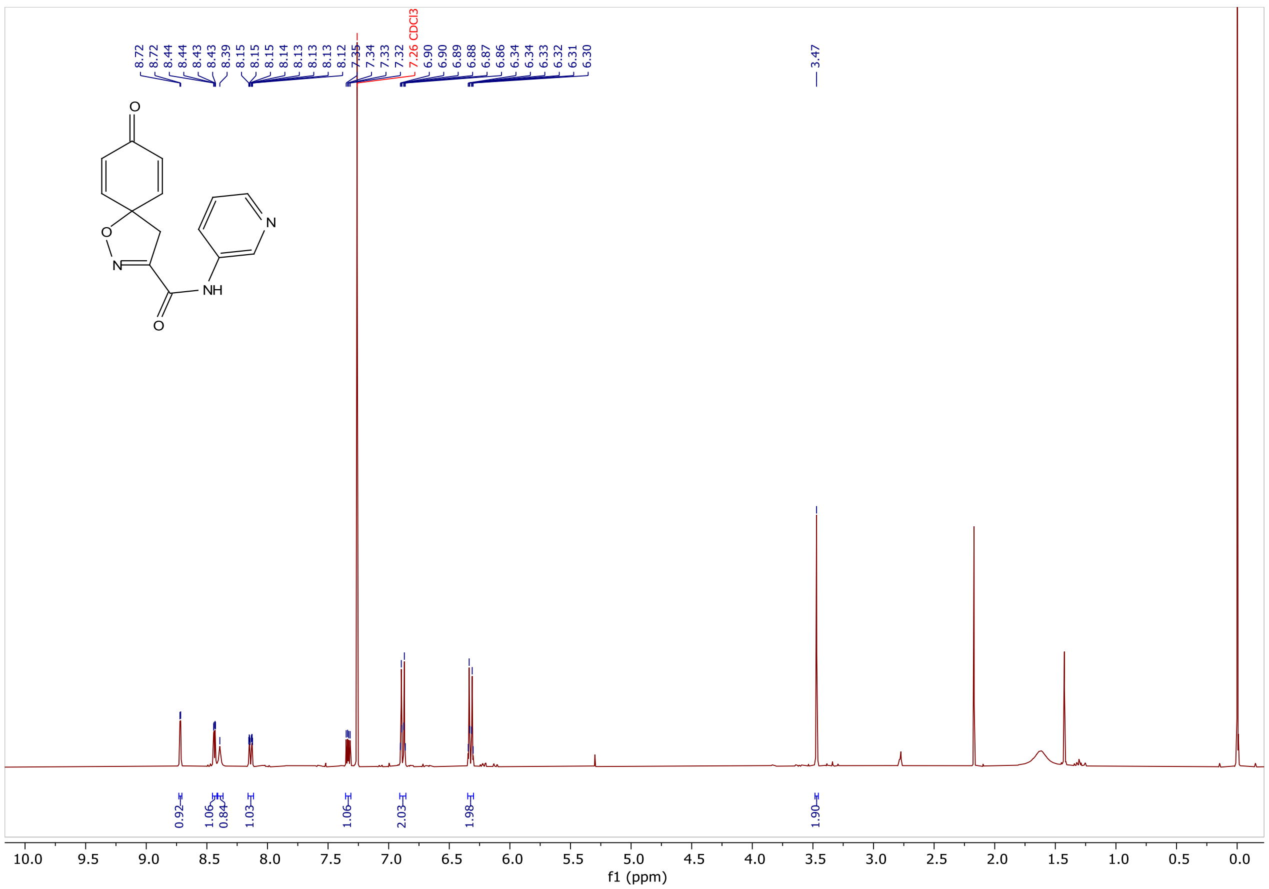

^{13}C NMR of compound **26** in $CDCl_3$ (77.16 ppm)

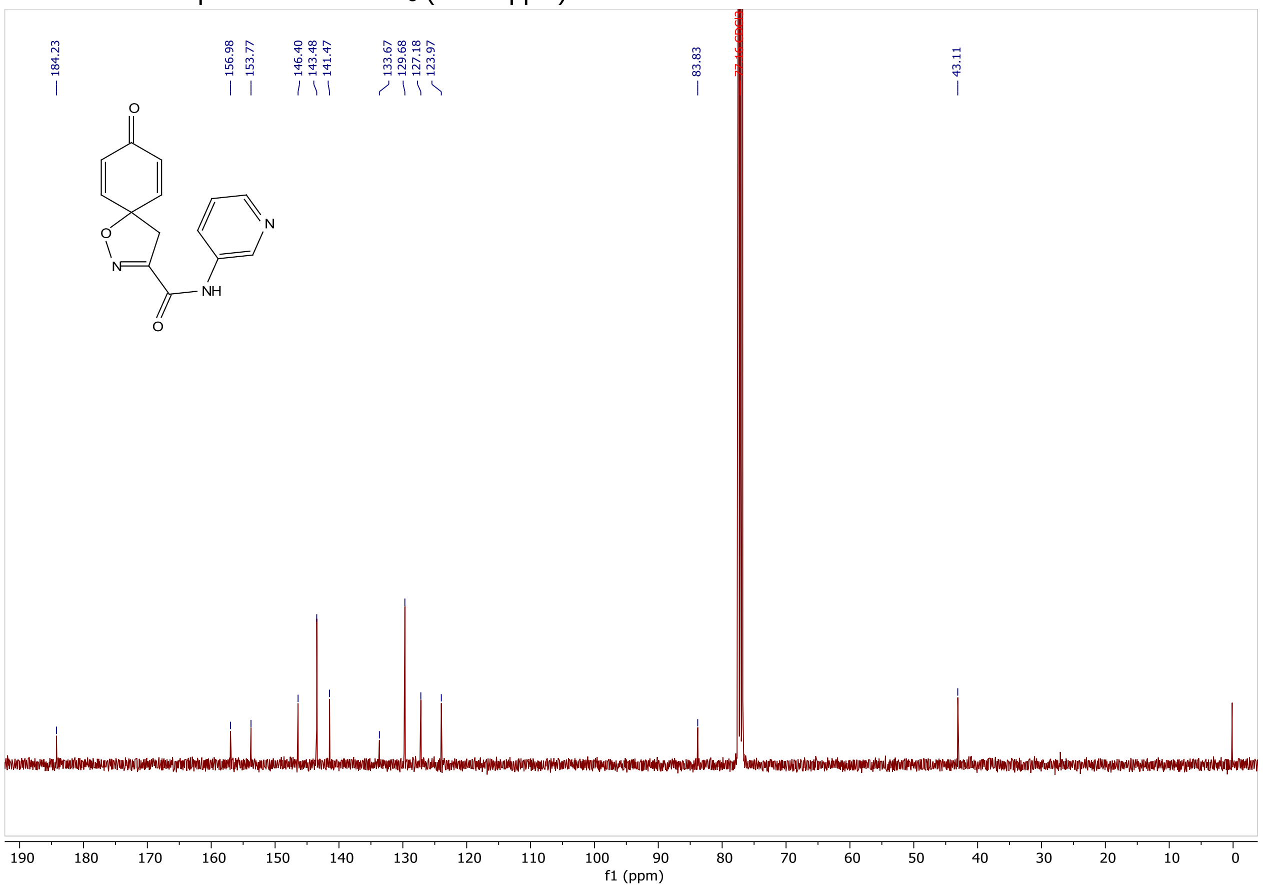

^{1}H NMR of compound **27** in d_6-DMSO (2.50 ppm)

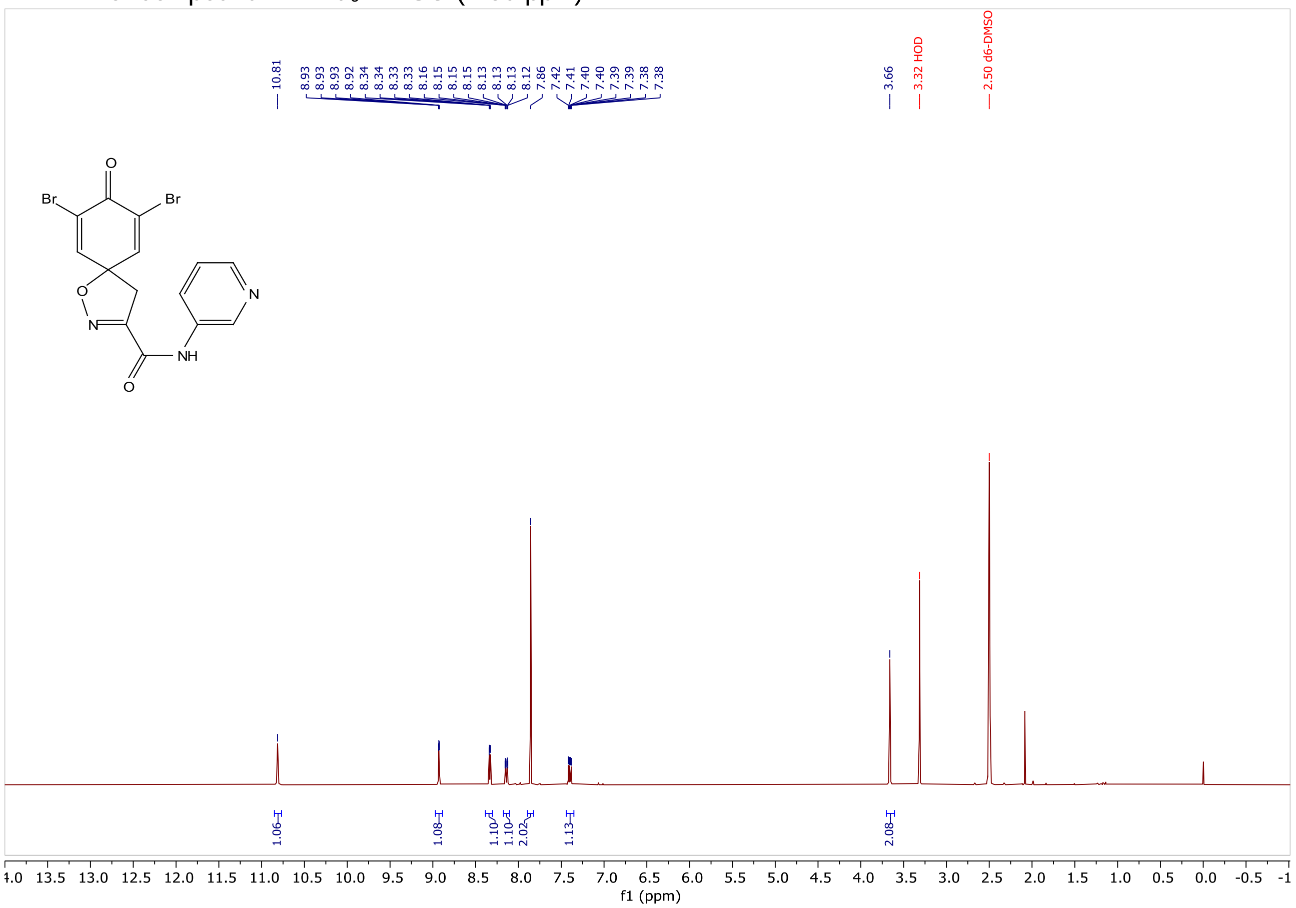

^{13}C NMR of compound **27** in d_6-DMSO (39.52 ppm)

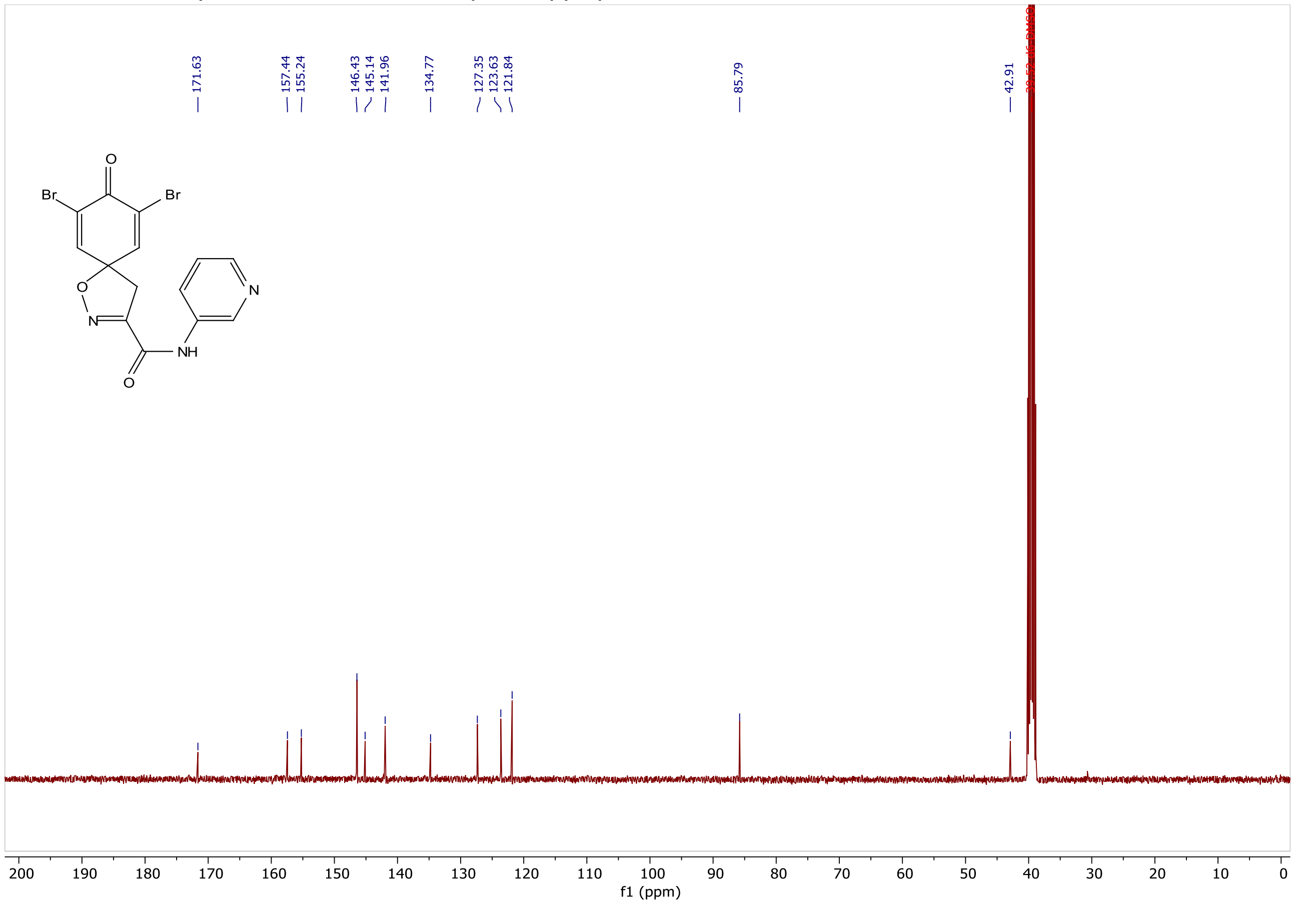

^{1}H NMR of compound **28** in $CDCl_3$ (7.26 ppm)

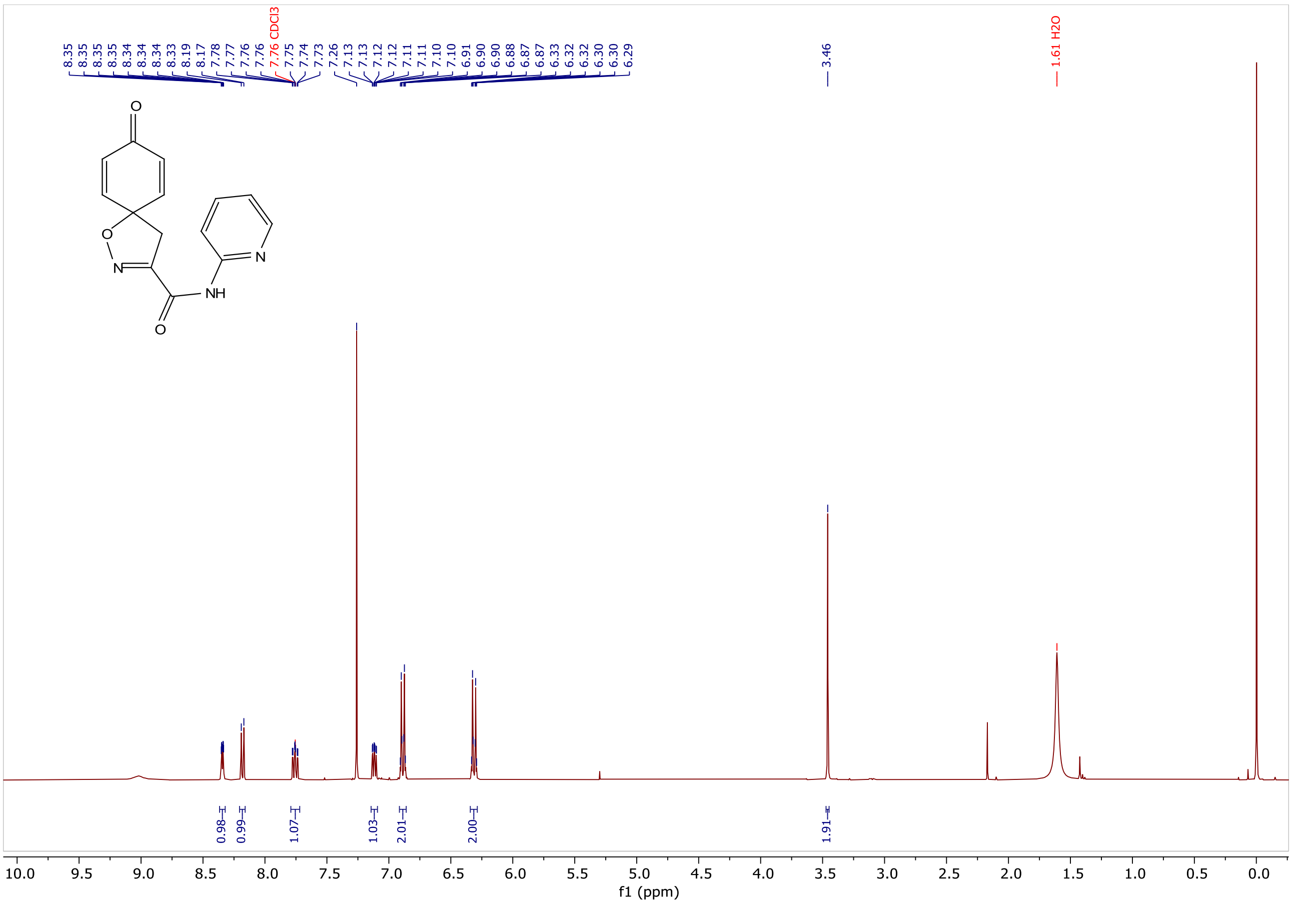

^{13}C NMR of compound **28** in $CDCl_3$ (77.16 ppm)

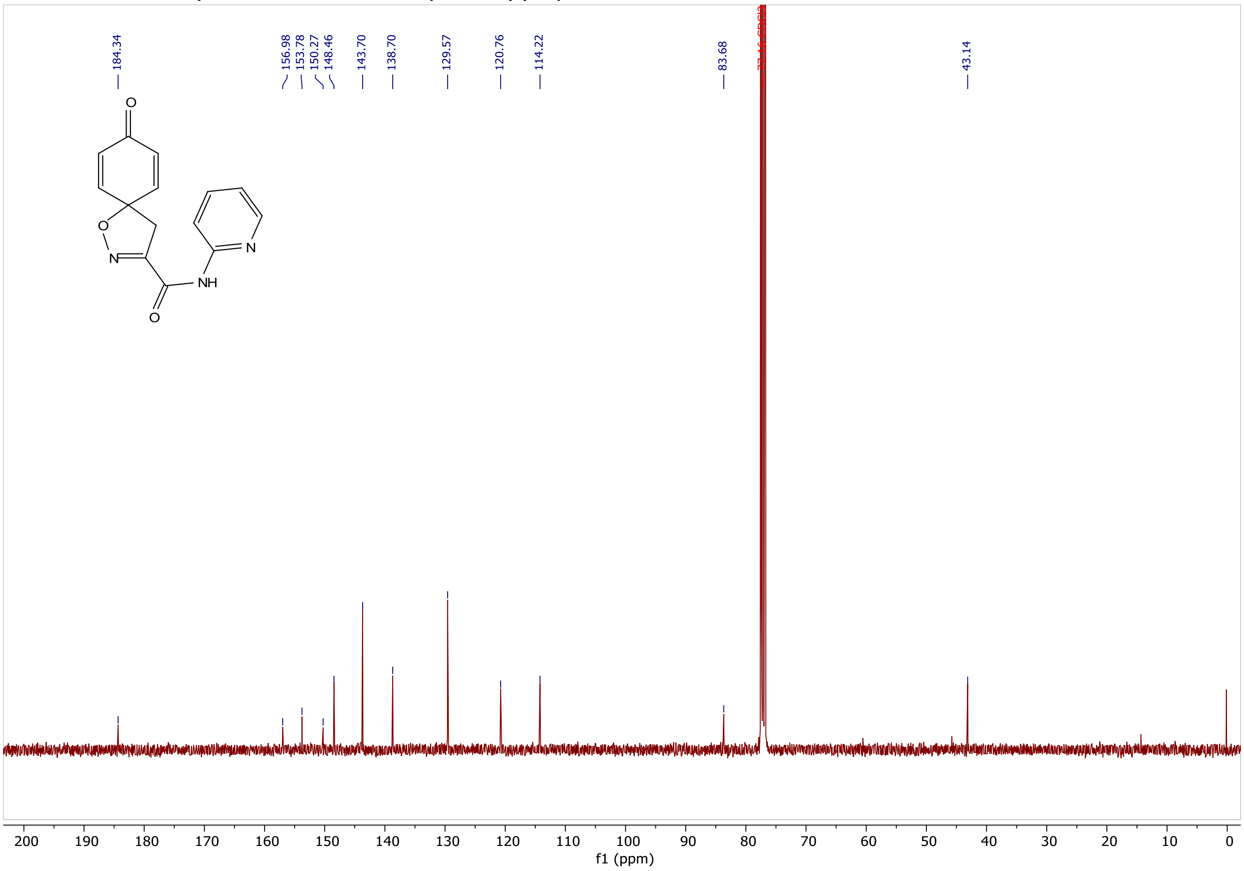

^{1}H NMR of compound **29** in d_6-acetone (2.05 ppm)

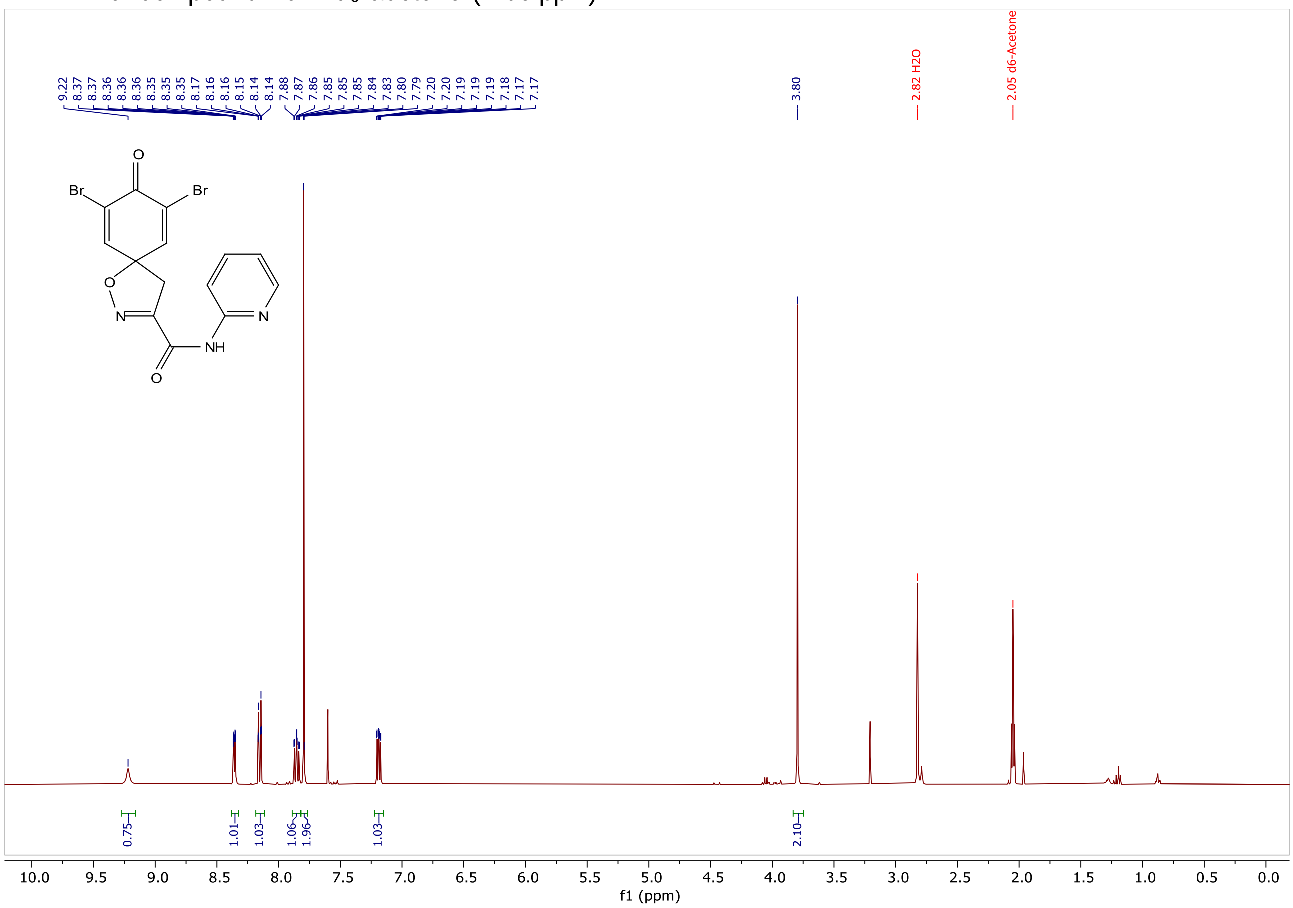

^{13}C NMR of compound **29** in d_6-acetone (29.84 ppm)

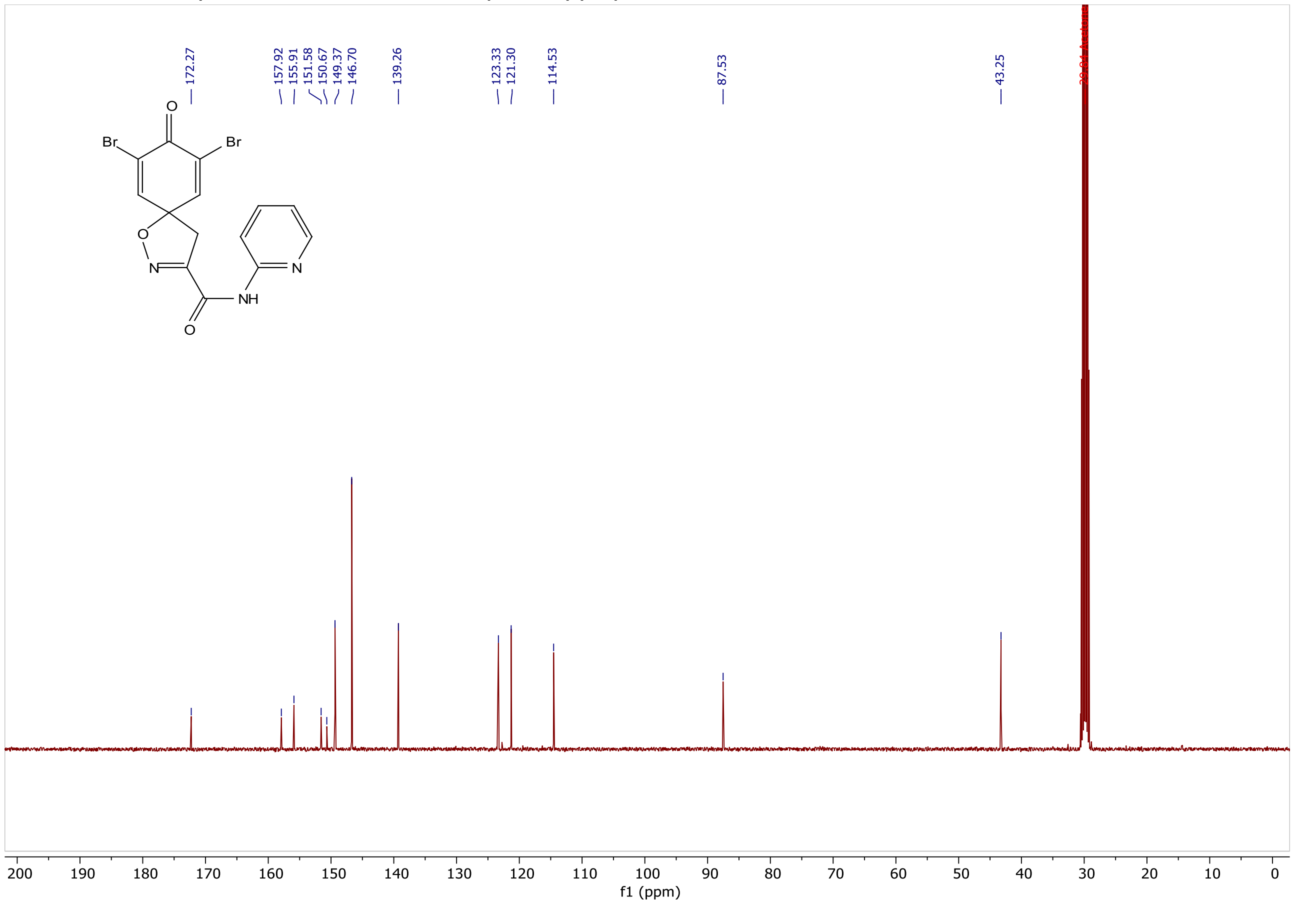

^{1}H NMR of compound **30** in $CDCl_3$ (7.26 ppm)

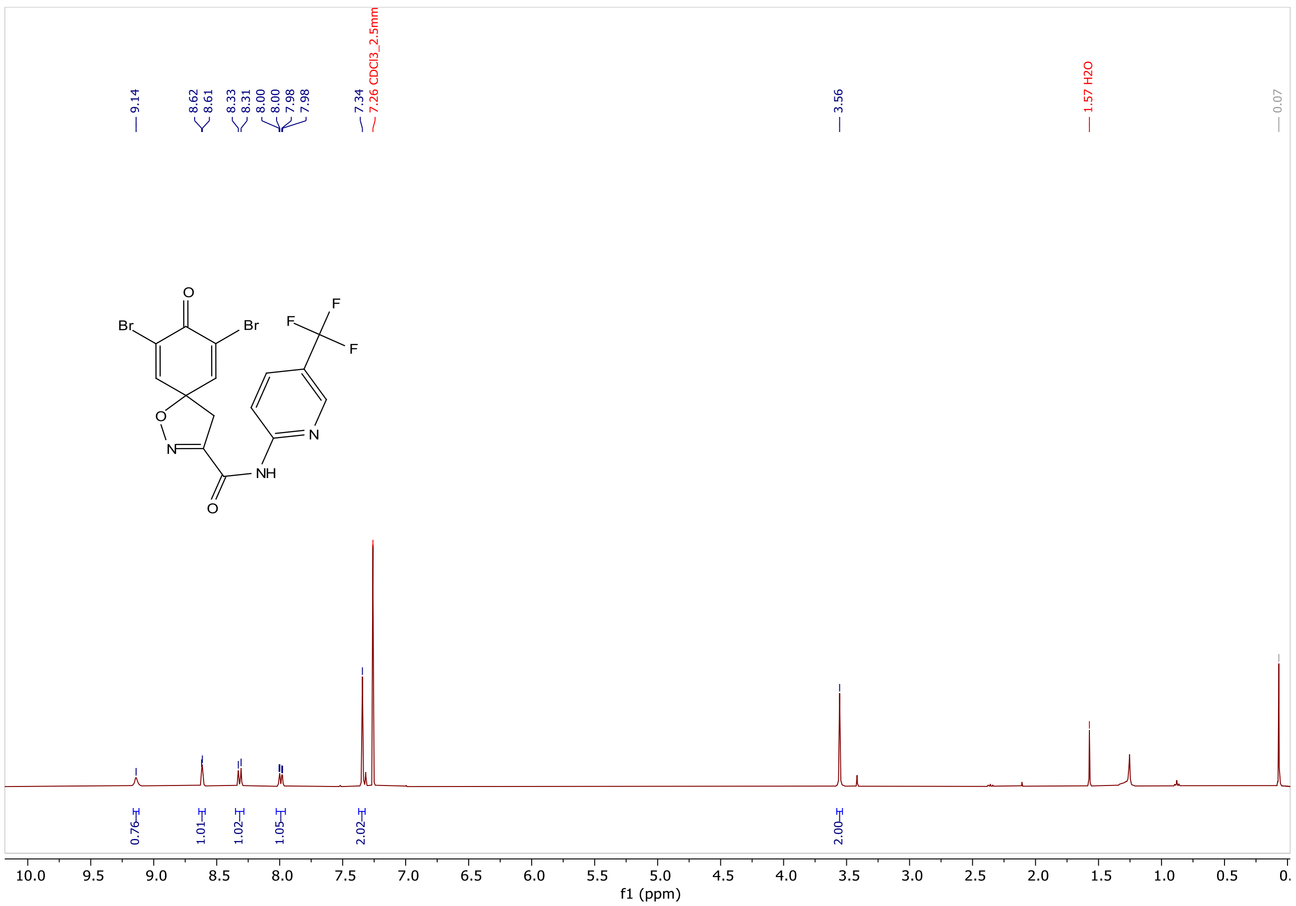

^{13}C NMR of compound **30** in $CDCl_3$ (77.16 ppm)

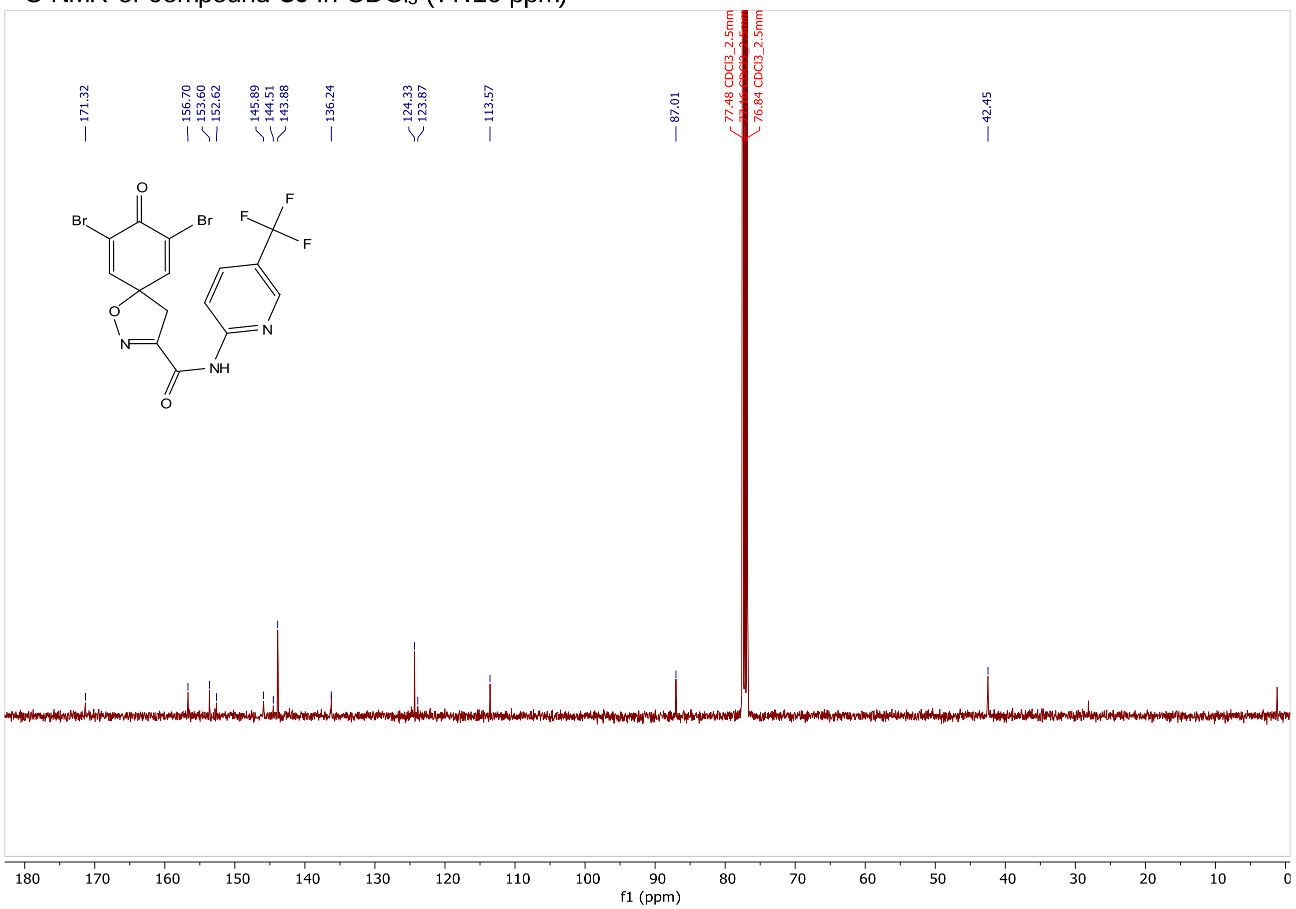

^{1}H NMR of compound **31** in d_6-acetone (2.05 ppm)

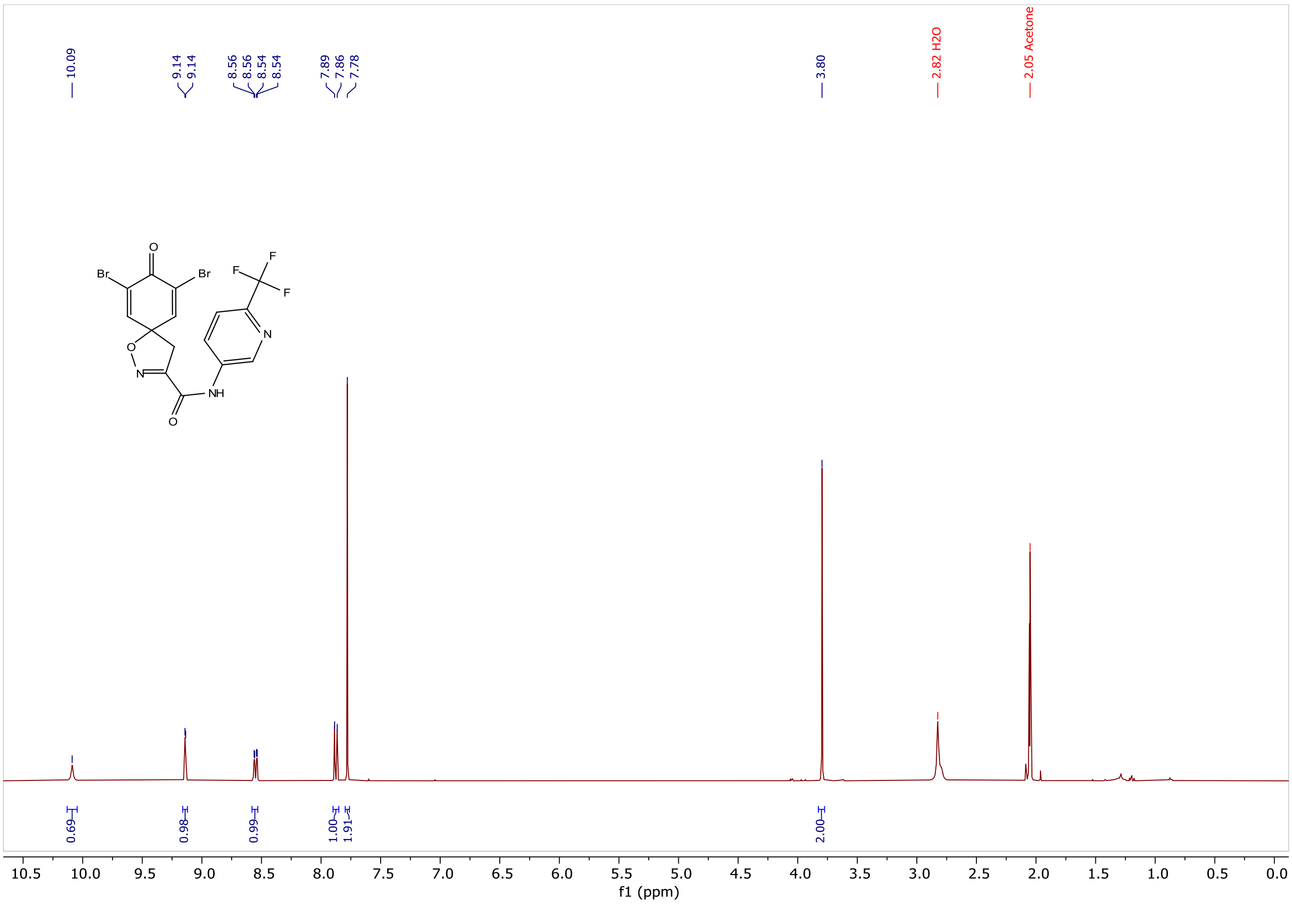

^{13}C NMR of compound **31** in d_6-acetone (29.84 ppm)

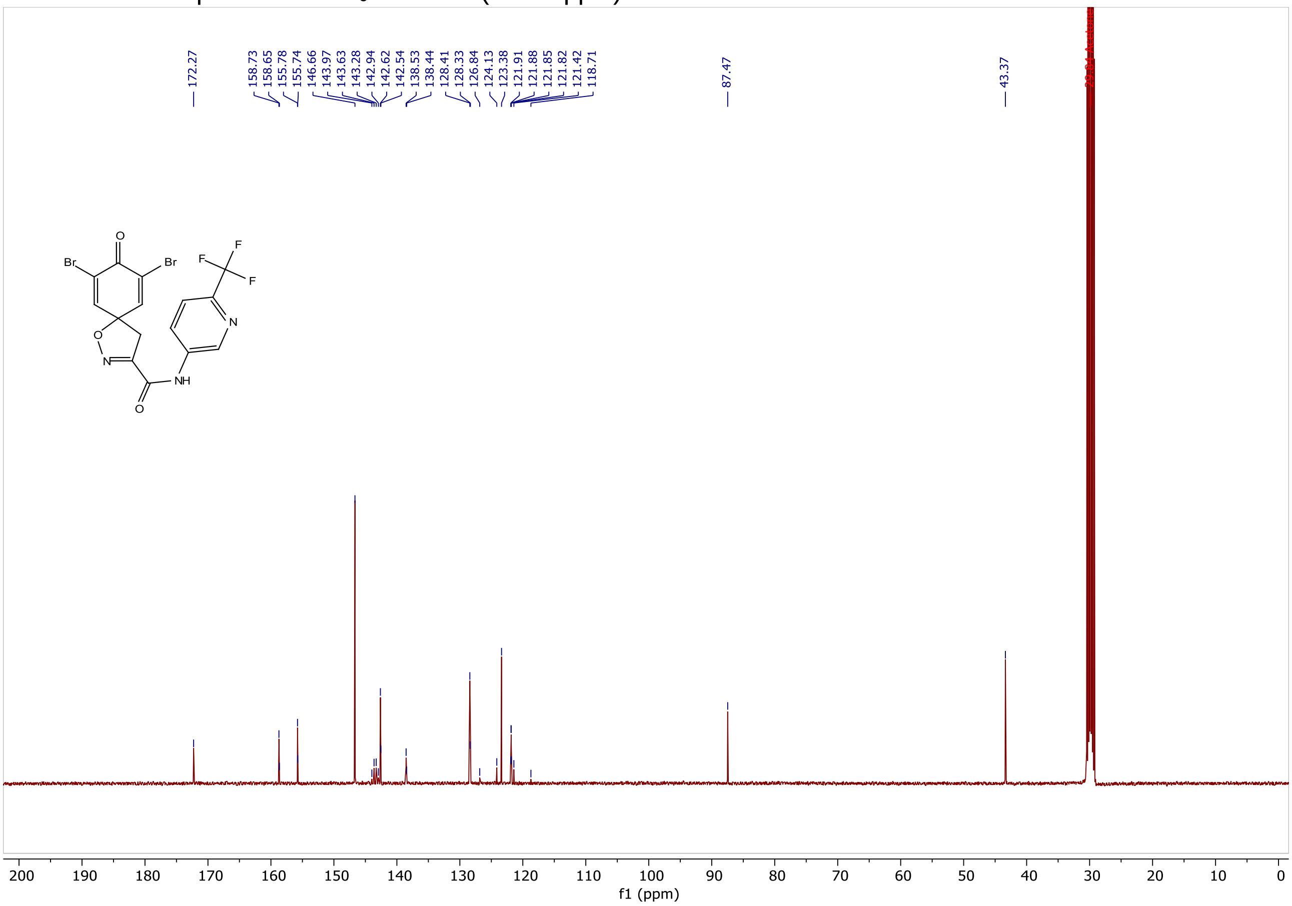

^{1}H NMR of compound **32** in $CDCl_3$ (7.26 ppm)

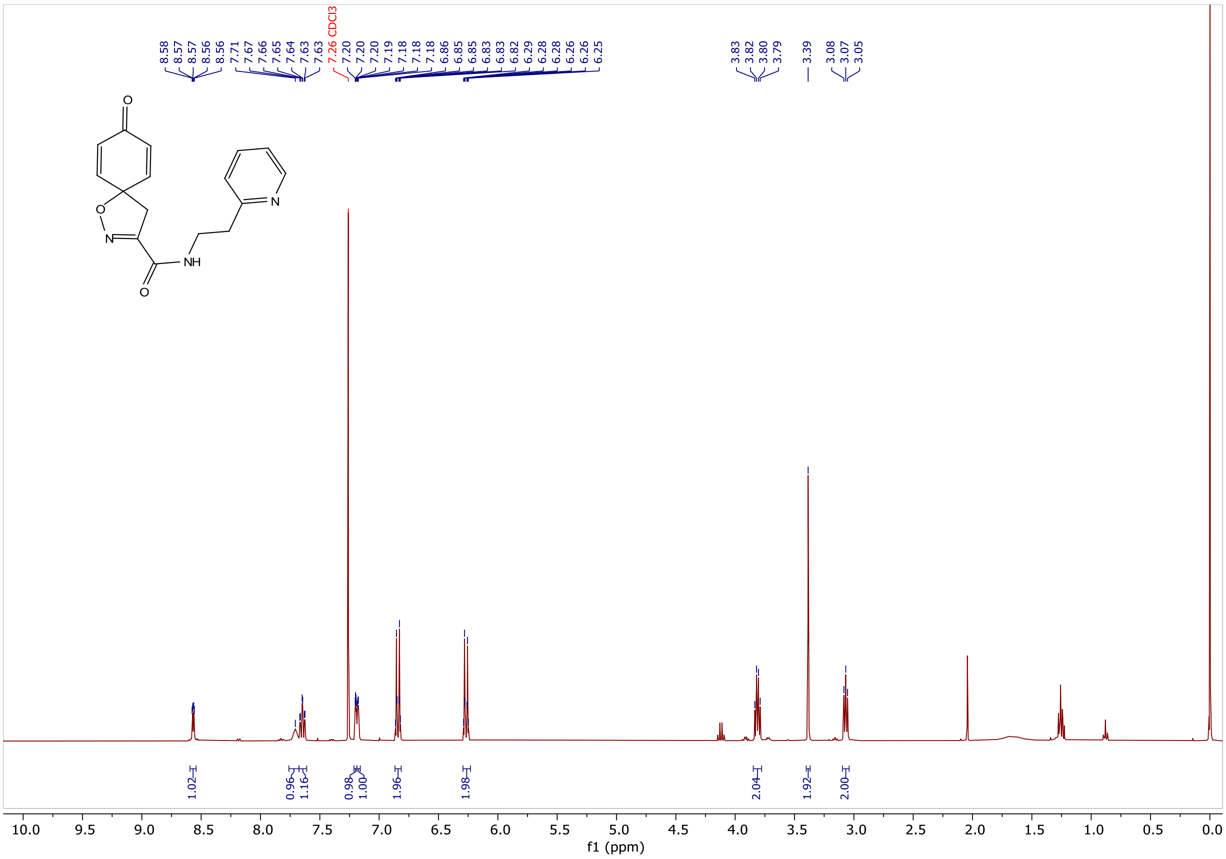

^{13}C NMR of compound **32** in $CDCl_3$ (77.16 ppm)

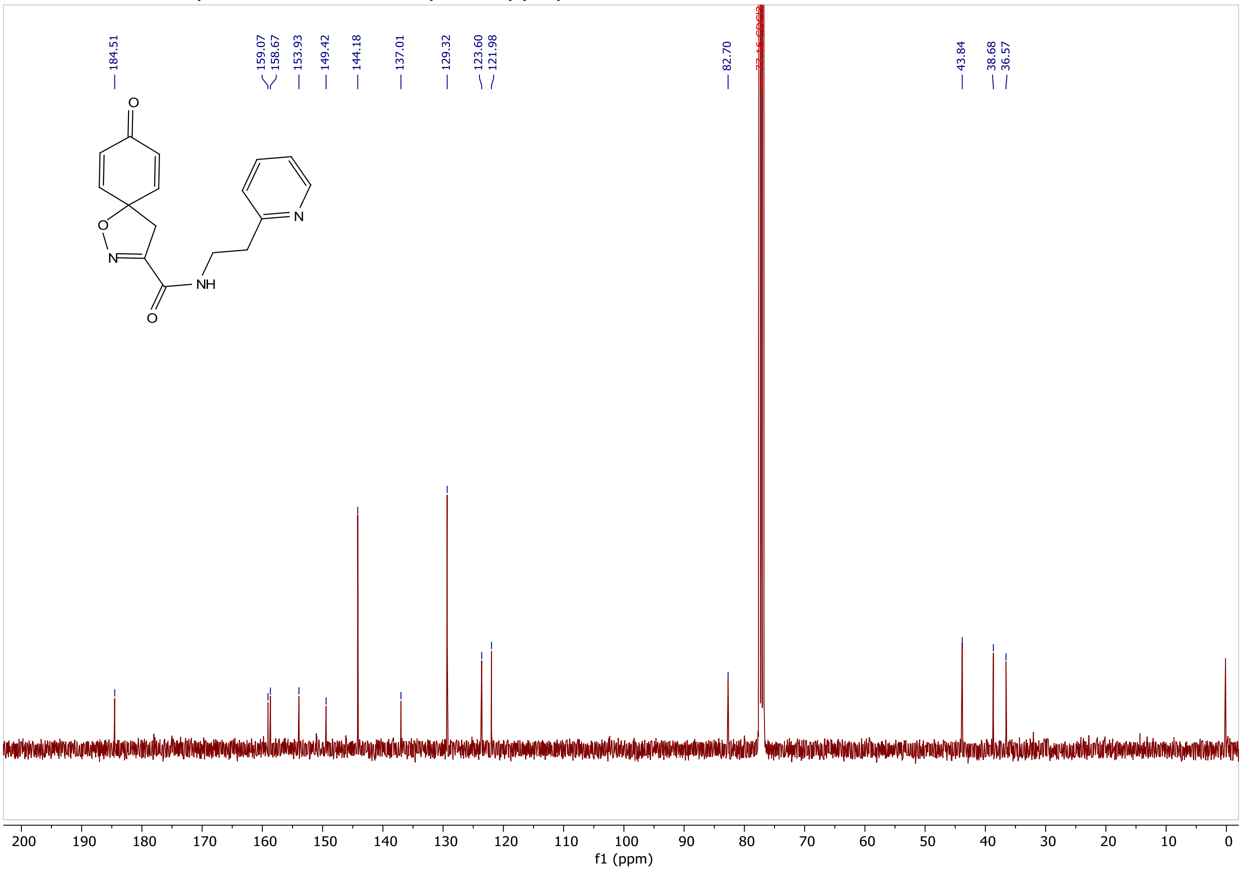

^{1}H NMR of compound **33** in $CDCl_3$ (7.26 ppm)

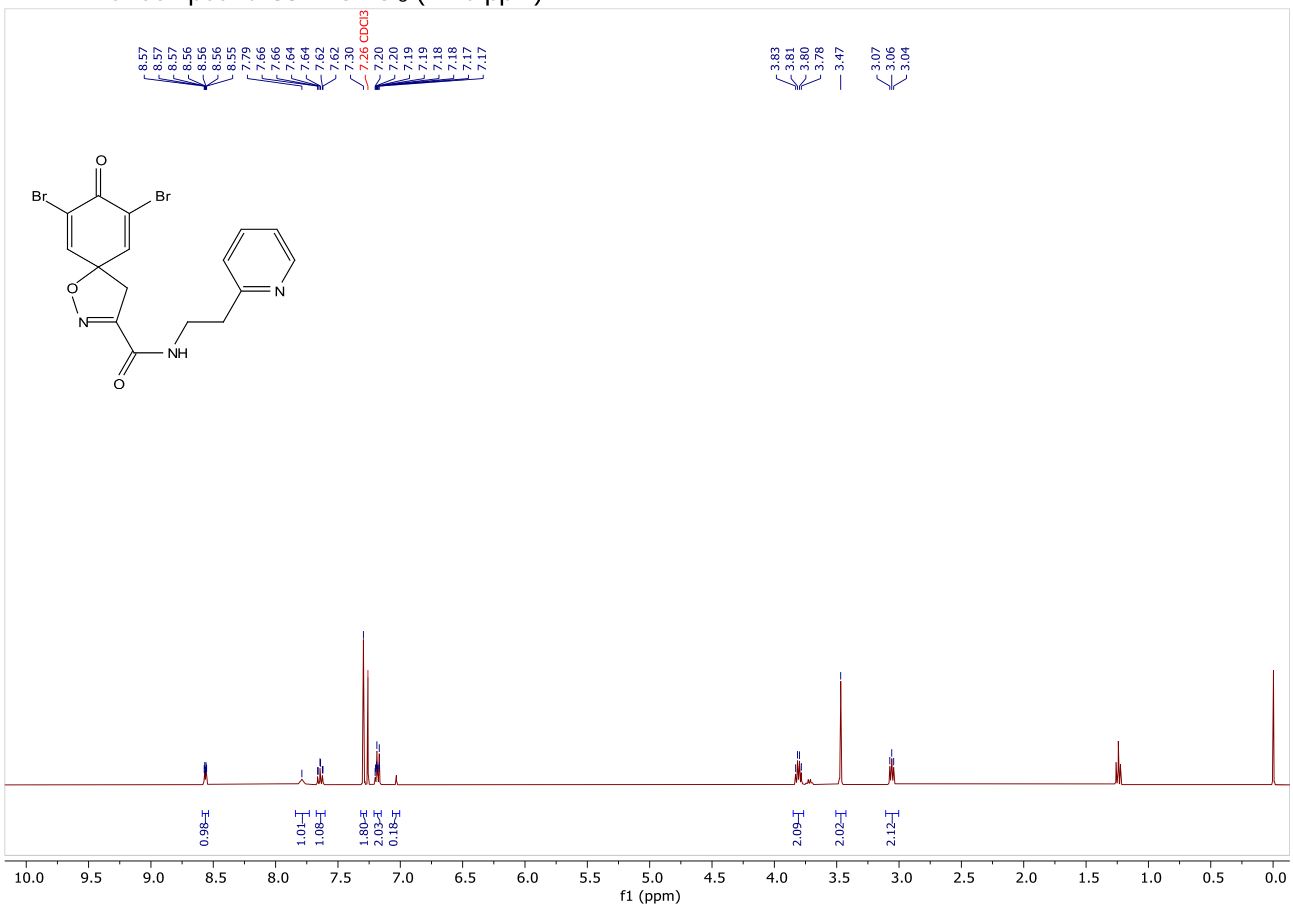

^{13}C NMR of compound **33** in $CDCl_3$ (77.16 ppm)

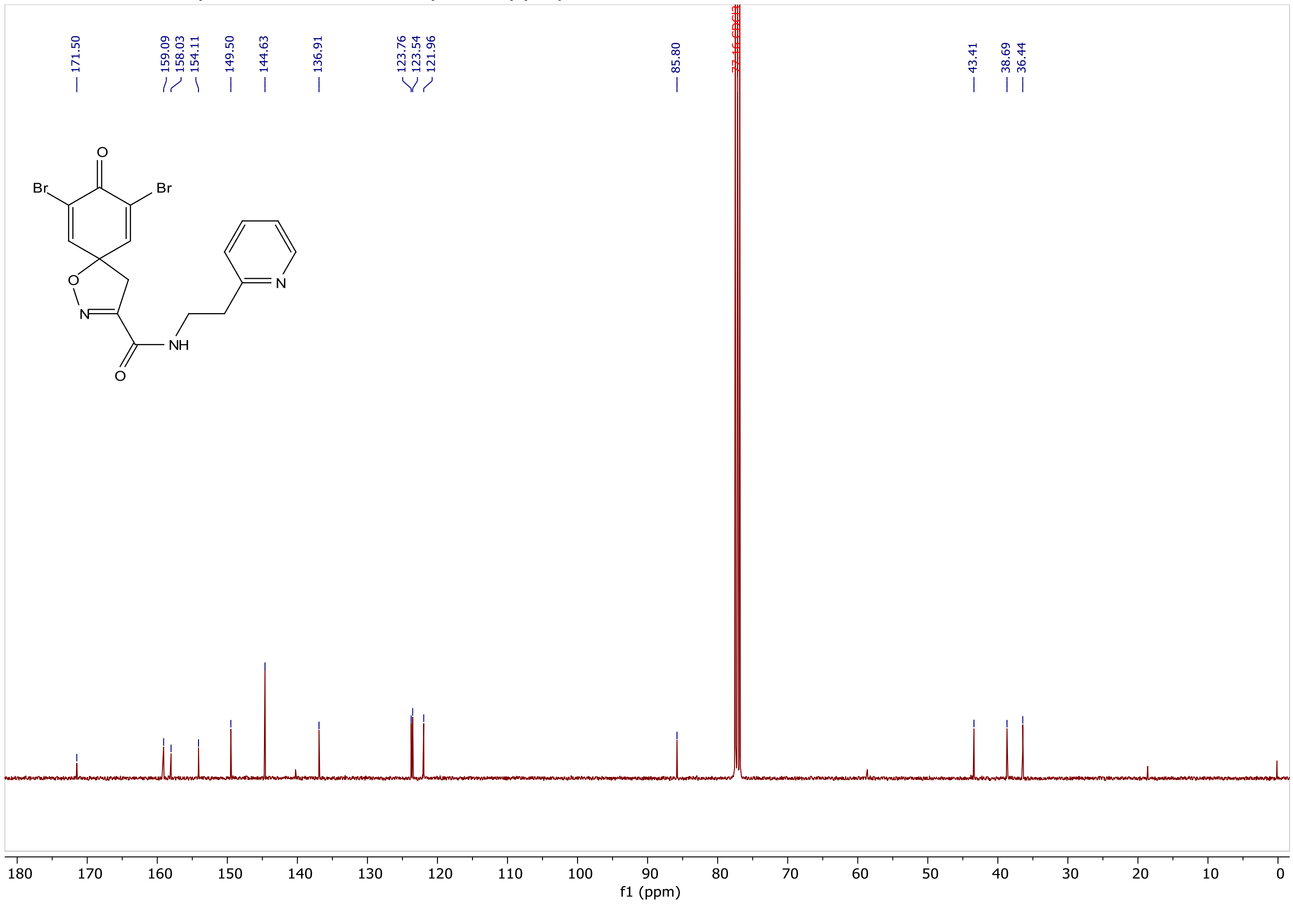

^{1}H NMR of compound **34** in d_6-DMSO (2.50 ppm)

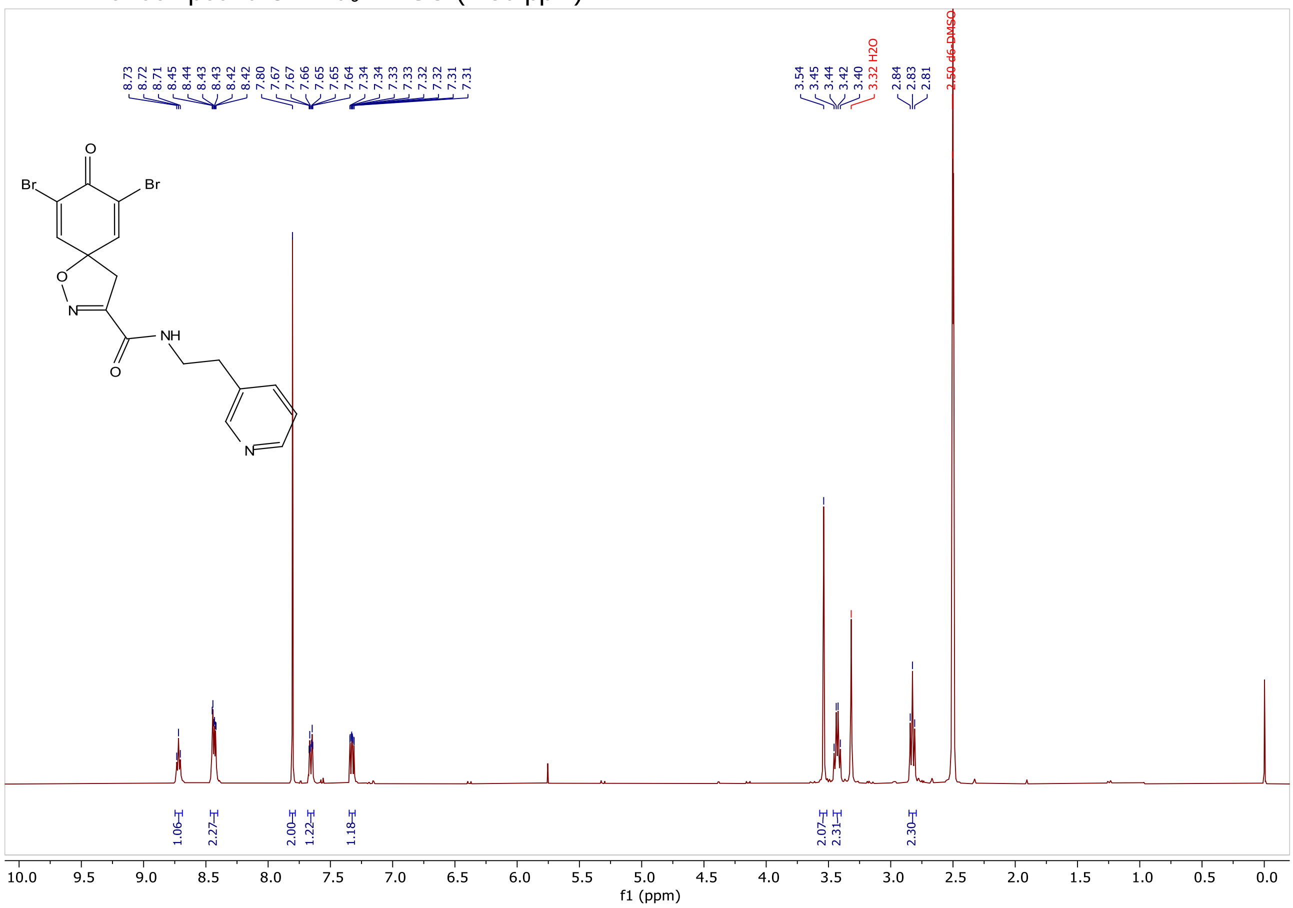

^{13}C NMR of compound **34** in d_6-DMSO (39.52 ppm)

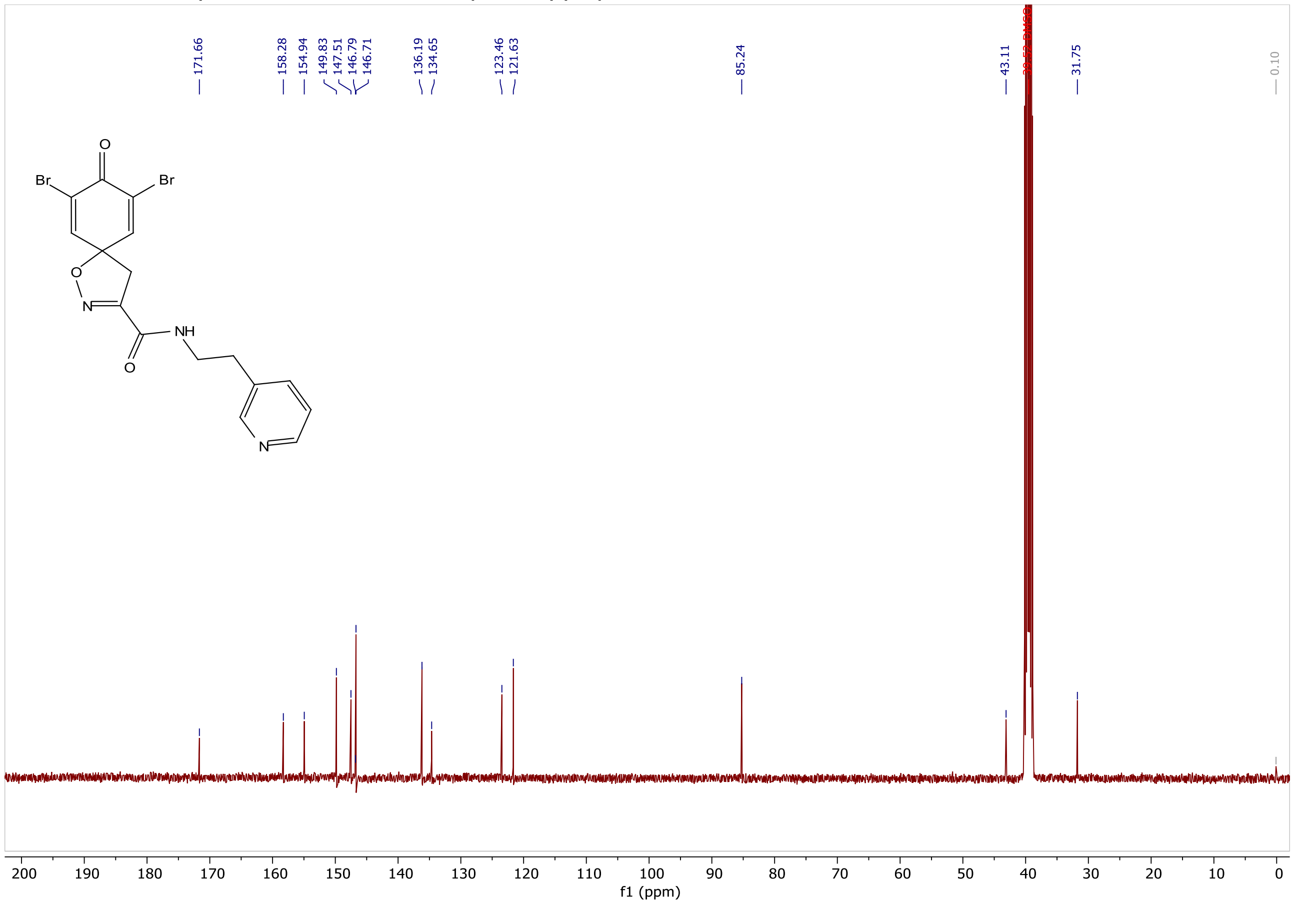

^{1}H NMR of compound **35** in d_6-acetone (2.05 ppm)

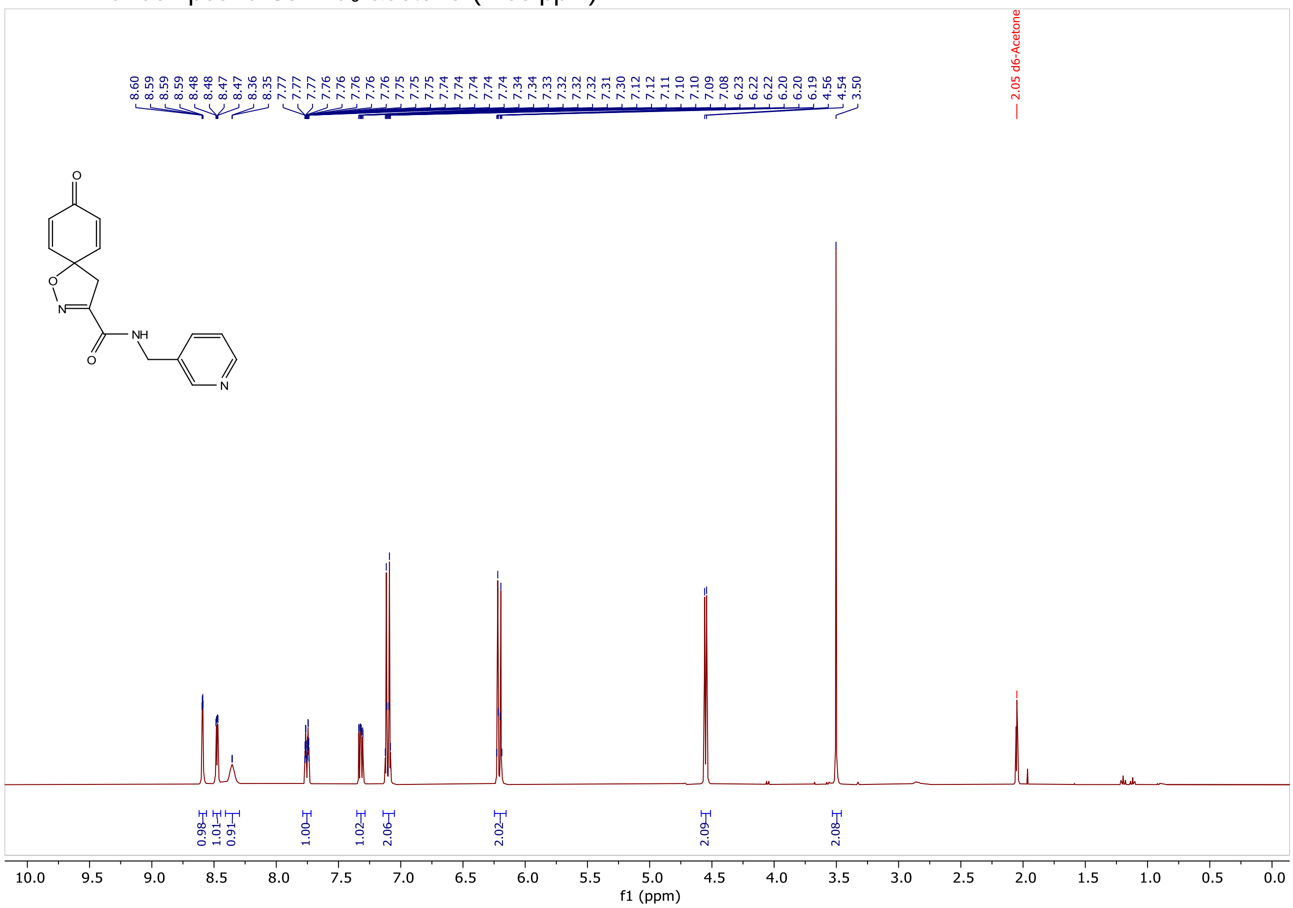

^{13}C NMR of compound **35** in d_6-acetone (29.84 ppm)

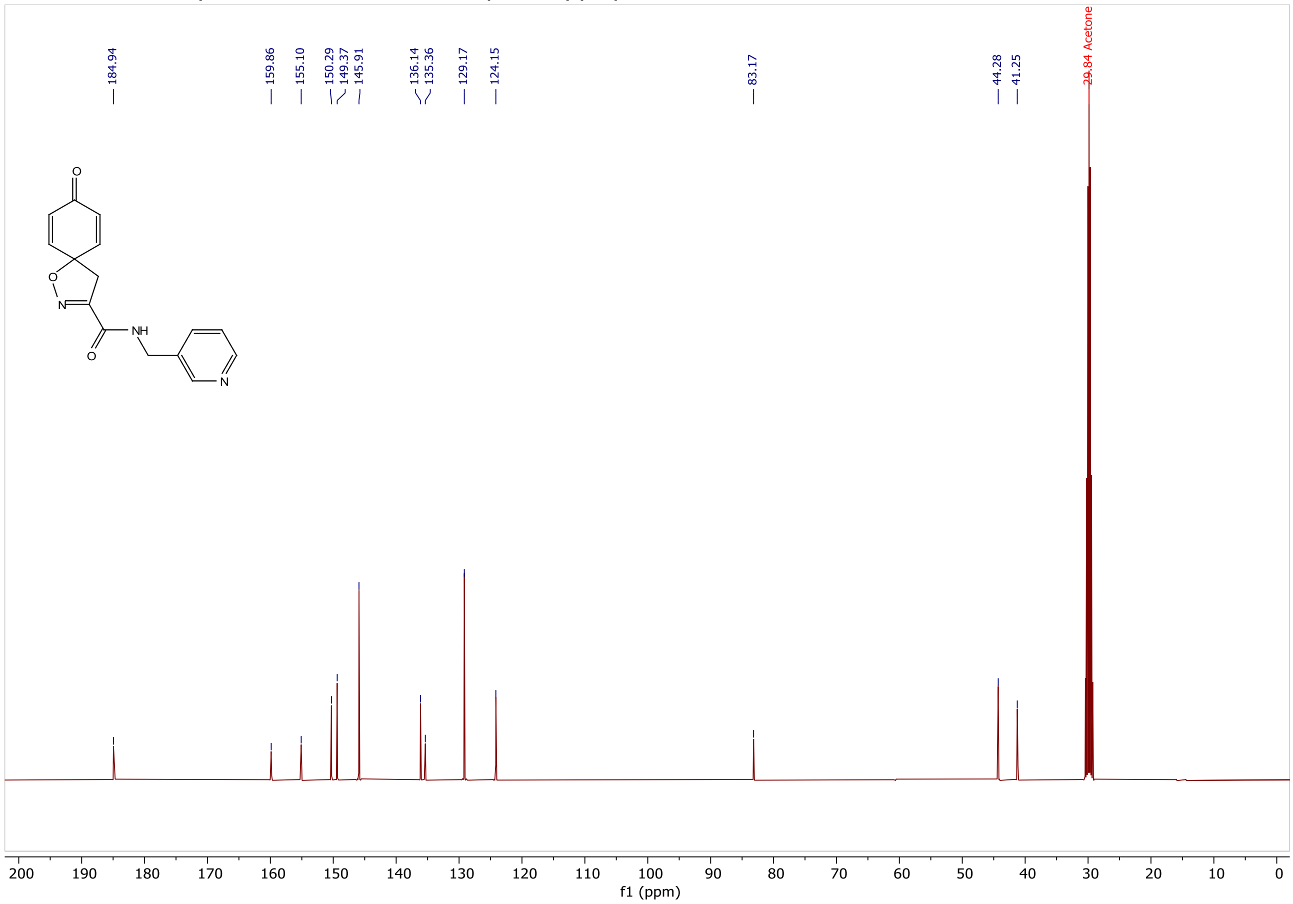

^{1}H NMR of compound **36** in d_6-DMSO (2.50 ppm)

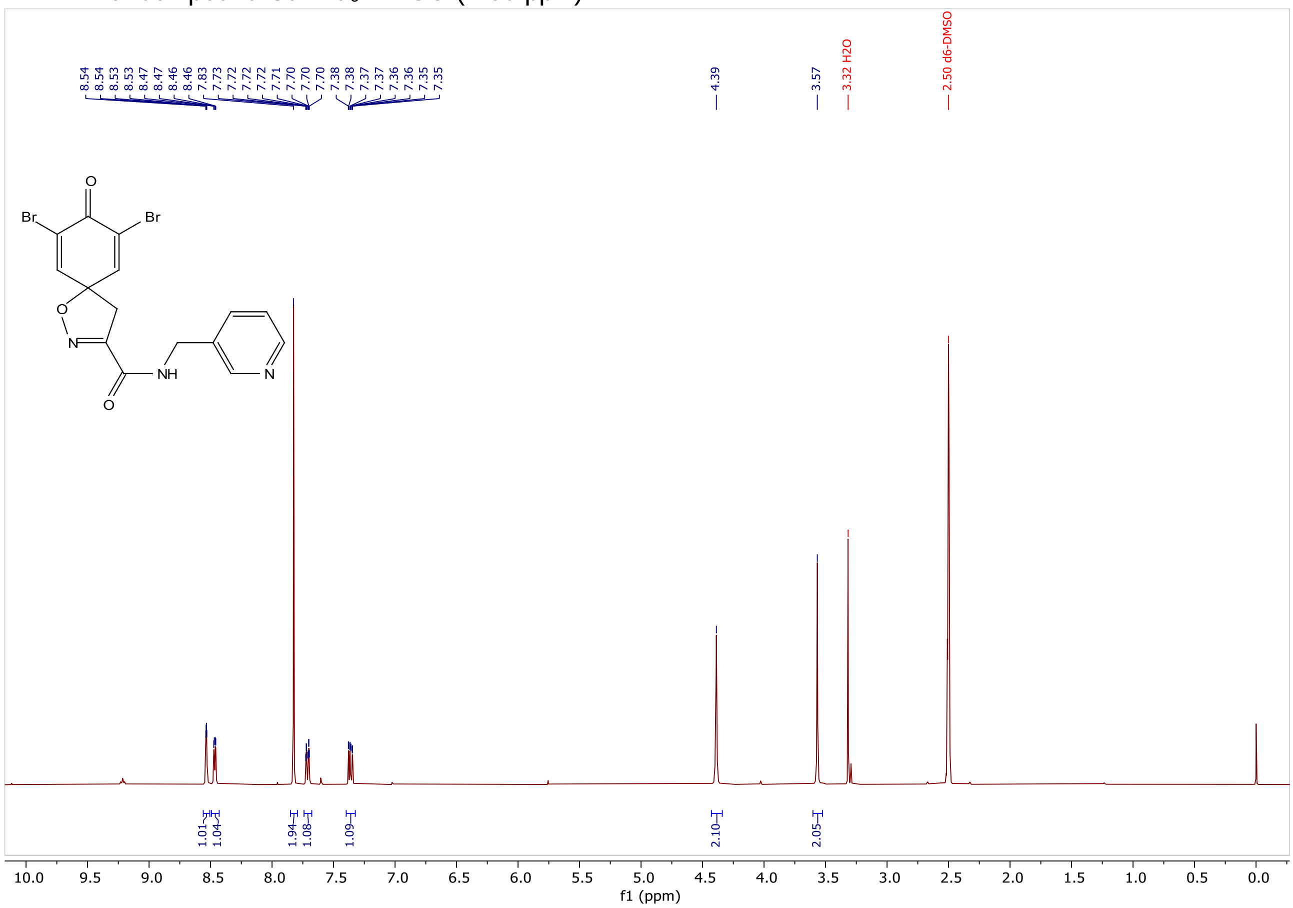

^{13}C NMR of compound **36** in d_6-DMSO (39.52 ppm)

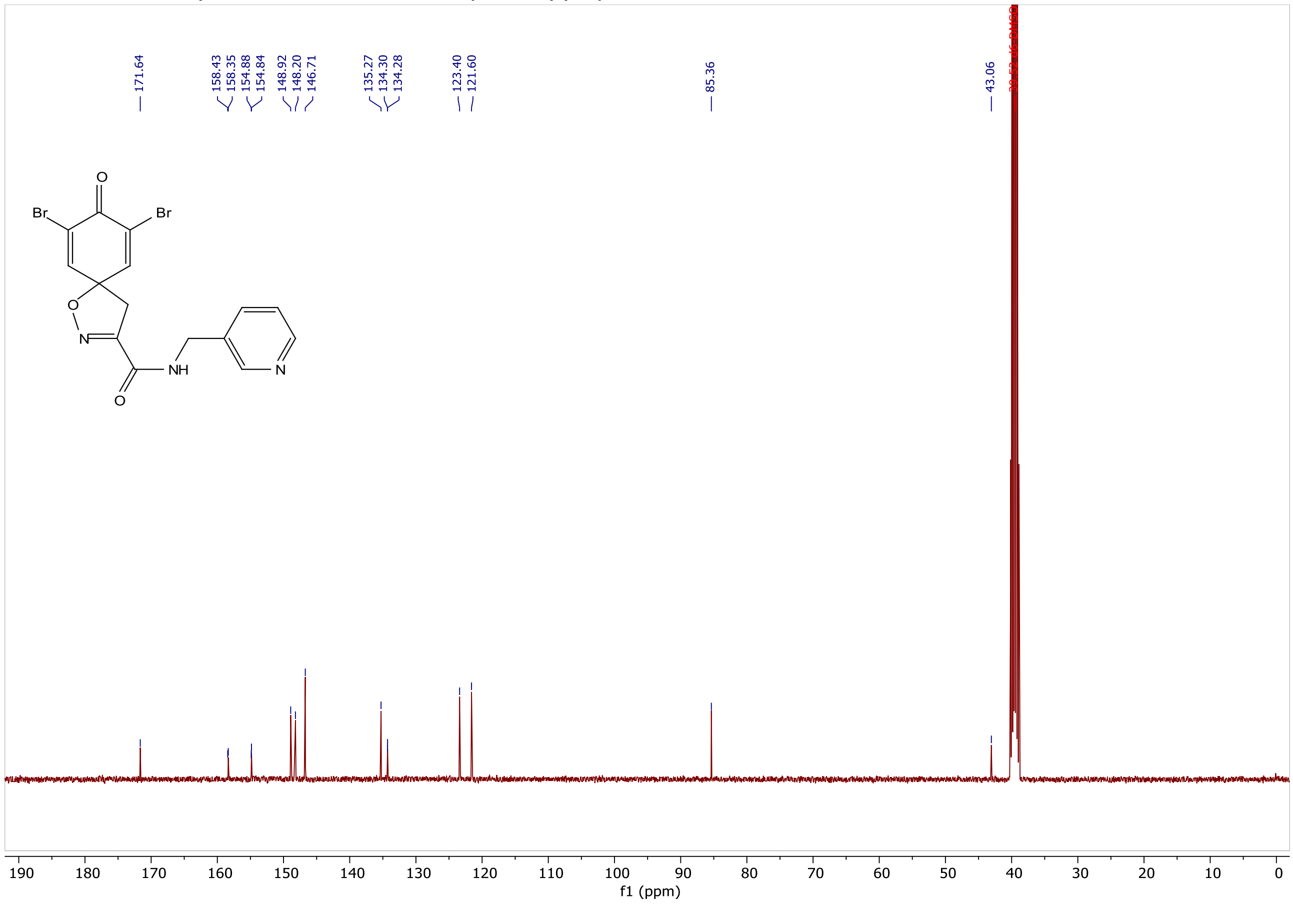

^{1}H NMR of compound **37** in *d$_6$*-DMSO (2.50 ppm)

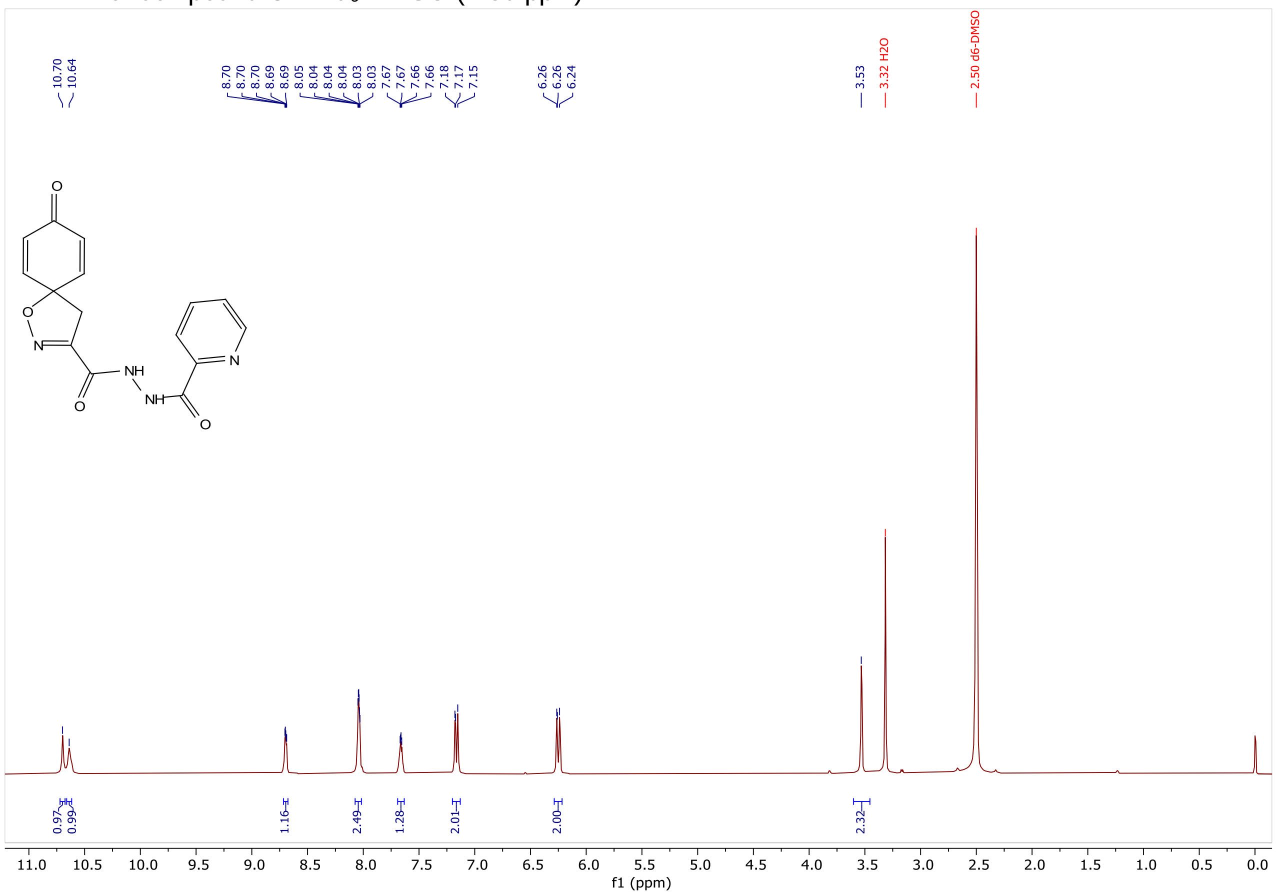

^{13}C NMR of compound **37** in *d$_6$*-DMSO (39.52 ppm)

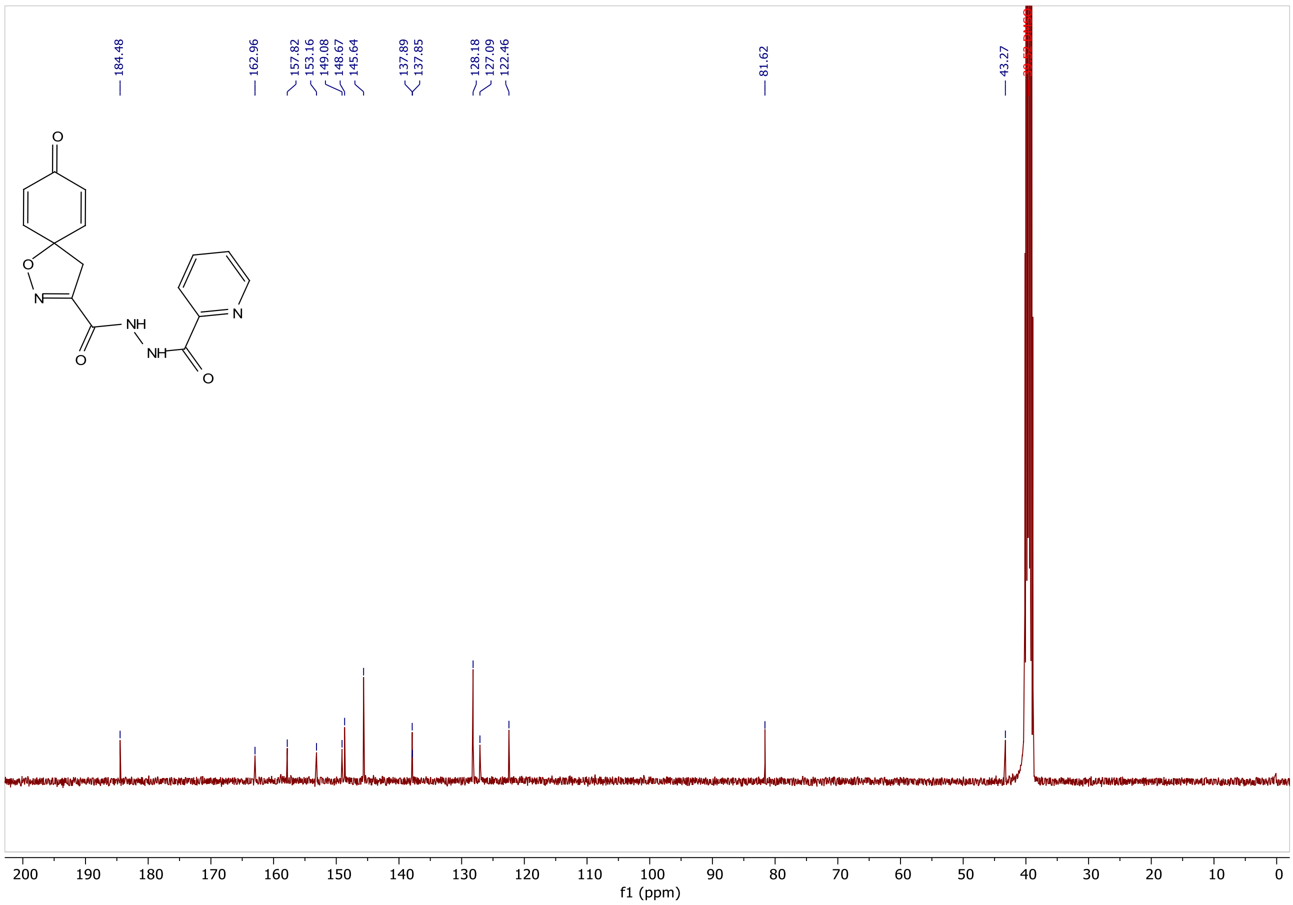

^{1}H NMR of compound **38** in d_6-DMSO (2.50 ppm)

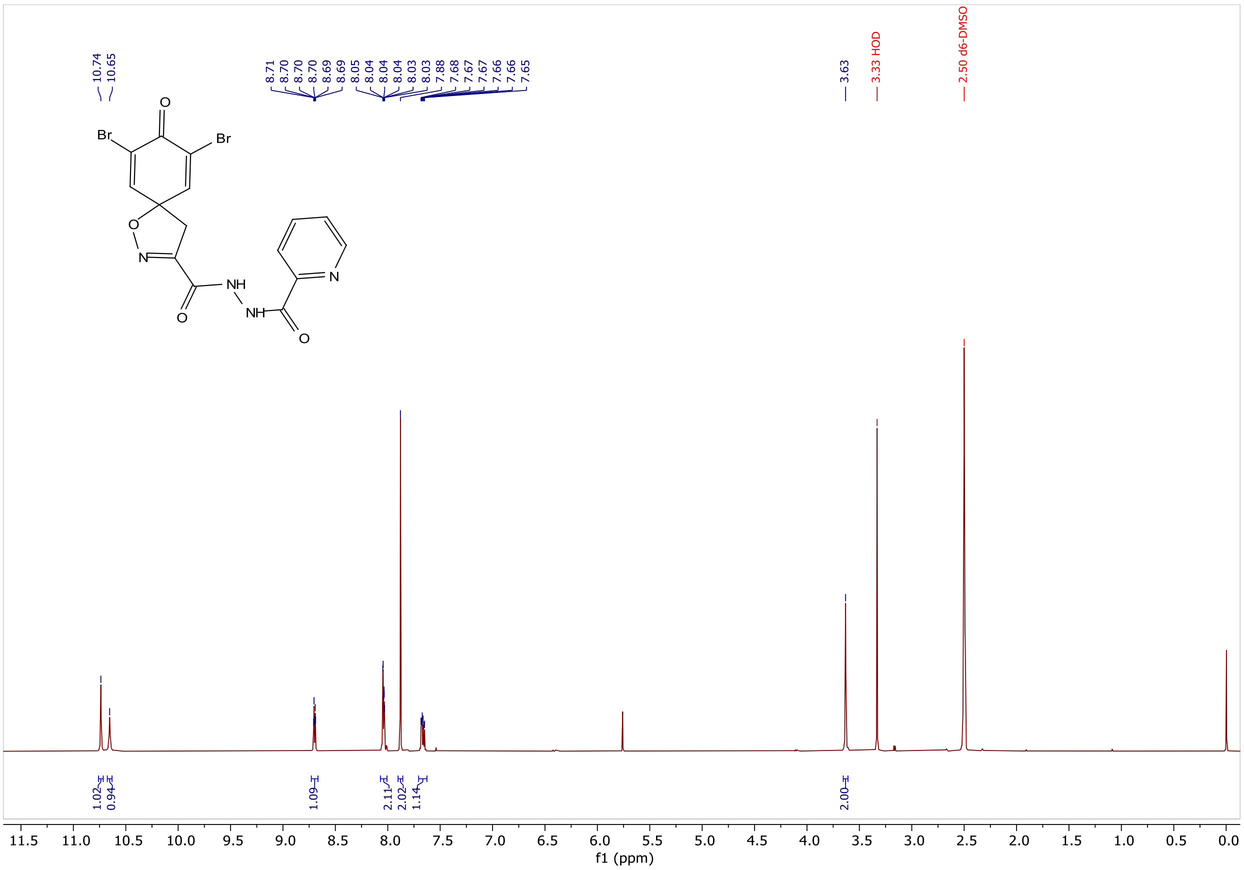

^{13}C NMR of compound **38** in d_6-DMSO (39.52 ppm)

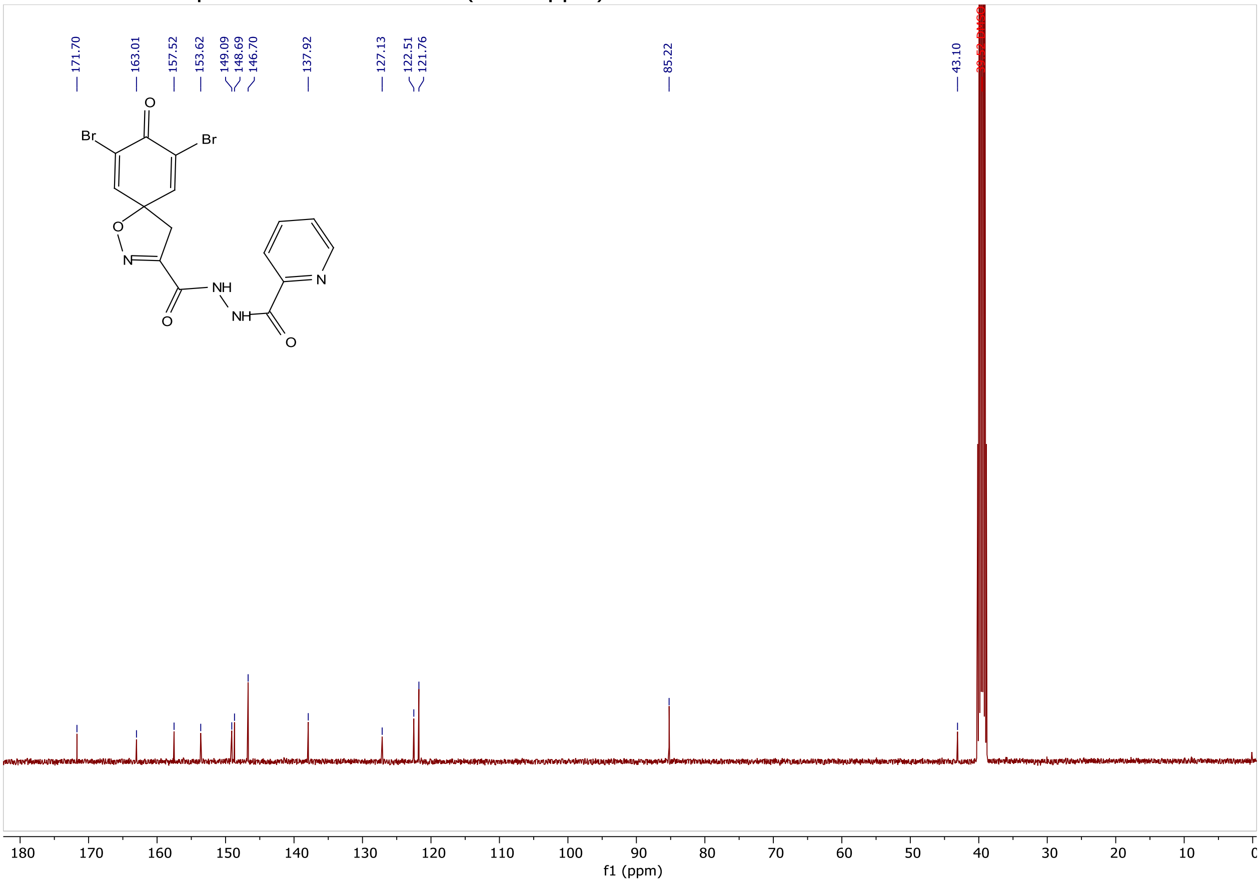

^{1}H NMR of compound **39** in d_6-DMSO (2.50 ppm)

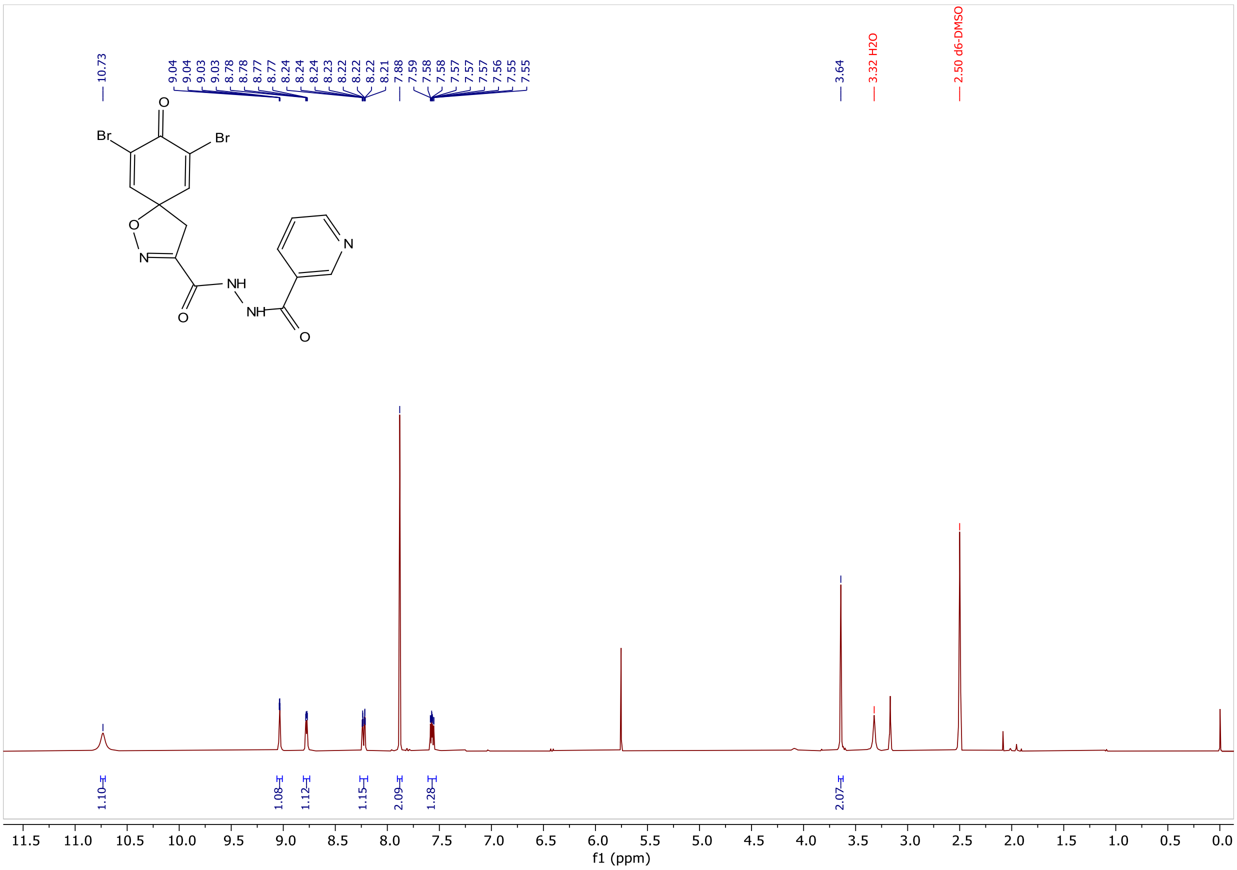

^{13}C NMR of compound **39** in d_6-DMSO (39.52 ppm)

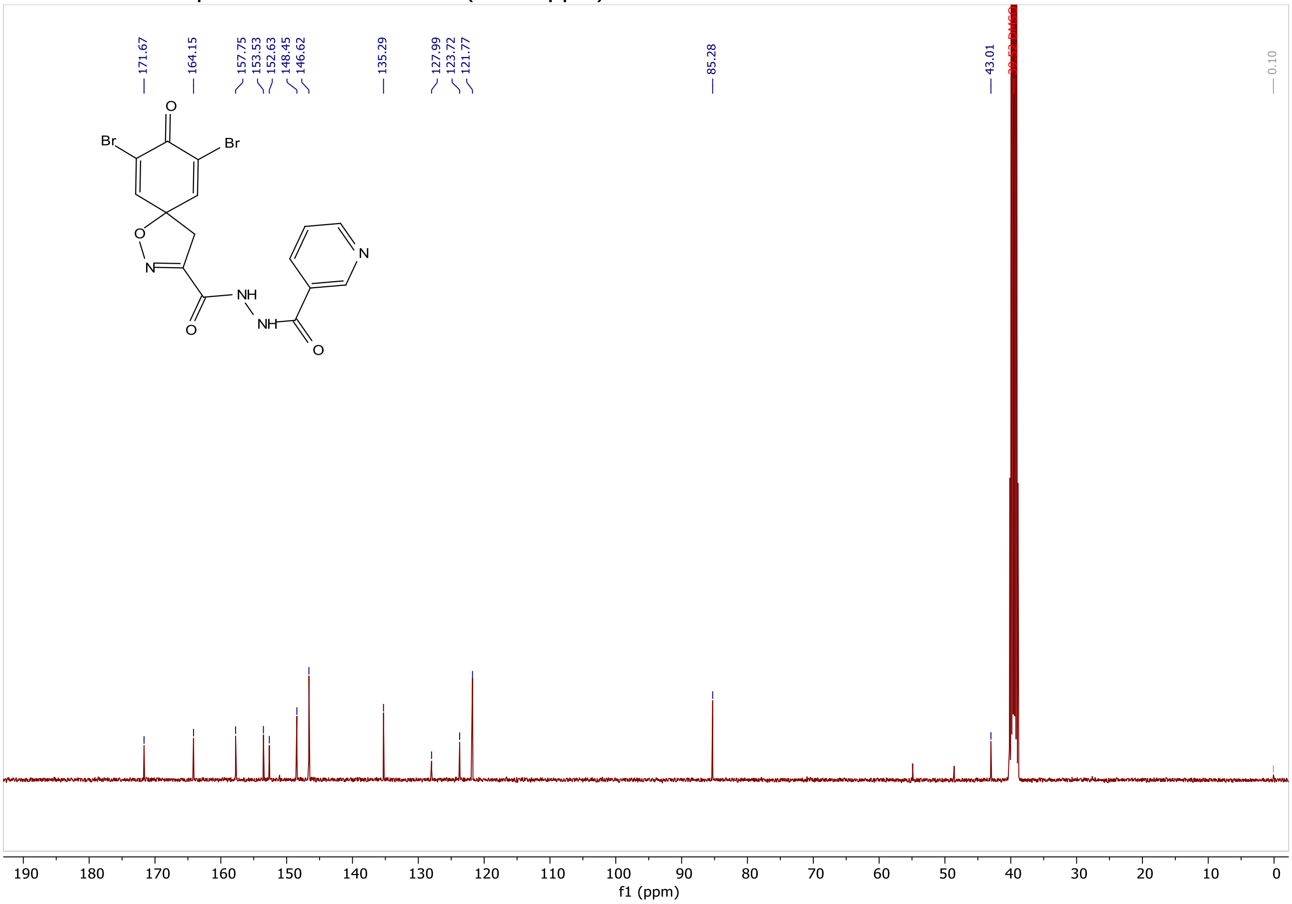

^{1}H NMR of compound **40** in $CDCl_3$ (7.26 ppm)

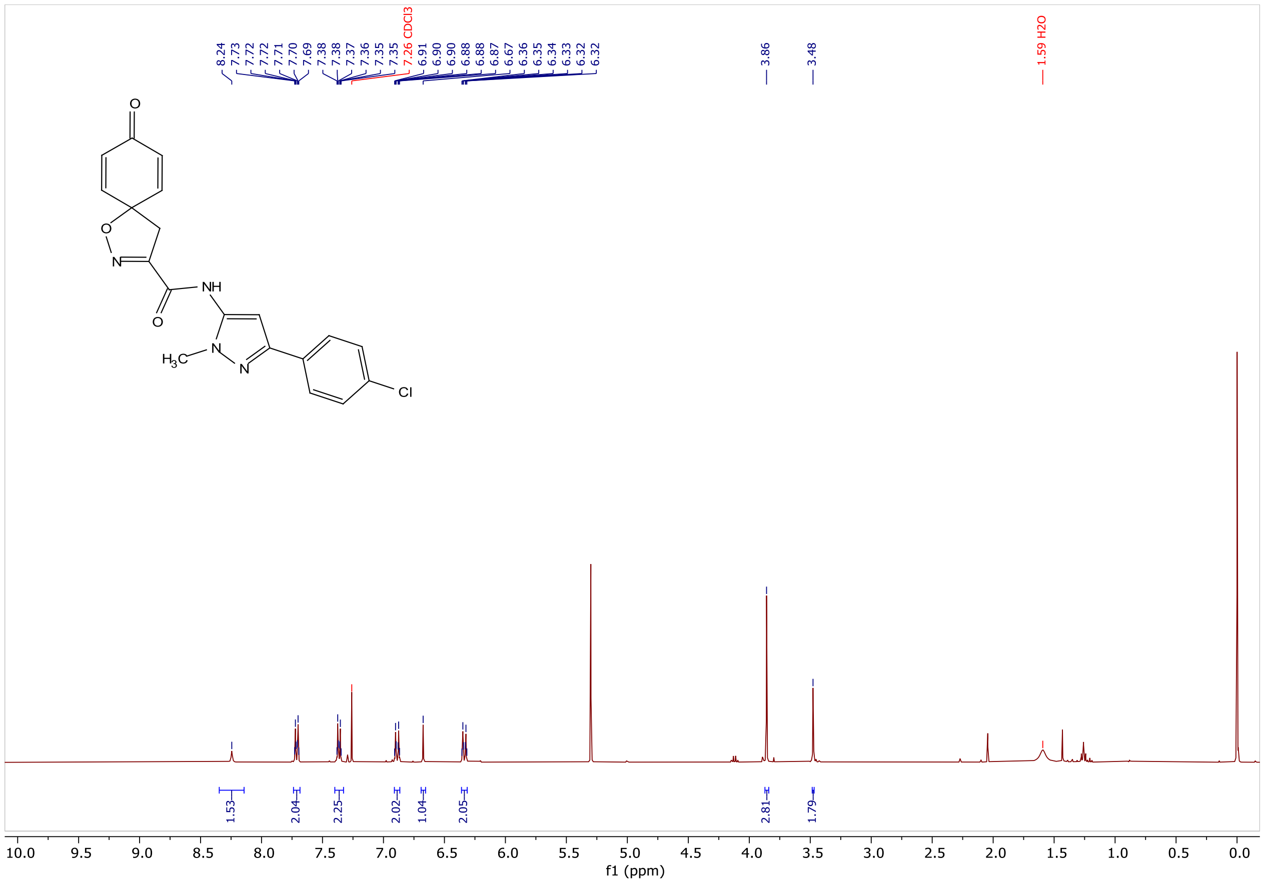

^{13}C NMR of compound **40** in $CDCl_3$ (77.16 ppm)

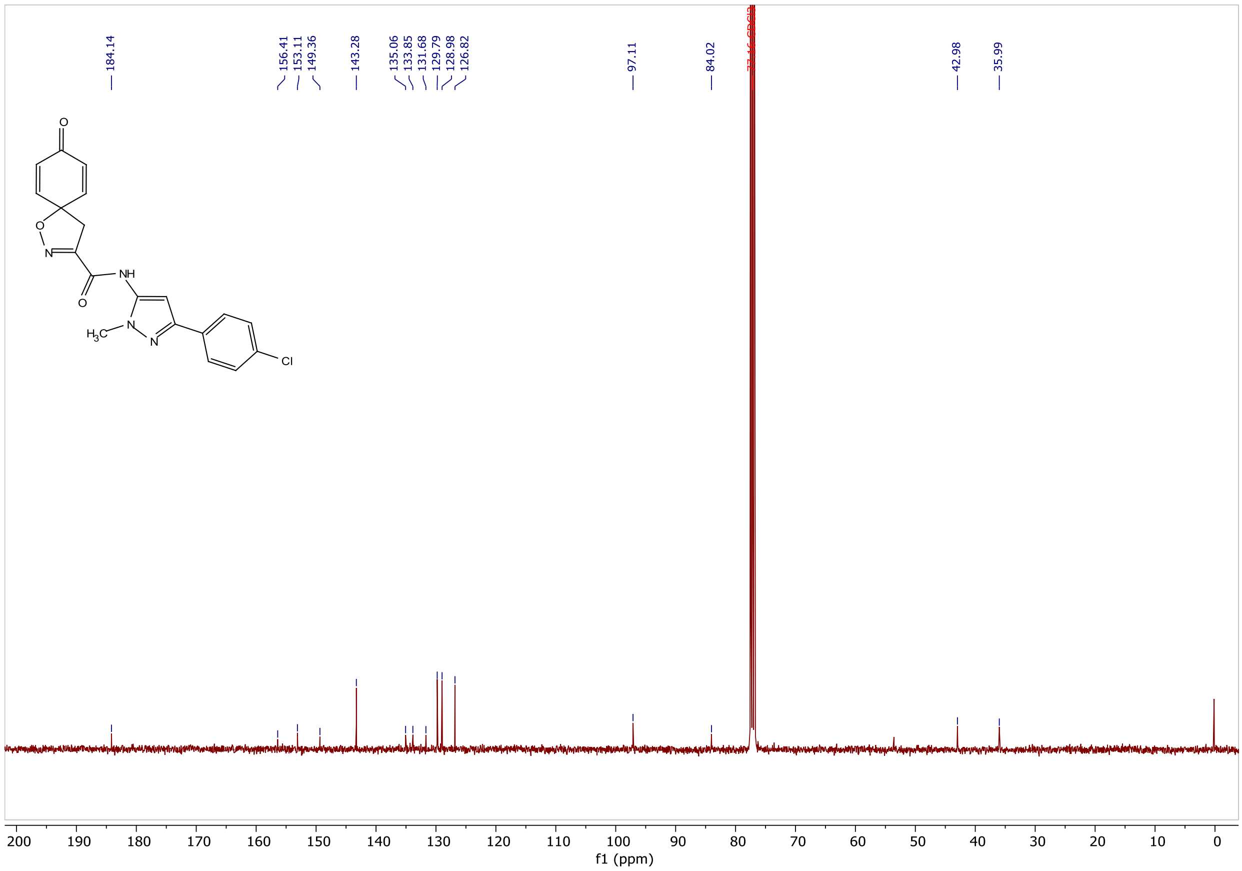

^{1}H NMR of compound **41** in d_6-DMSO (2.50 ppm)

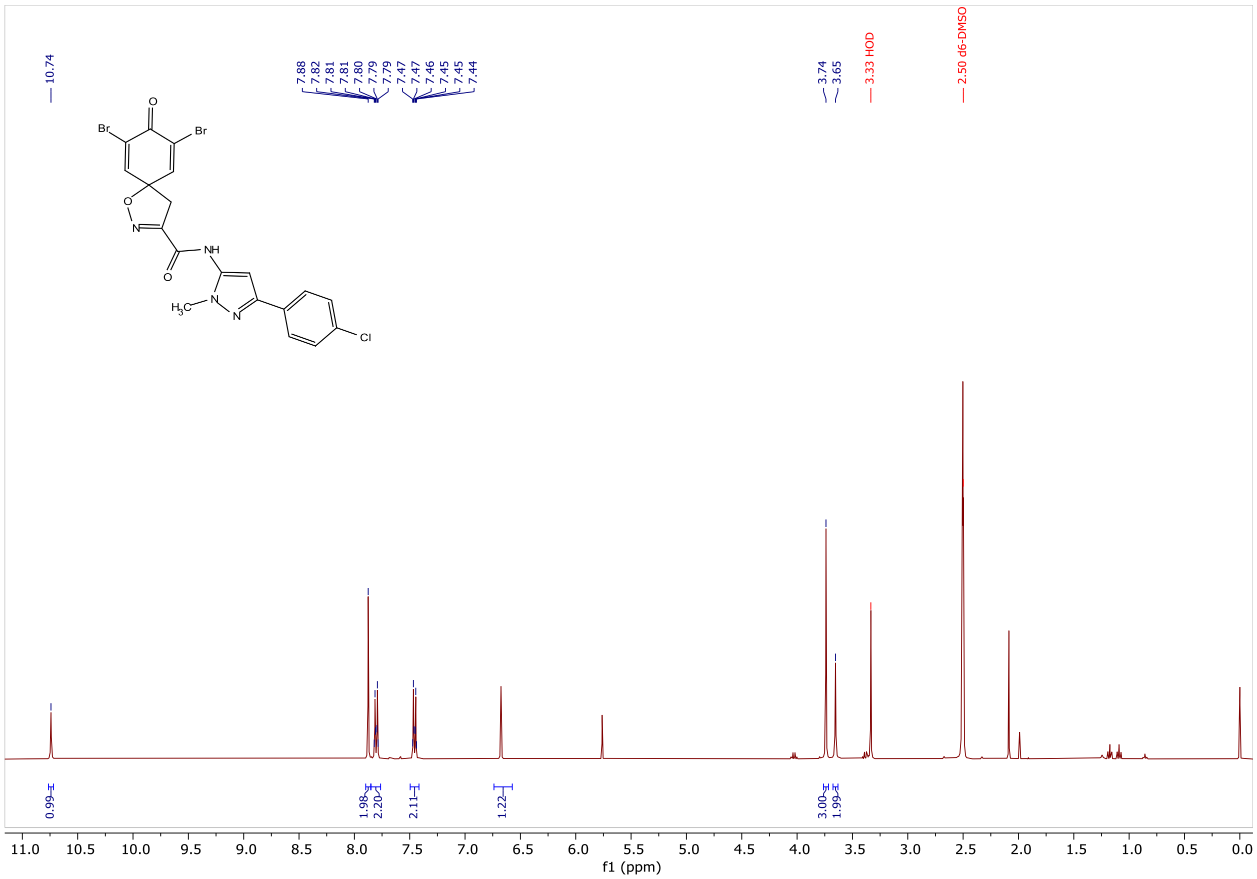

^{13}C NMR of compound **41** in d_6-DMSO (39.52 ppm)

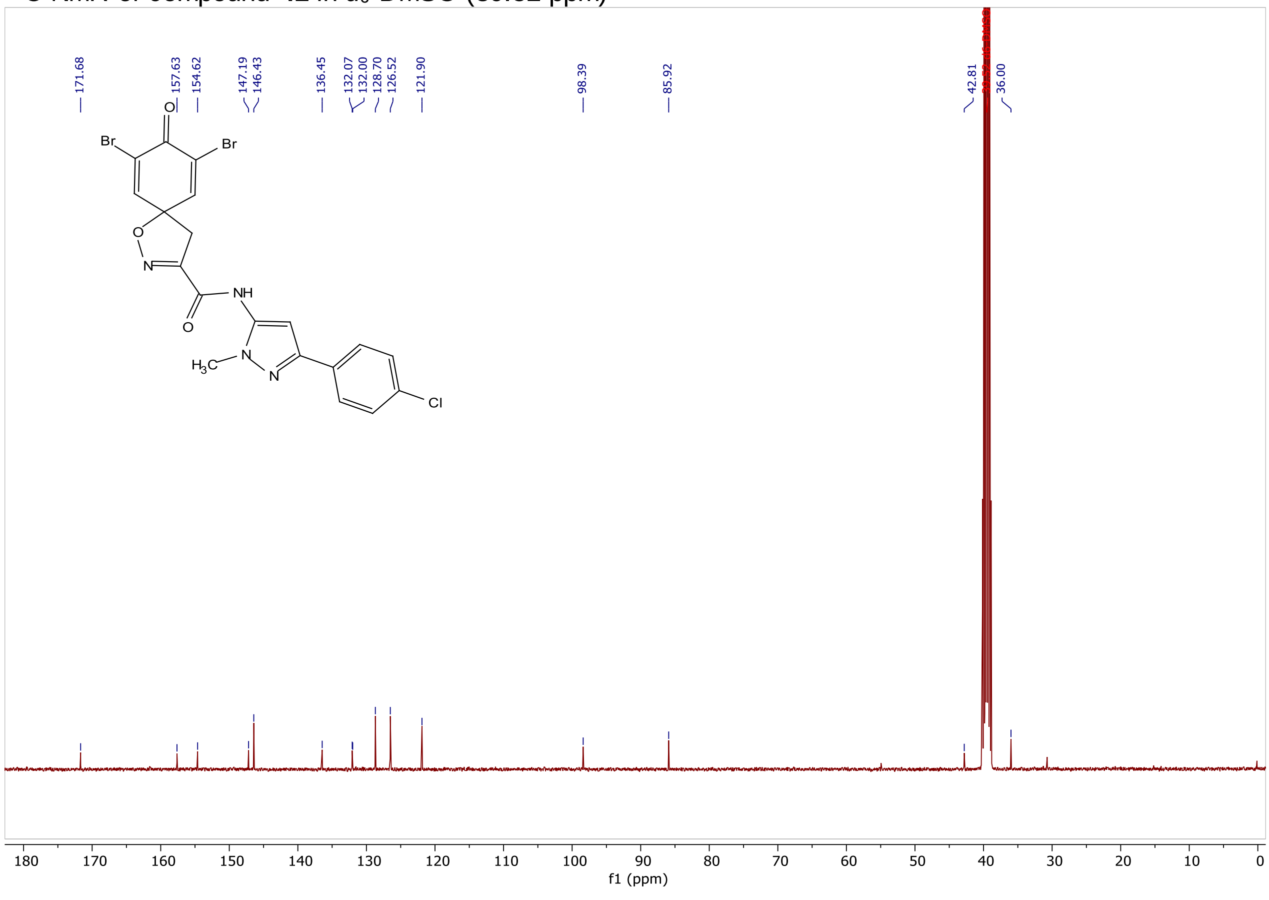

^{1}H NMR of compound **42** in d_6-acetone (2.05 ppm)

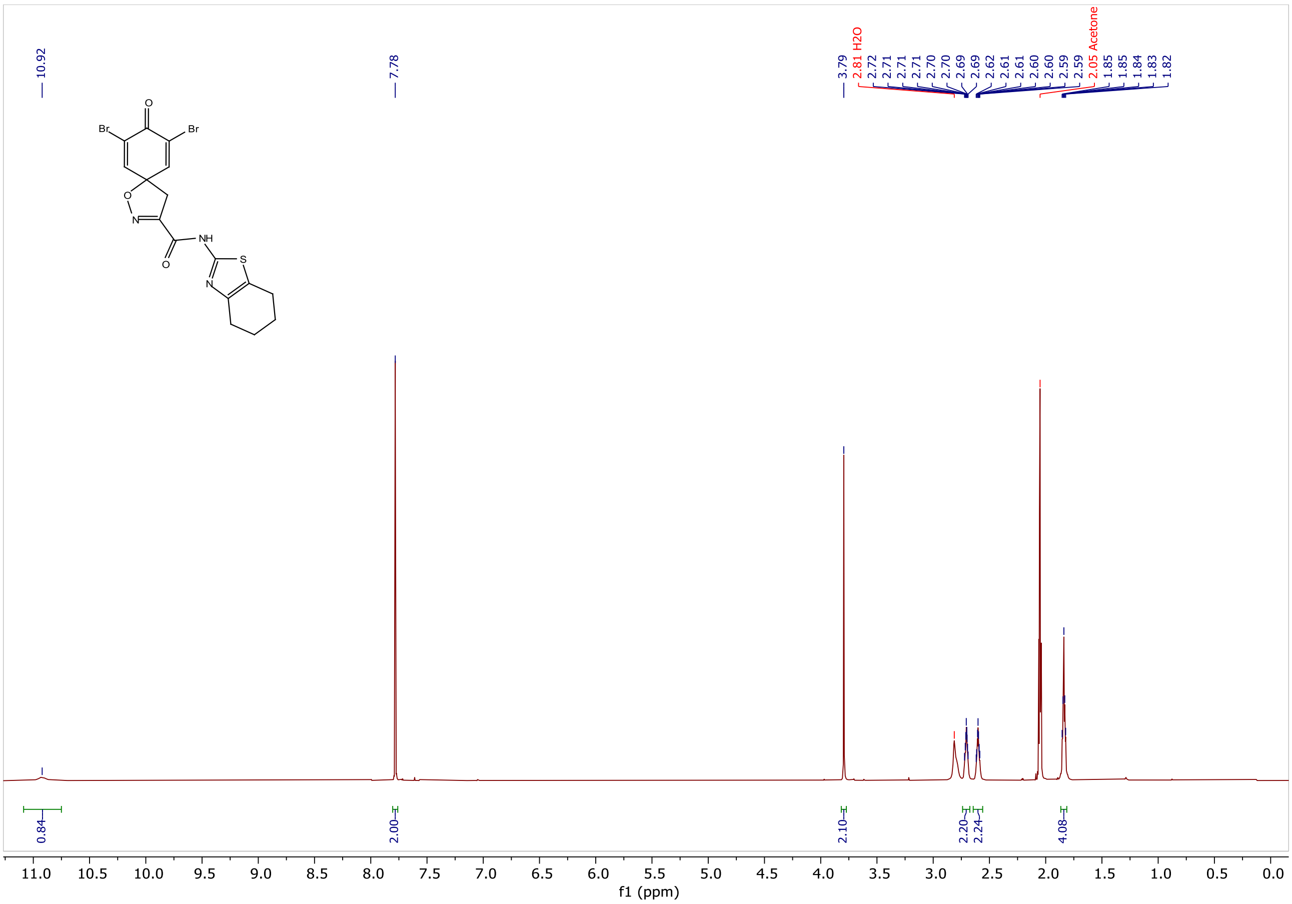

^{13}C NMR of compound **42** in d_6-acetone (29.84 ppm)

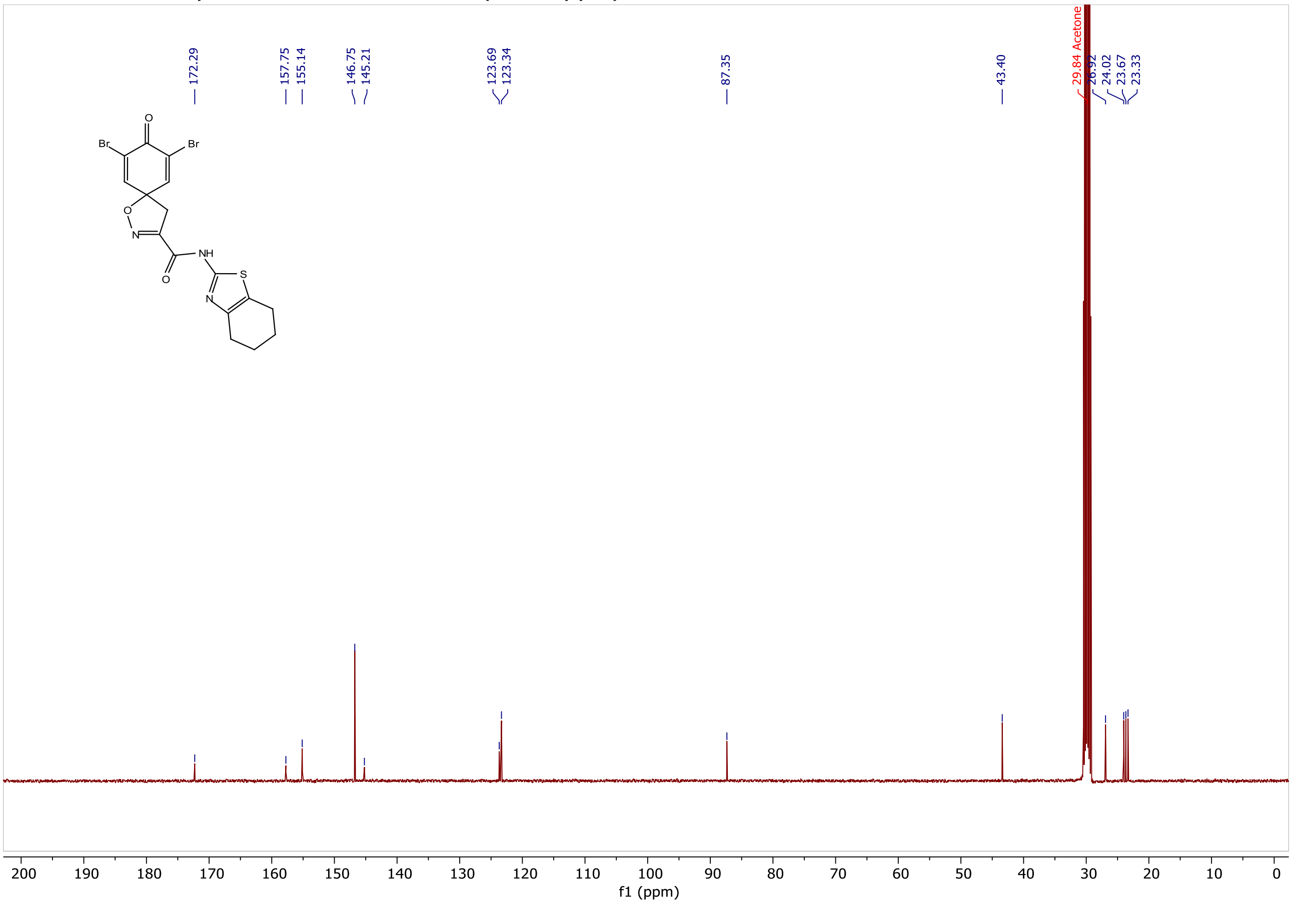

Supplement: Supplementary file 1 [file marinedrugs-19-00400-s001.zip › marinedrugs-1123322-supplementary.pdf]
